# Supplementary material for: Plasma lipopolysaccharide levels predict mortality in acutely ill children in Low- and Middle-Income Countries
Source: Nat Commun. 2025 Nov 28;16:10787. doi: 10.1038/s41467-025-65429-0 (PMC12663156; doi:10.1038/s41467-025-65429-0)
Supplement: Supplementary file 1 — Supplementary Information [file 41467_2025_65429_MOESM1_ESM.pdf]

SUPPLEMENTARY MATERIAL

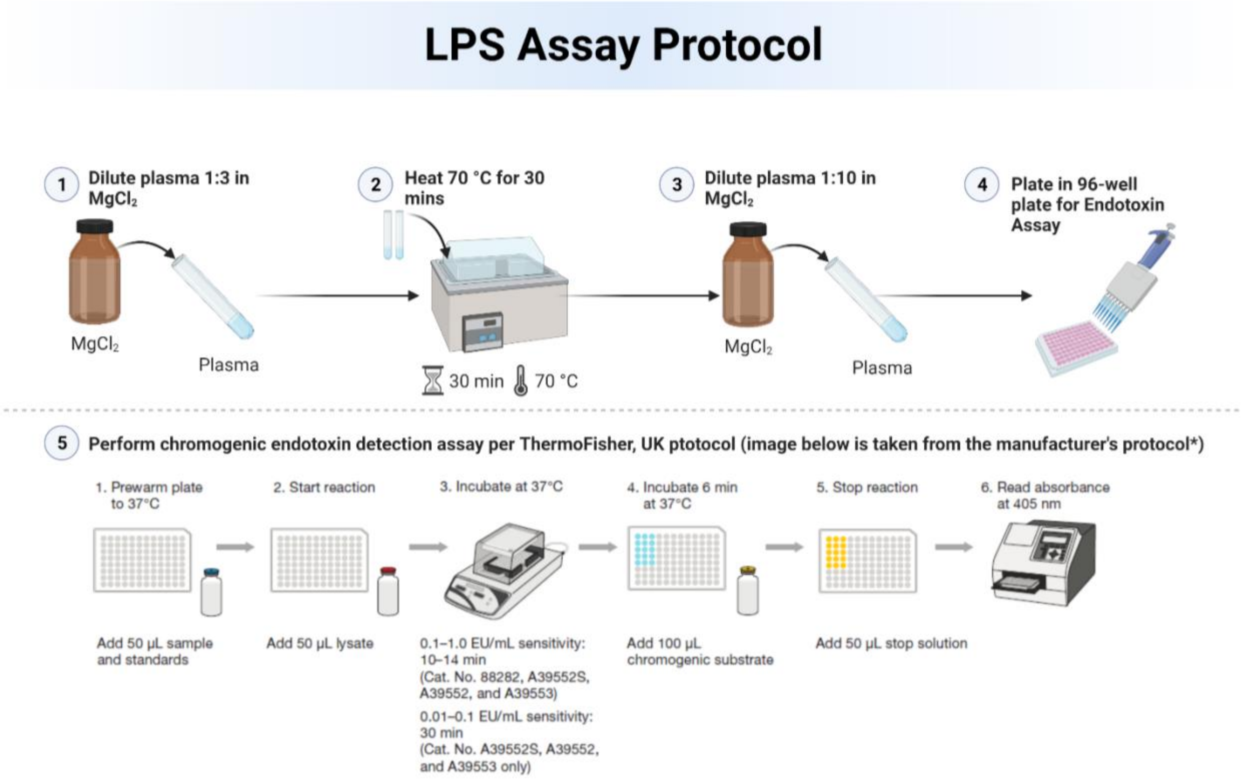

Supplementary Methods 1: Optimization of LPS chromogenic assay

The ThermoFisher, UK chromogenic endotoxin detection assay was optimized (steps 1-4) to assess LPS levels in plasma samples collected from CHAIN participants. Optimization included dilutions with MgCl2 to prevent EDTA-driven inhibition of the assay and heating steps to destabilize interfering proteins. Created in BioRender. Allen, C. (2025) https://BioRender.com/c7w1e9o

Supplementary Methods 2: Cohort and sub-group definitions

Cohorts were defined as follows:

- Admission cohort: All children hospitalized at index visit (day 0).
- Discharge cohort: Admission cohort participants discharged from hospital during study period (November 2016 and January 2019).
- Community control cohort: Similarly, aged well-children from the same community as admission participants who were not hospitalized at index visit.
- '*90-day-death'* cohort: Admission participants who died within 90 days of hospitalization.
- '*Post-90-day-death*' cohort: Admission participants who survived 90-day interval after hospitalization but died during study period.
- '*Alive*' cohort: Admission participants who were alive at study end.

LPS-mortality sub-groups

- *LPS^{hi} Fatal (F) (n = 40)* – Admission participants who presented with plasma LPS > 11.58 E.U./ml and died within 90 days of index visit.
- *LPS^{lo} Fatal (F) (n= 125)* – Admission participants who presented with plasma LPS ≤ 11.58 E.U./ml and died within 90 days of index visit.
- *LPS^{hi} Non-Fatal (NF) (n= 46)* – Admission participants who presented with plasma LPS > 11.58 E.U./ml and were alive 90 days post index visit.
- *LPS^{lo} Non-Fatal (NF) (n = 427)* – Admission participants who presented with plasma LPS ≤ 11.58 E.U./ml and were alive 90 days post index visit.

Packages	References
Base R	R Core Team. R: A Language and Environment for Statistical Computing. R Foundation for Statistical Computing 2022; Vienna, Austria. https://www.R-project.org/.
AICcmodavg	Mazerolle MJ (2023). _AICcmodavg: Model selection and multimodel inference based on (Q)AIC(c)_. R package version 2.3.2, https://cran.r-project.org/package=AICcmodavg.
CCPlotR	Sarah Ennis, Pilib Ó Broin, Eva Szegezdi (2023). CCPlotR: an R package for the visualization of cell–cell interactions, *Bioinformatics Advances*, Volume 3, Issue 1, 2023, vbad130, https://doi.org/10.1093/bioadv/vbad130
ComplexHeatmap	Gu, Z. (2016) Complex heatmaps reveal patterns and correlations in multidimensional genomic data. Bioinformatics.
contsurvplot	Denz R, Timmesfeld N (2023). “Visualizing the (Causal) Effect of a Continuous Variable on a Time-To-Event Outcome.” Epidemiology, 34(5).
corrplot	Wei T, Simko V. R package ‘corrplot’: Visualization of a Correlation Matrix. (Version 0.92). 2021; published online Nov 18. https://github.com/taiyun/corrplot (accessed June 28, 2023).
ggcorrplot	Kassambara A (2023). _ggcorrplot: Visualization of a Correlation Matrix using 'ggplot2'_. R package version 0.1.4.1, https://CRAN.R-project.org/package=ggcorrplot
glmnet	Tay JK, Narasimhan B, Hastie T. Elastic Net Regularization Paths for All Generalized Linear Models. 2021; published online March 5. http://arxiv.org/abs/2103.03475 (accessed Jan 26, 2023).
gtsummary	Sjoberg DD, Whiting K, Curry M, Lavery JA, Larmarange J. Reproducible summary tables with the gtsummary package. The R Journal 2021;13:570–80. https://doi.org/10.32614/RJ-2021-053.
survey	Lumley T (2023). “survey: analysis of complex survey samples.” R package version 4.2.
survminer	Kassambara A, Kosinski M, Biecek P, Fabian S. survminer: Drawing Survival Curves using ‘ggplot2’. 2021; published online March 9. https://cran.r-project.org/web/packages/survminer/index.html (accessed June 28, 2023).
survival	Therneau TM. A Package for Survival Analysis in R. R package version 3.5-5. 2023; published online March 12. https://cran.r-project.org/web/packages/survival/index.html (accessed June 28, 2023).
weight	Pasek J, Tahk wsafA, Culter scmfRAcbG, Schwemmle. M (2021). _weights: Weighting and Weighted Statistics_. R package version 1.0.4, https://CRAN.R-project.org/package=weights

Supplementary Methods 3: Reference of R packages used for analysis

Supplementary Methods 4: Statistical Analysis

Descriptive Statistics. Within the CHAIN cohort, strata were summarized using mean, median, SD, and IQR for continuous data, and frequency and percent for categorical data. Wilcoxon signed-rank test or Kruskal-Wallis rank-sum test assessed associations in continuous variables between groups, while chi-square tests were used for categorical variables. Rao & Scott's second-order correction was utilized for weighted assessment of categorical data. Paired comparisons were performed for individual-level changes. Odds ratios estimated effect sizes for predictor variables where appropriate. Log2 and Log2+1 transformation managed heteroscedasticity in LPS and proteomics data. Visualizations utilized violin, bar and scatter plots for group comparisons. The 'survey' package was utilized for inverse proportionally weighted summaries.

Justification of 90-day mortality. The impact of plasma LPS on the immune system varies between acute and chronic exposure. Assessing 90-day mortality captures both acute and prolonged LPS effects, enhancing our understanding of its role in survival. Since the CHAIN NCC includes inpatient deaths beyond 30 days, 90-day mortality was useful for evaluating LPS-associated chronic inflammation, immune dysregulation, and immune modulation over time. Additionally, with a subsequent post-discharge mortality peak within the CHAIN NCC around 150 days, this timeframe facilitates the analysis of complex mortality trends without the confounding effects of events beyond 90 days. Studies of cardiac fibrosis in animal models have shown elevated mortality following moderate LPS exposure for 60 to 90 days[1], and there were no differences in sensitivity or specificity in predictive value of plasma LPS in survival outcomes at 30-day, 60-day or 90-day time points (*Supplementary Method 6*) Additionally, with a subsequent post-discharge mortality peak within the CHAIN NCC around 150 days, this timeframe facilitates the analysis of complex mortality trends without the confounding effects of events beyond 90 days.

Cut point of plasma LPS. The 'surv_cutpoint' function from the 'survminer' R package was utilized to derive optimal unweighted cut-off points for continuous variables. This function uses maximally selected log-rank statistics (*Maxstat* method), which identifies the most statistically significant cut-off point by maximizing the difference in survival between two groups based on plasma LPS concentration. The cut-point is clinically useful for identifying thresholds that stratify patients into high and low plasma LPS groups, based on the greatest separation of survival outcomes. Briefly, the *Maxstat* method evaluates numerous candidate cut-off points for plasma LPS and selects the optimal threshold (11.58 E.U./ml in this case) by assessing the maximum value of the log-rank statistic for each candidate cut-off. The selected cut-point was validated through Kaplan-Meier survival curve visualization, which demonstrated significant differences in survival between patients with high vs. low plasma LPS concentrations (*Supplementary Figure 1A*). Additionally, the *Maxstat* method employs bootstrapping to assess the robustness of the selected cut-point and to provide confidence intervals for the cut-off. This resampling approach ensures the reliability of the identified threshold in predicting survival outcomes.

Linear Regression. For predictive analysis of continuous dependent and independent variables, linear regression models were employed. The 'stats' package in R was used to generate linear models. Where appropriate, the correlation coefficient (R) or coefficient of determination (R^2) value were displayed on plots to describe the fit of the regression line and the actual data.

Correlation Models. For proteomic and clinical phenotype data, the strength and direction of correlations were assessed through the inversely proportionally weighted Pearson correlation coefficient method using the 'weights' package. The 'scale' function was used for standardization of predictor variables ensuring comparability of scales. Visualization of correlation matrices was achieved through the corrplot or ggcorrplot packages. Where computationally possible, the 'bootse = TRUE' and 'bootp = TRUE' arguments were used to allow for more accurate estimates through bootstrapping. Significant correlations were specified as $p<0.05$ and $r\geq0.1$ | $r\leq-0.1$. The Benjamini-Hochberg (BH) correction was applied to the p-values from the SomaScan proteomics data to control the False Discovery Rate (FDR), accounting for the high dimensionality and biological complexity of the dataset. This approach balanced sensitivity and specificity which allowed for robust assessment of correlations of proteomics measurements that are inherently variable. Strong, moderate and weak correlations were defined as $R>0.5$, $0.3<R>0.05$ and $R<0.03$,

respectively where Holm-adjusted p-value was also < 0.05. Where hierarchical clustering of correlation matrix was performed, the 'hclust' method was employed.

Adjustment for multiple comparisons. Whenever p-values were generated for analysis, the Holm–Bonferroni method was employed to adjust for multiple hypothesis testing. Holm–Bonferroni adjustment controlled for Type I errors by multiplying unadjusted p-values by the quantity of statistical tests performed. All statistical tests were two-tailed unless otherwise specified.

Mortality status	Anthropometric classification	CHAIN NCC Weights	CHAIN NCC (N)	LPS Analysis (N)	Inverse probability	Final Weights
Additional deaths	SAM (1)	0.39	174	101	1.72	0.67
Additional deaths	MAM (2)	0.4	41	20	2.05	0.82
Additional deaths	Normal (3)	1	28	12	2.33	2.33
Alive within 24% sub cohort	SAM (1)	1.63	231	165	1.40	2.28
Alive within 24% sub cohort	MAM (2)	1.67	168	111	1.51	2.53
Alive within 24% sub cohort	Normal (3)	4.17	258	163	1.58	6.60
Died within 24% sub cohort	SAM (1)	0.39	76	48	1.58	0.62
Died within 24% sub cohort	MAM (2)	0.4	21	10	2.10	0.84
Died within 24% sub cohort	Normal (3)	1	11	8	1.38	1.38
**Total**			**1008**	**638**		

Supplementary Methods 5: Inverse proportional weighting calculations

The CHAIN nested case-cohort (NCC) study sampled participants from the CHAIN cohort based on mortality and anthropometric classification. "CHAIN NCC Weights" were calculated based on the proportion of children sampled into the NCC from each stratum defined by survival status (survived or died) and anthropometric classification (Normal, MAM, or SAM) in the original CHAIN cohort (n = 3,101). The weights were computed using the formula: CHAIN NCC Weight = 1 / (n_NCC / n_COHORT). See CHAIN nested case-cohort study protocol[2] for details on CHAIN NCC selection. "Inverse probability" was calculated based on the frequency of each group within the LPS analyses and computed using the formula: Inverse probability = 1/ (n_LPS_Analysis/ n_NCC). "Final Weights" were calculated as the product of the Inverse probability and CHAIN NCC Weights. SAM = Severe Acute Malnutrition (MUAC <11.5 cm for children aged ≥6 months, MUAC <11 cm for children aged <6 months, or the presence of bilateral pitting oedema), MAM = Moderate Acute Malnutrition (MUAC 11.5 to <12.5 cm for children aged ≥6 months, or MUAC 11 to <12 cm for children aged <6 months), Normal = No Malnutrition (MUAC ≥12.5 cm for children aged ≥6 months, or MUAC ≥12 cm for children aged <6 months).

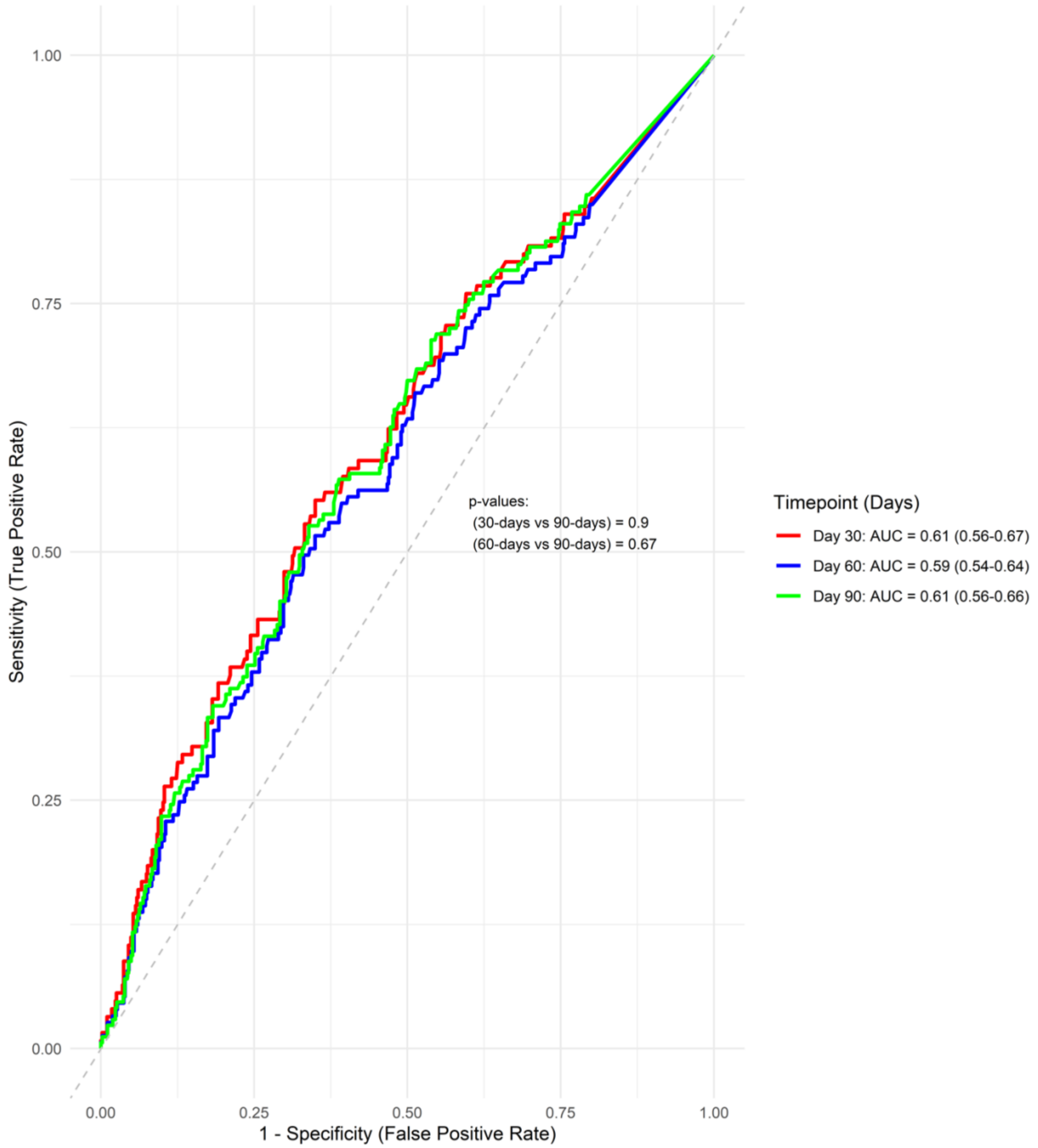

Supplementary Method 6: Time-dependent ROC curves for plasma LPS concentration in predicting survival outcomes.

The ROC curves illustrate the predictive ability of unadjusted plasma LPS concentration for survival outcomes at 30, 60, and 90 days in CHAIN admission children (n = 638). The Area Under the Curve (AUC) values with 95% confidence intervals are displayed in the legend for each time point. The diagonal dashed line represents the reference line for no discrimination (AUC = 0.5). Statistical comparisons of AUC values between time points were performed using two-tailed Z-tests to assess differences between 30- or 60-day versus 90-day AUCs. Colored lines represent 30- (red), 60- (blue), and 90-day (green) time points.

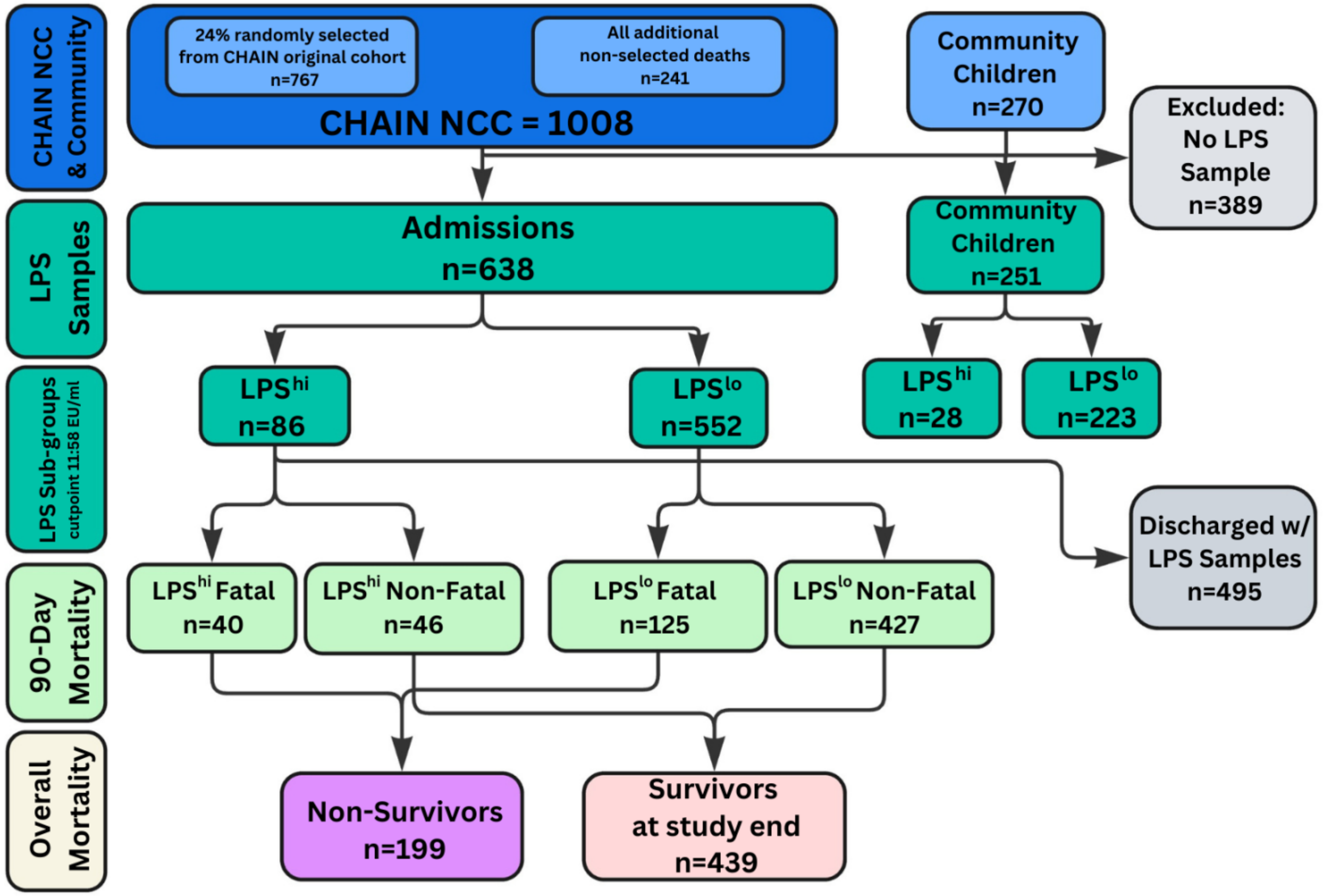

Supplementary Methods 6: CHAIN LPS Analysis Flowchart

Reference	Study Objective	Cohort Description	LPS-associated finding(s)	LPS and mortality finding
**Mwape *et al.*, 2017** **"Immunogenicity of Rotavirus Vaccine (RotarixTM) in Infants with Environmental Enteric Dysfunction."[3]**	Assessed the relationships between EED biomarkers and rotavirus seroconversion after immunization with Rotarix.<br><br>Study assessed LPS signaling biomarkers soluble CD14(sCD14) and Endotoxin Core IgG (EndoCab) ELISA assessment.<br><br>Authors suggested sCD14 and EndoCab are biomarkers of gut leakage.	Retrospective cohort of 142 Zambian infants.	• No evidence that levels of sCD14 or EndoCab are associated with seroconversion status.	No findings specific to LPS-associated mortality.
**Amadi *et al.*, 2017** **"Impaired Barrier Function and Autoantibody Generation in Malnutrition Enteropathy in Zambia."[4]**	Assessed gut structure and function in children with Severe Acute Malnutrition (SAM) and persistent diarrhoea and adult comparison group.<br><br>Study directly assessed plasma LPS and LPS signaling molecules, sCD14 and LPS Binding protein (LBP).<br><br>Authors suggest that LPS is a biomarker of bacterial translocation.	Cross-sectional analysis of 34 children with SAM and persistent diarrhoea in a population from poor communities in Zambia, where stunting is prevalent. 61 adult controls were also investigated. Potential impact of HIV status was also assessed.	• Plasma LPS levels were higher in samples from participants with bacterial DNA detected by 16S RNA compared to those with no bacterial DNA detected.<br>• Plasma LPS was higher in adults and malnourished children compared to healthy children.<br>• Serum LBP was elevated in malnourished children compared to comparison groups.<br>• IGF-1 was inversely correlated with plasma LPS.<br>• Plasma LPS was inversely correlated with GLP-2 and this association was stronger in HIV-negative adults.<br>• TF3 immunoreactivity in duodenal aspirates was associated with reduced plasma LPS.<br>• HIV status was not associated with increased plasma LPS.	No findings specific to LPS-associated mortality.
**Kelly *et al.*, 2010** **"Gastric and Intestinal Barrier Impairment in Tropical Enteropathy and HIV."[5]**	Assessed impact of micronutrient supplementation on gut barrier function.<br><br>Study assessed serum LPS, anti-LPS IgG and anti-LPS IgM.<br><br>Authors used serum LPS, and other biomarkers, as markers of intestinal permeability.	Observational sub-studies nested in a RCT of daily supplementation in children in Zambia. 87 participants were enrolled in the sub-study of mucosal permeability.	• Serum LPS levels were not significantly different among supplementation or HIV status groups.<br>• Anti-LPS IgM was reduced in micronutrient recipients.<br>• No correlation between serum LPS and anti-LPS antibodies levels.<br>• Log-transformed Xylose recovery was negatively associated with log-transformed anti-LPS IgG<br>• Log-transformed TNFRp55 was associated with anti-LPS IgG and anti-LPS IgM.<br>• Authors suggested that background EED may facilitate translocation by other mechanisms than paracellular permeability.	No findings specific to LPS-associated mortality.

Supplementary Methods 7: Findings from studies on LPS, Environmental Enteric Dysfunction and Mortality

This table summarizes the LPS-associated and LPS-mortality-associated findings from previous studies in Environmental Enteric Dysfunction (EED) settings. Three relevant studies were identified through a PubMed search on March 14, 2025, using the following terms: ("EED" OR "Environmental Enteropathy" OR "Environmental Enteric Dysfunction" OR "Environmental Enteric Disease" OR "Tropical Enteropathy" OR "Tropical Enteric Dysfunction") AND ("lipopolysaccharide" OR "LPS" OR "Endotoxin") AND ("mortality" OR "survival" OR "death") AND ("human" OR "children" OR "infant").

Characteristic	Admission, N = 1,894[a]	Discharge, N = 1,635[a]	Community children, N = 251[a]	p-value[b]
Plasma LPS				0.003
Mean (SD)	4.4 (5.3)	3.2 (4.1)	4.5 (5.3)	
Median (Q1, Q3)	2.5 (0.5, 6.0)	2.0 (0.3, 4.2)	2.5 (0.5, 5.9)	
Minimum	0.0	0.0	0.0	
Maximum	29.5	26.5	26.6	
Total N (unweighted)	638	495	251	

[a] N = inverse proportionally weighted total within CHAIN cohort (n=3101)

[b] Kruskal-Wallis rank-sum test for complex survey samples

Supplementary Table 1: Plasma LPS is different amongst cohorts

There were significant differences in plasma LPS concentration among children at admission (n = 638), discharge (n = 495), and in the community (n = 251) (p = 0.003). A two-tailed Kruskal–Wallis rank-sum test was used for comparisons, which yields a single global p-value and does not require multiple-testing correction. SD = standard deviation, Q1 = 25th percentile, Q3 = 75th percentile, N = number of participants in each cohort. E.U./ml = endotoxin units per ml. For admission and community children, n values refer to independent participants, whereas discharged participants represent the subset of admission children discharged from hospital; no technical replicates were used.

Characteristic	Admission<br>*N = 1,894*[a]	Discharge<br>*N = 1,635*[a]	p-value[b]
**Plasma LPS *(E.U./ml)***	2.5 (0.5, 6.0)	2.0 (0.3, 4.2)	0.002
**Anthropometric classification**			0.9
Acute A (SAM)	474 (25%)	415 (25%)	
Acute B (MAM)	306 (16%)	280 (17%)	
Acute C (Normal)	1,115 (59%)	941 (58%)	
**Age group**			0.8
<6 months	361 (19%)	283 (17%)	
6-11 months	690 (36%)	597 (37%)	
12 months & above	844 (45%)	755 (46%)	
**Admission age *(months)***	10.7 (6.6, 15.7)	11.1 (6.9, 15.9)	0.6
**Sex**			>0.9
Female	742 (39%)	648 (40%)	
Male	1,152 (61%)	988 (60%)	

[a] Median (IQR); n (%); N = inverse proportionally weighted total within CHAIN cohort (n=3101)

[b] Wilcoxon rank-sum test for complex survey samples; chi-squared test with Rao & Scott's second-order correction

Supplementary Table 2: Demographic comparisons between admission and discharge cohort
There were no significant differences in key demographic factors (anthropometric classification, age group, age at admission, and sex) between admission (n=638) and discharge (n=495) cohorts. Unpaired, two-tailed Wilcoxon rank-sum tests were used for comparisons of continuous variables, while chi-square tests were used for categorical variables. n values refer to independent participants, where discharge participants represent the subset of admission children discharged from hospital; no technical replicates were used. SAM = severe acute malnutrition, MAM = moderate acute malnutrition, E.U./ml = endotoxin units per ml.

Characteristic[a,b]	Admission,<br>N = 1,211[a,b]	Discharge,<br>N = 1,211[a,b]
Plasma LPS		
Mean (SD)	3.9 (4.9)	3.0 (3.8)
Median (IQR)	2.2 (0.2, 5.5)	1.8 (0.1, 4.1)
Minimum	0.0	0.0
Maximum	28.5	26.5
Total N (unweighted)	378	378

[a] N = inverse proportionally weighted total within CHAIN cohort

[b] P-value (paired weighted Wilcoxon test): 0.14

Supplementary Table 3: Plasma LPS concentration is similar in paired comparison of hospital survivors
There were no significant differences in admission and discharge plasma LPS concentration in hospital survivors (n=378). Paired, two-tailed Wilcoxon rank-sum test was used for comparison. n values refer to independent participants; no technical replicates were used. SD = standard deviation, N = number of participants in cohort. E.U./ml = endotoxin units per ml.

Tertile	Mean Plasma LPS[a]	Total (N)	Total died	% Mortality
All Admissions				
1	0.28	632	27	4.25
2	2.71	632	38	5.94
3	10.24	630	68	10.75
Non-wasted *(MUAC > 11.5 cm)*				
1	0.33	489	12	2.44
2	2.63	486	13	2.61
3	9.53	483	32	6.64
Wasted *(MUAC ≤ 11.5 cm)*				
1	0.14	146	14	9.77
2	3.14	146	30	20.39
3	12.67	144	31	21.83

[a] Measured in E.U./ml
[b] N = inverse proportionally weighted total within CHAIN cohort (n=3101)
`% Mortality' was calculated using unrounded totals

Supplementary Table 4: Plasma LPS concentration is associated with mortality
Children presenting with elevated LPS concentration at hospitalization have enhanced risk of 90-day mortality. 'Total' = inverse proportional weighted total number of children in each group. 'Total died' = inverse proportional weighted total number of children from each group who died within 90 days of admission. '% Mortality' = percentage of children who died within 90-days of admission from that group. E.U./ml = endotoxin units per ml.

Characteristic	Tertile 1, N = 632[b]	Tertile 3, N = 630[b]	p-value[c]
90-day Mortality (*All Admissions*)	27 (4.2%)	68 (11%)	<0.001
**Characteristic**	**Tertile 1,** N = 489[b]	**Tertile 3,** N = 483[b]	**p-value**[c]
90-day Mortality (Non-w*asted*)	12 (2.4%)	32 (6.6%)	0.013
**Characteristic**	**Tertile 1,** N = 146[b]	**Tertile 3,** N = 144[b]	**p-value**[c]
90-day Mortality (*Wasted*)	14 (9.8%)	31 (22%)	0.003

[b] n (%); N = inverse proportionally weighted total within CHAIN cohort (n=3101)
[c] chi-squared test with Rao & Scott's second-order correction

Supplementary Table 5: Mortality is significantly increased in children who presented with elevated LPS
Children presenting with elevated LPS concentration at hospitalization had significantly elevated 90-day mortality. Two-tailed chi-squared tests were used to compare 90-day mortality between children in tertile 1 (n=27) vs. tertile 3 (n=68). n values refer to independent participants; no technical replicates were used. 'N' = inverse proportional weighted total number of children in each tertile. '90-day Mortality' = inverse proportional weighted total number of children from each tertile who died within 90 days of admission.

Characteristic	90-day death<br>*N = 132*	Post 90-day death<br>*N = 29*	Alive at study end<br>*N = 1,733*	p-value[a]
**Plasma LPS *(E.U./ml)***				<0.001
Mean (SD)	7.0 (6.7)	6.2 (7.1)	4.2 (5.0)	
Median (IQR)	*4.8 (1.7, 11.6)*	*3.8 (0.9, 7.7)*	*2.3 (0.4, 5.8)*	
Minimum	0.0	0.0	0.0	
Maximum	29.5	27.3	28.5	
Total n (unweighted)	165	34	439	

N = inverse proportionally weighted total within CHAIN cohort (n=3101)

[a] Kruskal-Wallis rank-sum test for complex survey samples

Supplementary Table 6: Plasma LPS concentration is associated with mortality status
Patients who died within 90-days of hospitalization ('90-day death') (n=165) present with elevated plasma LPS compared to those who died after 90-days of hospitalization ('post-90-day death') (n=34) or who were alive at study end ('Alive') (n=439). A two-tailed Kruskal–Wallis rank-sum test was used for comparisons, which yields a single global p-value and does not require multiple-testing correction. n values refer to independent participants; no technical replicates were used. n = number of children in each cohort (unweighted), N = inverse proportionally weighted numbers, SD = standard deviation E.U./ml = endotoxin units per ml

**Characteristic**	**90-day death** *N = 132*[a]	**Post 90-day death** *N = 29*[a]	**Alive at study end** *N = 1,733*[a]	**p-value**[b]
**Plasma LPS *(E.U./ml)***	4.8 (1.7, 11.6)	3.8 (0.9, 7.7)	2.3 (0.4, 5.8)	<0.001
**Anthropometric classification**				<0.001
Severe Acute Malnutrition (SAM)	82 (62%)	15 (52%)	376 (22%)	
Moderate Acute Malnutrition (MAM)	19 (14%)	6 (20%)	281 (16%)	
Normal	31 (23%)	8 (29%)	1,076 (62%)	
**Age group**				0.4
<6 months	29 (22%)	7 (24%)	325 (19%)	
6-11 months	54 (41%)	12 (42%)	624 (36%)	
12 months & above	49 (37%)	10 (34%)	785 (45%)	
**Admission age (months)**	9.6 (6.4, 15.6)	9.3 (5.9, 14.0)	10.9 (6.7, 15.8)	0.3
**Sex**				0.003
Female	66 (50%)	19 (64%)	658 (38%)	
Male	66 (50%)	10 (36%)	1,075 (62%)	

[a] Median (IQR); n (%); N = inverse proportionally weighted total within CHAIN cohort (n=3101)

[b] Kruskal-Wallis rank-sum test for complex survey samples; chi-squared test with Rao & Scott's second-order correction

Supplementary Table 7: Demographic summary of mortality cohorts
There are significant differences in plasma LPS concentration (p < 0.001), anthropometric classification (p < 0.001) and sex frequency (p = 0.003) among '90-day death' (n=165) 'Post 90-day death' (n=34) and 'Alive' (n=439) cohorts. There is no difference in age between mortality cohorts. A two-tailed Kruskal–Wallis rank-sum test was used for comparisons of numerical variables, which yields a single global p-value and does not require multiple-testing correction. Two-tailed chi-squared tests were used for categorical variables. n values refer to independent participants; no technical replicates were used. SD = standard deviation, SAM = severe acute malnutrition, MAM = moderately acute malnutrition, E.U./ml = endotoxin units per ml.

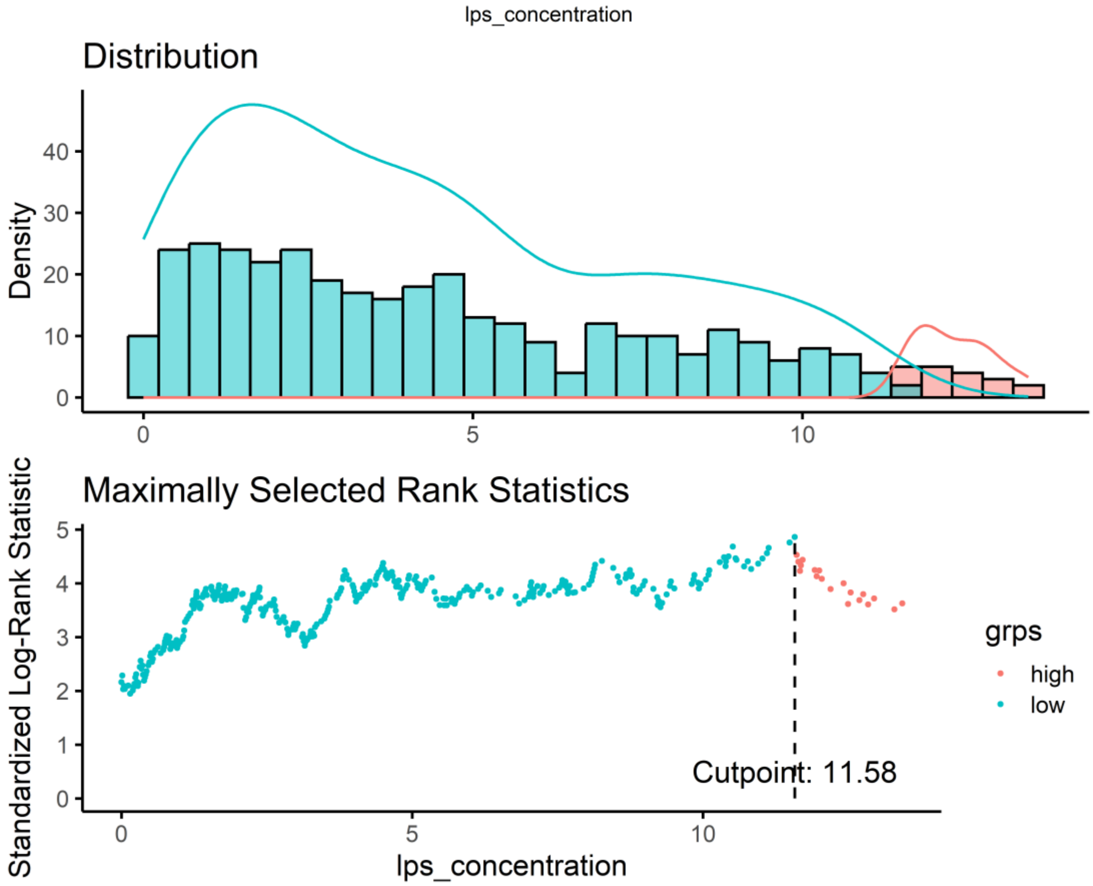

Supplementary Figure 1A: LPS cutoff analysis of Log-Rank Statistic for 90-day survival

The optimal cut point of LPS based on maximally selected rank statistics of 90-day survival outcome is 11.58 E.U./ml for the CHAIN LPS study (n=638). The maximally selected rank statistics stratifies the cohort into two groups (high vs. low) and tests all possible cut points using two-tailed tests. The standardized statistic of the cut point that most significantly separates the groups is selected, with an adjustment for multiple testing inherent in the maximally selected rank statistic procedure. This cut point is then used to define high and low expression within the cohort. Color bars, lines, and dots denote the high (red) or low (blue) group.

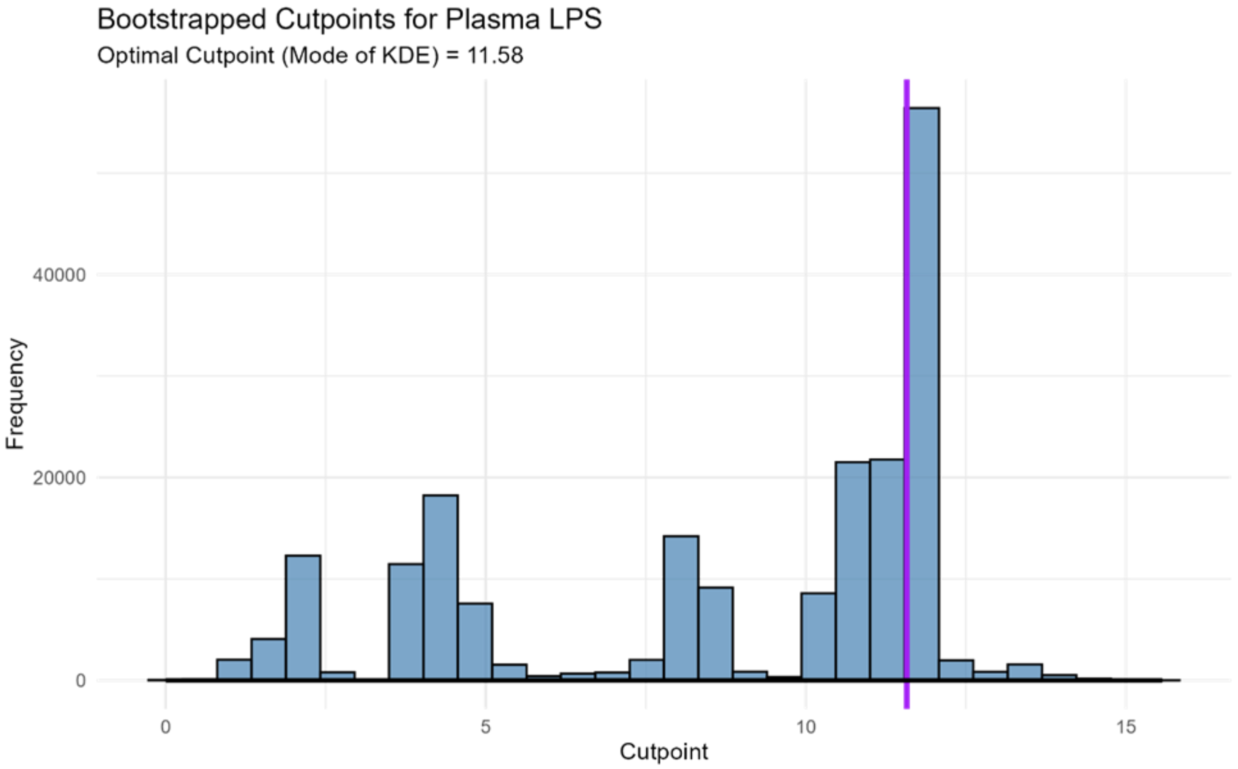

Supplementary Figure 1B: Bootstrap-based analysis of optimal LPS cut point for 90-day survival

Maximally selected rank statistics with 200,000 bootstrap resamples were used to determine the optimal cut point of plasma LPS concentration for predicting 90-day mortality. This method iteratively evaluates all possible cut points and identifies the threshold that most significantly separates survival curves based on the standardized log-rank statistic (Supplementary Figure 1A). The bootstrap distribution of optimal cut points identified 4 clusters and specified 11.58 E.U./ml (highlighted with purple vertical line) as the most frequently selected cut point across resamples. This threshold was used to stratify children into high ('hi') and low ('lo') LPS expression groups for survival analysis in the CHAIN LPS cohort.

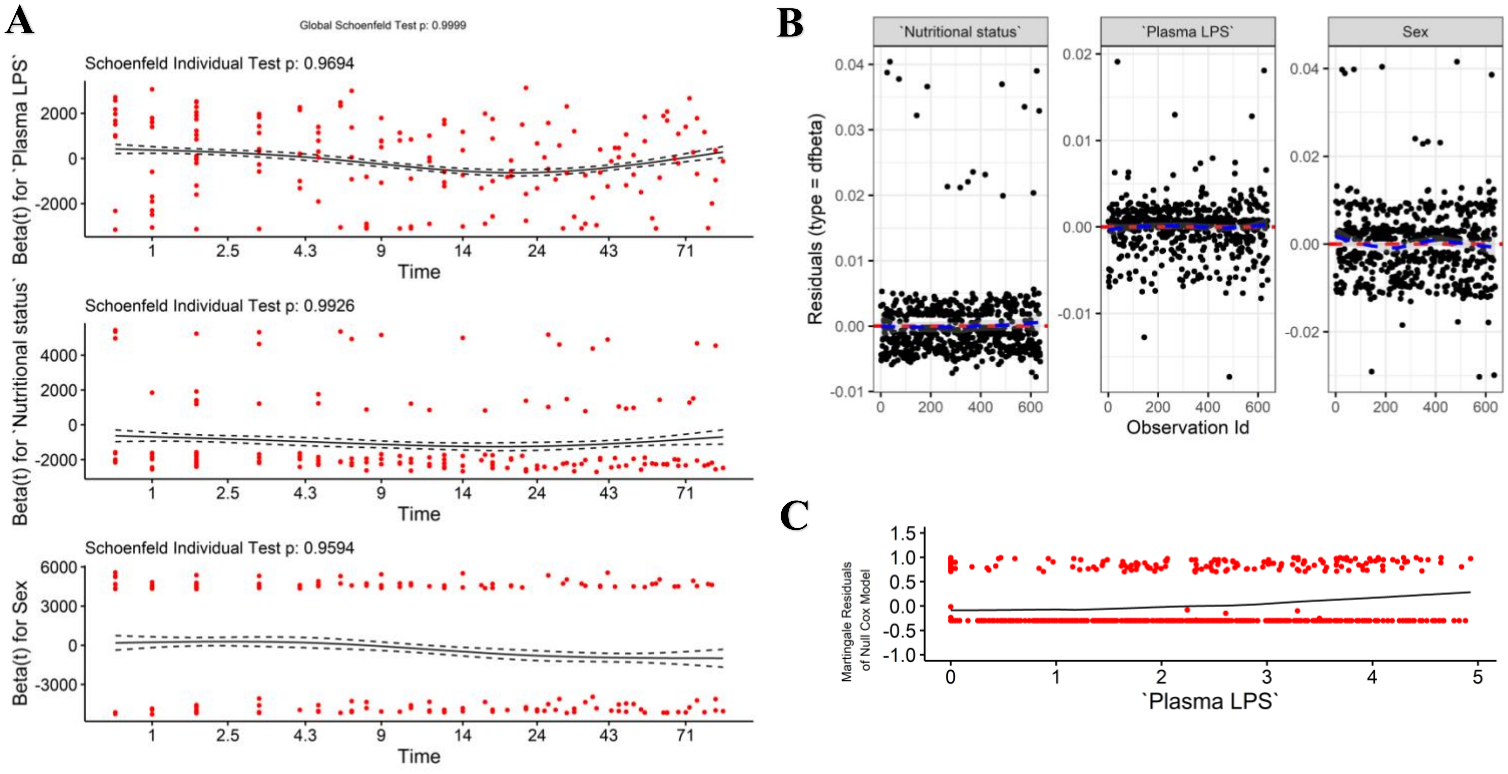

Supplementary Figure 2: Proportional Hazard assumption is satisfied for CoxPH model

Plots show (A) scaled Schoenfeld individual test and (B) dfbeta values for testing influential for all variables included for CoxPH model of 90-day mortality with weighted plasma LPS concentration, with nutritional status and sex as covariates. (C) Martingale residuals for the continuous variable, plasma LPS, shows non-linearity.

Clinical Feature	Coefficient
Cold Peripheries level	-0.40
Plasma LPS concentration	0.28
Dehydration	0.26
Admission MUAC groups	-0.24
Site continent	0.23
Capillary Refill	0.21
Drinking/breast feeding status	-0.18
Fecal Calprotectin	0.18
Typical enteropathogenic E. coli, bfpA positive	-0.14
Normal mouth	-0.13
Associated with Azithromycin resistance, plasmid-borne	-0.13
Enrolment MUAC	-0.12
Feeding status	0.11
Skin recovers after pinch	0.11
Glucose	0.11
Rurality	-0.09
Perineum rash	0.09
Traditional medicine use	0.07
Sunken eyes	0.07
Enrolment Length-for-age z-score	-0.07
Giardia lamblia	0.06
Oedema	0.06
Vitamin supplementation	-0.05
Dermatitis	0.05
Enrolment Weight-for-age z-score	-0.05

Supplementary Table 8: Selection of clinical phenotype after elastic net regularization with 90-day mortality
Top 25 clinical features associated with 90-day survival were assessed for admission children (n=638) using Elastic net regularization regression. Analysis was performed on split training (70%) and testing (30%) datasets, with both models utilizing the "cox" family. Statistical tests within the Cox models were two-tailed. Variables were excluded to account for multicollinearity, missingness over 50%, and zero variance. The best alpha (0.4) and lambda (0.052) determined from the training regression were applied to the testing model. Variable trace plots for Elastic net regression are presented in Supplementary Figure 4.

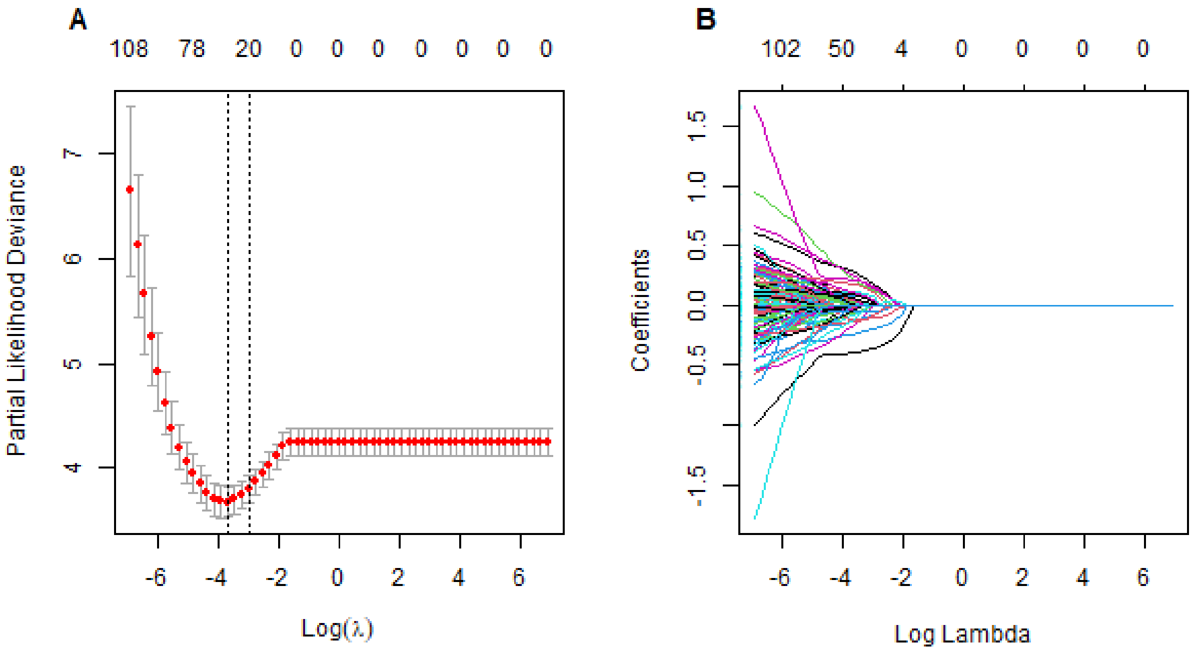

Supplementary Figure 3: Variable trace plots of Elastic Net Regression analysis of clinical features
(Left) Cross-validated partial likelihood deviances. Vertical, solid lines represent partial likelihood deviance ± standard error (SE). The leftmost vertical, dotted line represents the ideal lambda with minimal deviance while the rightmost vertical, dotted line represents the 1-SE criteria. The value for lambda chosen was $\lambda = 0.052$, which best minimized Mean Squared Error. (Right) Elastic net coefficient profiles of over 200 clinical features of the CHAIN admissions cohort.

	**Admission** [a] *Mean (SD)*	**Admission** [a] *Median (Q1, Q3)*	**Community** [a] *Mean (SD)*	**Community** [a] *Median (Q1, Q3)*	**p-value** [e]	**High-income reference for elevated levels**
**Fecal Biomarkers**						
Alpha-1-antitrypsin (µg/ml) (*non-acute diarrhoea*)[b]	449 (557)	246 (101, 470)	341 (373)	203 (100, 412)	0.30	270 µg/ml[6]
Myeloperoxidase (ηg/ml)[c]	4106 (4585)	2228 (868, 6263)	3709 (3553)	2255 (1053, 5411)	0.70	<2000 ηg/ml[7]
Calprotectin (µg/ml)[d] *(≥12 months)*	699 (1,086)	251 (91, 912)	503 (1,193)	188 (105, 514)	0.13	122 µg/ml[8]
**Stunting**						
HAZ [c]	-1.62 (1.68)	-1.53 (-2.55, -0.70)	-1.37 (1.36)	-1.41 (-2.23, -0.75)	0.08	0[f]
Percent HAZ < -2 [c]	38%		31%		0.05	

[a] N = inverse proportionally weighted total within CHAIN cohort (N =3101)
[b] *Unweighted n = 271 (admission), 129 (community children); Weighted N = 844 (admission), 129 (community children)*
[c] *Unweighted n = 638 (admission), 251 (community children); Weighted N = 1894 (admission), 251 (community children)*
[d] *Unweighted n = 233 (admission), 251 (community children); Weighted N = 731 (admission), 251 (community children)*
[e] Design-based Kruskal-Wallis test; Pearson's X^2: Rao & Scott adjustment based on comparison of CHAIN Admission and Community children
[f] World Health Organization reference

Supplementary Table 9: Fecal EED biomarker expression and growth stunting in CHAIN population
Fecal EED biomarkers and stunting are elevated in CHAIN admissions (n=638) and community children (n=251) compared to references from high-income cohorts. CHAIN admission and community children have similar (p ≥ 0.05) expressions of fecal EED biomarkers and frequency of stunting. Unpaired, two-tailed Wilcoxon rank-sum tests were used for comparisons of continuous variables, while chi-square tests were used for categorical variables. n values refer to independent participants; no technical replicates were used. Summary statistics are inverse proportionally weighted to account for selection biases. HAZ = Length-for-age Z-score or Height-for-age Z-score.

**Country**<br>**(*cohort, n*) (Reference)**	**Age range**	**Cohort Description**	**Alpha-1-antitrypsin**<br>*Mean (SD) μg/ml or μg/g*	**Myeloperoxidase**<br>*Mean (SD) ηg/ml*	**Calprotectin**<br>*Mean (SD) μg/ml or μg/g*	**Length-for-age Z-score (HAZ)** *Mean (SD)*	**% Stunting**<br>*LAZ/HAZ <-2SD*
**CHAIN community**<br>(*n=251*)	Under 5 years	Community-based comparison group from the CHAIN cohort. Children were asymptomatic to serious diseases and/or acute infections.	341 (373)	3,709 (3,553)	576 (1,017)	-1.37 (1.36)	31%[†]
**Peru**<br>MAL-ED cohort (*n=303*)<br>(Colston *et al.,* 2017)	Birth - 5 years	Observation study of 303 infants from a semi-urban community situated in the forested interior of the country. Study exclusion: 1) children with serious illnesses, 2) children whose mother < 16 years of age at birth, 3) children from non-singleton pregnancy. Stool samples were collected in the 2 days before a diarrheal episode. Summary statistics were generated from longitudinal data.	598.3 (651.0)	12,482.8 (12,910.7)		-1.6 (0.9)	
**Multiple countries**<br>MAL-ED cohort<br>*Bangladesh (n=186),*<br>*Brazil (n=99),*<br>*India (n=207),*<br>*Nepal (n=122),*<br>*Peru (n=145),*<br>*South Africa (n=132),*<br>*Tanzania (n=126)*<br>(Richard *et al.,* 2019)	Birth - 5 years	The MAL-ED observational cohort of 1017 children were conducted at multiple sites. Inclusion criteria included birth weight >1500 g, no serious illnesses, mother at least 16 years of age at infant's birth. Samples collected during diarrhoea episodes were excluded from the analysis.	Bangladesh: 400 (100)<br>India: 400 (100)<br>Nepal: 400 (100)<br>Brazil: 300 (100)<br>Peru: 400 (200)<br>South Africa: 200 (100)<br>Tanzania: 300 (200)	Bangladesh: 4380 (1834)<br>India: 7866 (3959)<br>Nepal: 4166 (1880)<br>Brazil: 3008 (2394)<br>Peru: 7950 (3878)<br>South Africa: 4614 (1750)<br>Tanzania: 5511 (2520)<br>*(Measured in: ng/mol)*		**At Enrollment:**<br>Bangladesh: −1.0 (1.0)<br>India: −1.0 (1.1)<br>Nepal: −0.7 (1.0)<br>Brazil: −0.8 (1.2)<br>Peru: −1.0 (0.9)<br>South Africa: −0.8 (1.0)<br>Tanzania: −1.0 (1.2)<br><br>**At 5 years old:**<br>Bangladesh: −1.6 (0.9)<br>India: −1.5 (0.9)<br>Nepal: −1.3 (0.9)<br>Brazil: −0.2 (1.0)<br>Peru: −1.3 (0.8)<br>South Africa: −0.9 (1.0)<br>Tanzania: −1.9 (0.9)	
**Multiple countries**<br>Afrobiota project<br>Madagascar (*n=417*),<br>Central African Republic (CAR) (*n=387*)<br>(Vonaesch *et al.,* 2022)	2-5 years old	Cross-sectional study on stunting of 804 children aged 2–5 years from Madagascar and Central African Republic. Study did not include children experiencing severe diseases.	Madagascar: 590.7 (551.0)<br>CAR: 396.3 (318.6)		Madagascar: 764.5 (1024.4)<br>CAR: 549.2 (647.4)	Madagascar: -2.0 (1.1)<br>CAR: -1.8 (1.4)	Madagascar: 48%<br>CAR: 44%

**Country**<br>**(*cohort, n*) (Reference)**	**Age range**	**Cohort Description**	**Alpha-1-antitrypsin**<br>*Median [Q1,Q3]* μg/ml or μg/g	**Myeloperoxidase**<br>*Median [Q1,Q3]* ηg/ml	**Calprotectin**<br>*Median[Q1,Q3]* μg/ml or μg/g	**Length-for-age Z-score (HAZ)** *Median [Q1,Q3]*	**% Stunting**<br>*LAZ/HAZ <-2SD*

**CHAIN community** (*n=251*)	Under 5 years	Community-based comparison group from the CHAIN cohort. Children were asymptomatic to serious diseases and/or acute infections.	203 [100, 412]	2,255 [1,053, 5,411]	254 [124, 692]	-1.41 [-2.23, -0.75]	31%†
**Bangladesh** MAL-ED cohort (*n=246*) (Arndt *et al.*, 2016)	3-24 months	Observational study of 246 children from the Mirpur urban slum. Study exclusion included: 1) children with serious illnesses, 2) children whose mother < 16 years of age at birth, 3) children from non-singleton pregnancy. Participants were followed until 2 years old.	3-21 months: 380 [190, 717.5] *(1,194 samples)*	3-21 months: 3354.9 [1,594.9, 7430.1] *(1,185 samples)*		Birth: -0.99 [−1.68, -0.40]<br>3 months: -1.17[-1.80, -0.47]<br>24 months: -1.99[-2.60, -1.30]	Birth: 16.7%<br>3 months: 15.6%<br>24 months: 49.5
**Bangladesh** GEMS DSS (*n=216*) (George *et al.*, 2015)	6-30 months	Nested cohort study of 216 randomly selected children from rural Bangladeshi households with live chickens in their compound. Participants were followed for 9 months.	260 [160, 510]	3,576.75 [1,969.50, 5,998.00]	402.67 [193.37, 822.30]		9-months follow-up: 34 (n=205)
**Zimbabwe** SHINE trial (*n=1169*) (Mutasa *et al.*, 2021)	0-18 months	Observational study of a subgroup of 1169 infants in rural Zimbabwe. Mothers were uninfected with HIV at enrollment. Children were followed longitudinally.	1 month:350 [170,980]<br>3 months:360 [180,780]<br>6 months:340 [190,640]<br>12 months:300 [160,580]<br>18 months:230 [110,420]	1 month: 5861 [2777,11647]<br>3 months: 8221 [3962,18074]<br>6 months: 7233 [4166,12413]<br>12 months: 3959 [2107,7044]<br>18 months: 2063 [967,4109]		1 month: -0.79 [-1.62, -0.03]<br>3 months: -0.84 [-1.58, -0.07]<br>6 months: -0.87 [-1.61, -0.07]<br>12 months: -1.12 [-1.87, -0.46]<br>18 months: -1.41 [-2.80, -0.74]	
**Multiple countries** Afrobiota project Madagascar (*n=417*), Central African Republic (CAR) (*n=387*) (Vonaesch *et al.*, 2022)	2-5 years old	Cross-sectional study on stunting of 804 children aged 2–5 years from Madagascar and Central African Republic. Study did not include children experiencing severe diseases.	Madagascar: 500 [220, 795]<br>CAR: 330 [130, 573]		Madagascar: 448 [249, 853]<br>CAR: 308 [172, 617]	Madagascar: -1.97 [-2.83, -1.31]<br>CAR: -1.75 [-2.88, -0.77]	Madagascar: 48%<br>CAR: 44%
**India** MANTRA program (*n=221*) (Sinharoy *et al.*, 2021)	Under 5 years	Nested sub-study of 221 community control children within the larger MANTRA program. Matched cohort study of a household-level water and sanitation intervention in rural Odisha, India.	355.2 [207.6,620.8]	812.8 [495.4,1477.2]		-1.73 (1.32) (Mean)	
**Bangladesh** MAL-ED (*n=265*) (Fahim *et al.*, 2018)	Birth - 2 years	Observational study of 265 healthy newborns from an urban community with low socioeconomic status and inadequate sanitation. Study exclusion included: 1) children with serious illnesses, 2) children whose mother < 16 years of age, 3) children from non-singleton pregnancy.	330 [180,620] *(627 samples)*	3895.42 [1,563.76, 8432.82] *(625 samples)*		7 months: −1.22 [−1.87, −0.58]<br>15 months: −1.74 [−2.42, −1.15]<br>24 months: −1.98 [−2.60, −1.30]	7 months: 20.3%<br>15 months: 41.7%<br>24 months: 47.9%

Supplementary Table 10: Comparison of fecal EED biomarkers and stunting in the CHAIN community children and other EED studies

Fecal biomarkers of environmental enteric dysfunction (EED) and stunting were similarly elevated in the community-based comparison group from the CHAIN cohort compared to other published EED cohorts. Summary statistics for fecal EED biomarkers and Length-for-Age Z-score (HAZ) are reported as Mean (SD) (upper panel) or Median (Q1, Q3) (lower panel) , while stunting prevalence is reported as a percentage (%).

Relevant studies were identified through a PubMed search using the following terms on March 14, 2025: ("EED" OR "Environmental Enteropathy" OR "Environmental Enteric Dysfunction" OR "Environmental Enteric Disease" OR "Tropical Enteropathy" OR "Tropical Enteric Dysfunction") AND (("Myeloperoxidase" OR "MPO") AND ("alpha-1 antitrypsin" OR "AAT") OR ("Calprotectin" AND ("alpha-1 antitrypsin" OR "AAT")) OR (("Myeloperoxidase" OR "MPO") AND "Calprotectin")) AND ("HAZ" OR "LAZ" OR "height-for-age" OR "length-for-age" OR "stunting"). Inclusion criteria: Studies were included if they assessed at least ***two*** *of the three key fecal biomarkers (α-1-antitrypsin, myeloperoxidase, calprotectin) alongside stunting. When multiple studies were available from the same cohort and sample population, the study most like the CHAIN cohort in age and population characteristics was selected.*

Characteristics	Admission<br>Weighted N = 1,894[a]<br>Unweighted n = 638	Community children<br>Weighted N = 251[a]<br>Unweighted n = 251	p-value[b]
Plasma LPS *(E.U./ml)(Mean (SD)*	4.40 (5.25)	4.47 (5.34)	>0.9
Demographics			
Age group			0.2
<6 months	361 / 1,894 (19%)	41 / 251 (16%)	
6-11 months	690 / 1,894 (36%)	81 / 251 (32%)	
12 months & above	844 / 1,894 (45%)	129 / 251 (51%)	
Admission age *(Mean (SD))*	11.47 (5.57)	12.48 (6.11)	0.049
Sex			0.047
Female	742 / 1,894 (39%)	118 / 251 (47%)	
Male	1,152 / 1,894 (61%)	133 / 251 (53%)	
Continent			0.032
Africa	1,085 / 1,894 (57%)	165 / 251 (66%)	
Asia	809 / 1,894 (43%)	86 / 251 (34%)	
Comorbidity frequencies			
Malaria	337 / 1,894 (18%)	11 / 251 (4.4%)	<0.001
Sepsis	295 / 1,894 (16%)	0 / 251 (0%)	<0.001
Gastroenteritis	1,142 / 1,872 (61%)	0 / 251 (0%)	<0.001
URTI	98 / 1,894 (5.2%)	0 / 251 (0%)	0.060
LRTI	774 / 1,894 (41%)	0 / 251 (0%)	<0.001
HIV status			0.2
negative	1,844 / 1,894 (97%)	248 / 251 (99%)	
positive	50 / 1,894 (2.6%)	3 / 251 (1.2%)	
TB	3 / 1,701 (0.2%)	0 / 221 (0%)	0.6
Breastfeeding status	1,428 / 1,894 (75%)	202 / 251 (80%)	0.12
Entero-pathogen frequencies			
Viruses	776 / 1,702 (46%)	53 / 222 (24%)	<0.001
Parasites	517 / 1,770 (29%)	56 / 231 (24%)	0.2
Gram-negative Bacteria	1,579 / 1,820 (87%)	206 / 241 (85%)	0.6
***Entero-pathogens Quantification** (Mean (SD))*[c]			
Aeromonas	34.95 (0.50)	34.88 (0.90)	0.4
Campylobacter_pan	32.08 (4.87)	32.72 (3.75)	>0.9
Campylobacter_jejuni_coli	33.19 (4.02)	33.83 (3.06)	0.11
EAEC	28.85 (6.31)	30.31 (5.32)	0.007
tEPEC	33.62 (4.03)	34.24 (2.50)	0.069
aEPEC	33.77 (3.02)	33.92 (2.90)	0.6
ETEC	32.79 (4.69)	33.86 (2.91)	0.008

ST_ETEC	33.51 (4.06)	34.63 (1.71)	<0.001
LT_ETEC	34.44 (2.33)	34.29 (2.34)	0.2
H_pylori	34.96 (0.38)	35.00 (0.02)	0.054
Plesiomonas	34.91 (0.75)	34.94 (0.55)	0.6
Salmonella	34.86 (1.01)	34.97 (0.37)	0.068
Shigella_EIEC	33.66 (4.04)	34.13 (2.67)	0.6
STEC	34.96 (0.36)	34.91 (0.71)	0.4
V_cholerae	34.79 (1.53)	35.00 (0.02)	0.006
CTX_M	27.24 (6.79)	30.62 (4.99)	<0.001
mphA Azithromysin Resistance	23.08 (6.47)	27.36 (5.69)	<0.001
X16s	13.90 (3.28)	15.25 (3.71)	<0.001
C_difficile	34.95 (0.67)	34.94 (0.49)	0.3
Ascaris	35.00 (0.06)	35.00 (0.00)	0.2
Cyclospora	35.00 (0.00)	35.00 (0.02)	0.3
E_bieneusi	34.83 (1.05)	34.85 (0.93)	0.7
E_intestinalis	34.96 (0.52)	34.99 (0.10)	>0.9
Giardia	34.07 (2.81)	34.04 (2.76)	0.8
Isospora	35.00 (0.08)	35.00 (0.00)	0.084
Necator	34.99 (0.17)	35.00 (0.01)	0.9
Cryptosporidium	33.90 (3.46)	34.35 (2.58)	0.015
Adenovirus_40_41	34.22 (3.19)	34.71 (1.59)	0.005
Astrovirus	34.69 (1.73)	34.87 (1.28)	0.11
Norovirus_GI	34.92 (0.74)	34.82 (0.98)	0.2
Norovirus_GII	34.28 (2.54)	34.60 (1.70)	0.4
Rotavirus	33.01 (4.26)	34.92 (0.82)	<0.001
Sapovirus	34.64 (1.65)	34.89 (0.77)	0.029
M_tuberculosis	34.99 (0.12)	35.00 (0.00)	0.2
***Fecal Biomarkers** (Mean (SD))*			
Fecal MPO	4,105.51 (4,584.89)	3,708.52 (3,552.86)	0.7
Fecal Calprotectin	587,752.29 (961,750.19)	575,652.43 (1,016,548.01)	0.029
Fecal Calprotectin (> 12 months)	698,853.84 (1,086,330.11)	502,960.37 (1,192,810.79)	0.13
Fecal AAT	285,739.78 (423,524.75)	341,356.60 (372,631.91)	<0.001
Fecal AAT (non-acute diarrhoea)	448,574.75 (557,006.86)	341,356.60 (372,631.91)	0.30
***Plasma Barrier Function Biomarkers** (Mean (SD)*			
Plasma Diamine Oxidase	4,776.11 (4,226.63)	6,809.30 (3,594.82)	<0.001
Plasma Zonulin	46,479.64 (29,948.42)	33,384.53 (19,464.19)	<0.001
FABP2	3,208.20 (2,915.06)	2,746.65 (1,951.33)	0.3
***Plasma Systemic Inflammatory Biomarkers** (Mean (SD)*			

Plasma MPO	27,618.30 (14,306.43)	16,296.82 (5,131.00)	<0.001
Plasma Calprotectin	1,773.65 (1,065.01)	1,749.45 (3,856.34)	<0.001
Plasma AAT	50,645.94 (15,708.32)	33,395.31 (9,471.53)	<0.001
Plasma SAA1	72,275.72 (72,226.17)	17,974.93 (35,545.88)	<0.001
Plasma SAA4	62,337.92 (29,839.60)	60,201.82 (27,614.05)	0.055
LPS Signal Transduction Markers *(Mean (SD)*			
CD14	32,196.14 (8,332.73)	25,194.53 (5,722.89)	<0.001
CD14.1	1,716.73 (743.00)	1,181.90 (292.50)	<0.001
TLR4	512.05 (1,017.52)	426.84 (163.61)	0.6
LBP	67,277.46 (33,676.12)	36,920.60 (17,629.39)	<0.001

[a]N = inverse proportionally weighted total within CHAIN cohort (n=3101)

E.U./ml = Endotoxin Unit per ml

[b]Design-based Kruskal-Wallis test; Pearson's X^2: Rao & Scott adjustment

[c] *Threshold Cycle (Ct) from quantitative PCR (qPCR) assay*

Supplementary Table 11: Demographic, Entero-pathogen, Fecal Biomarker and Plasma Barrier and Systemic Inflammation Profiles of Hospitalized vs. Community Children

Demographics (age, sex, continent), fecal biomarkers (MPO, calprotectin, AAT), and plasma LPS concentrations are comparable between admission (n=638) and community children (n=251). In contrast, the presence of comorbidities (malaria, sepsis, gastroenteritis, LRTI), barrier protein expression (zonulin, diamine oxidase) and plasma systemic inflammatory (CRP, SAA1, MPO, calprotectin, AAT) and LPS signal transduction biomarkers (CD14, soluble CD14, LPS binding protein) differ significantly between the two cohorts. Both unweighted (n) and inverse proportionally weighted totals (N) are presented. Unpaired, two-tailed Wilcoxon rank-sum tests were used for comparisons of continuous variables, while chi-square tests were used for categorical variables. n values refer to independent participants; no technical replicates were used. Comparisons with significant differences are highlighted in green. The following entero-pathogens were excluded from analysis because all tests in admission or community children were negative or missing: Ancylostoma, E.histolytica, Strongyloides and Trichuris. LPS = lipopolysaccharide, MPO = myeloperoxidase, AAT = Alpha-1-antitrypsin, FABP2 = Intestinal-type fatty acid-binding protein, SAA = Serum Amyloid A, TLR4 = Toll-like Receptor 4, CD14.1 = membrane CD14, CD14 = soluble CD14.

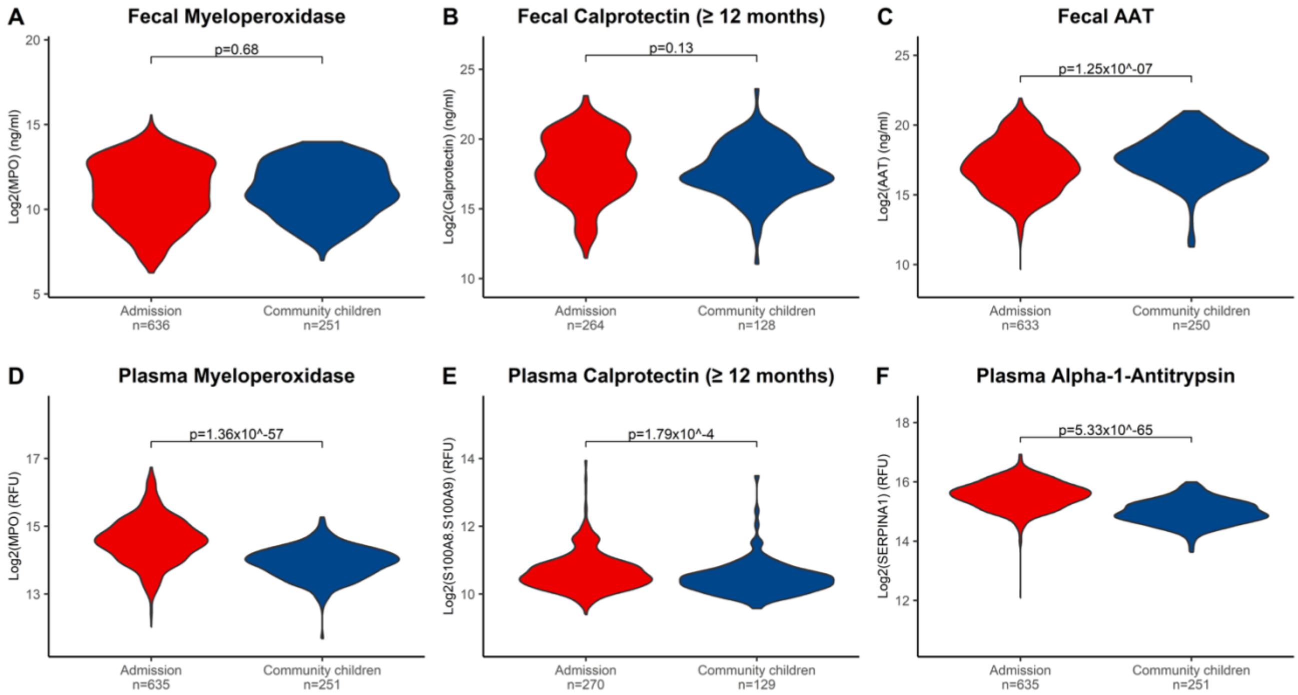

Supplementary Figure 4A-F: Systemic biomarkers of inflammation are significantly elevated in the admission cohort

Expression of biomarkers of intestinal inflammation, (A) myeloperoxidase (MPO) and (B) calprotectin, are not significantly different between admission (n=638) and community children (n=251). (C) Fecal Alpha-1-antitrypsin (AAT) is elevated in community children compared to admissions cohort. Plasma biomarkers of systemic inflammation, (D) MPO, (E) calprotectin and (F) AAT are significantly elevated in the admissions cohort. Comparisons were assessed by two-tailed, unpaired Wilcoxon signed-rank test and were inverse proportionally weighted to account for selection bias. n values refer to independent participants where at least 1 biomarker value was available; no technical replicates were used. Colored violin represents admission and community cohort. The x-axis denotes the cohort while the y-axis represents biomarker expression in units of Log2 transformed μg/ml, ηg/ml or RFU. RFU = Relative fluorescence units.

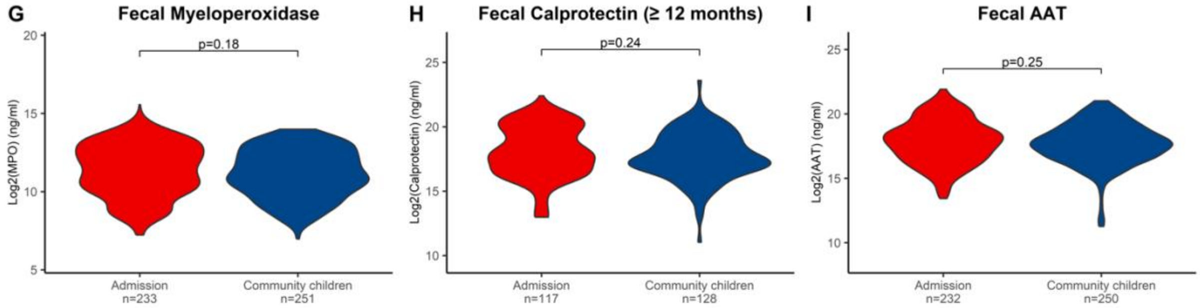

Supplementary Figure 4G-I: Biomarkers of intestinal inflammation and permeability in hospitalised children without acute diarrhoea at admission and community children.

Expression of fecal biomarkers of intestinal inflammation, (G) myeloperoxidase (MPO), (H) calprotectin and (I) Alpha-1-antitrypsin (AAT), are not significantly different between children without acute diarrhoea at admission (n=233) and community children (n=251). (H) Fecal Calprotectin was only assessed for children 12 months or older. Comparisons were assessed by unpaired, two-tailed Wilcoxon signed-rank test and were inverse proportionally weighted to account for selection bias. n values refer to independent participants where at least 1 biomarker value was available; no technical replicates were used. Colored violin represents admission and community cohort. The x-axis denotes hospitalization or community status while the y-axis represents biomarker expression in units of Log2 transformed µg/ml, ηg/ml.

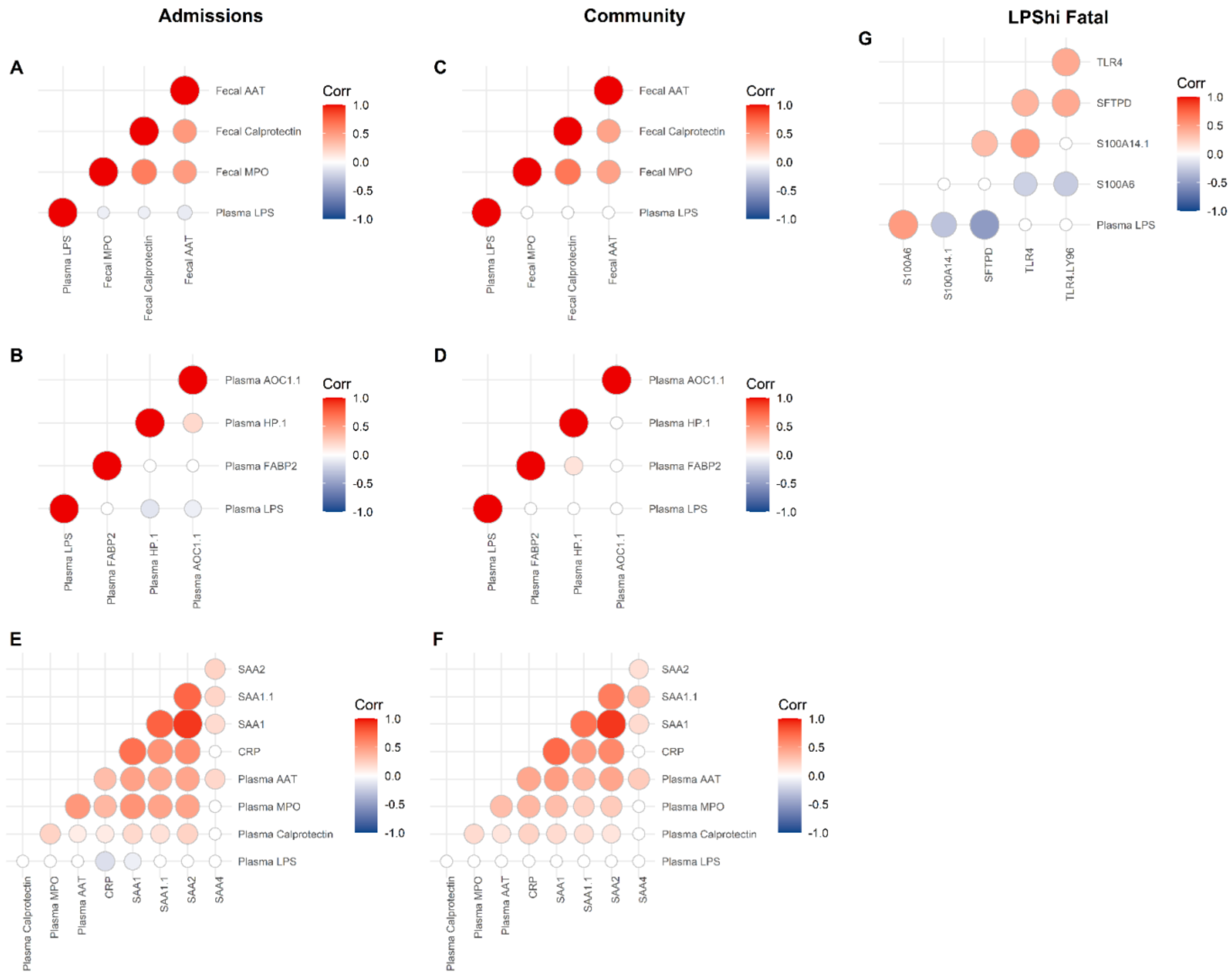

Supplementary Figure 5: Plasma LPS has differential sub-group associations with barrier function biomarkers. *Plasma LPS is weakly correlated with (A) fecal EED and (B) plasma barrier function biomarkers in the admissions cohort (n=638) but not in community children (n=251) (C and D, respectively). Plasma LPS is weakly associated with (E) CRP and SAA1 in the admissions cohort and (F) SAA4 in community children. Plasma LPS is correlated with genes that encode LPS endogenous ligands (G) S100A6 and SFTPD (strongly: R > 0·5) and S100A14.1 (moderately: R>0·3) in the LPS^{hi} F admissions group (n=39). Correlations matrices were inverse proportionally weighted to address selection biases. Only correlations with a two-tailed p-value < 0·05 are shown. Bootstrapping of p-values and standard error was performed to increase statistical rigor. All p-values were Benjamini-Hochberg (BH)-adjusted for false discovery rates. All n values refer to independent children; no technical replicates were used. LPS = lipopolysaccharide, MPO = myeloperoxidase, AAT = Alpha-1-antitrypsin, FABP2 = Intestinal-type fatty acid-binding protein, HP = Haptoglobin, AOC1 = Amine Oxidase Copper-Containing 1, S100A6 = Calcyclin, S100A14.1 = S100 calcium-binding protein A14, SFTPD = Surfactant Protein D, SCARB1 = Scavenger receptor class B member 1, TLR4 = Toll-like Receptor 4, TLR4.LY94 = TLR4-MD2 complex. F = Fatal.*

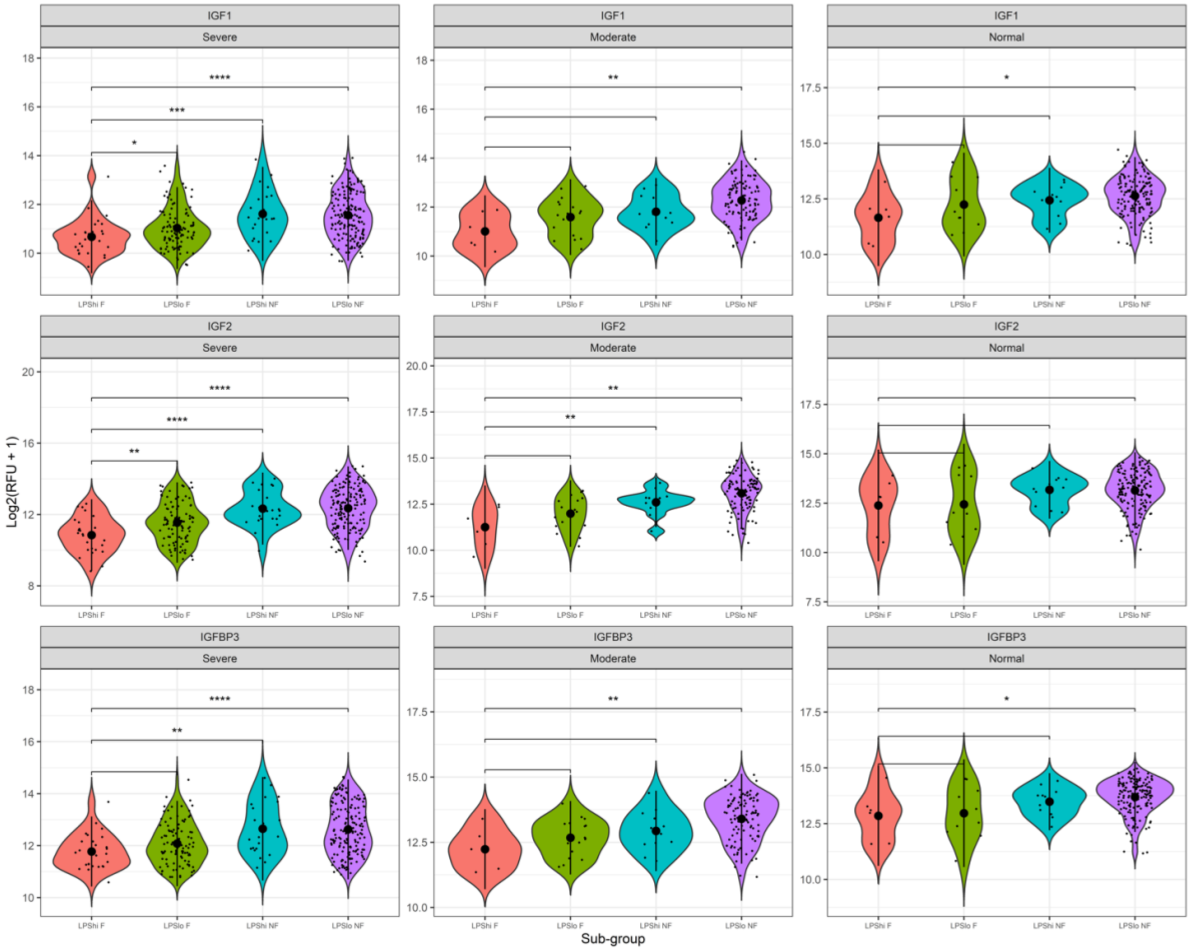

Supplementary Figure 6: Expression of IGF proteins among sub-groups stratified by anthropometric classification

Proteins of Insulin-like Growth Factor nutritional axis were significantly reduced in children from the LPS^{hi} Fatal (n=39) compared to other sub-groups. Comparisons were stratified by anthropometric classification ("Severe" (SAM), "Moderate" (MAM), and "Normal"). All proteins had significantly different expressions ($p < 0{\cdot}05$) as assessed by two-tailed Wilcoxon signed-rank test and were Holm-adjusted for multiple comparisons. Central tendencies are shown as large dots representing mean ± standard deviation (SD), while individual values for each child are indicated by small dots. All n values refer to independent children; no technical replicates were used. The x-axis denotes the LPS sub-groups while the y-axis represents protein expression in units of Log2 transformed (RFU+1). RFU = Relative fluorescence units. F = Fatal, NF = Non-fatal.

	CORRELATION COEFFICIENT	ADJUSTED P-VALUE
PLASMA LPS	1	0
GOLM2	-0.403041241	0.003034158
SELPLG	-0.457752658	0.000358588
ADH5	0.402791084	0.003060736
DNAJB2	0.401941138	0.003152971
TRAF4	-0.409055307	0.002452456
CD7	-0.423650484	0.00143083
NRBP1	0.413019831	0.002125059
KCNG4	0.408410818	0.002509719
CD247	0.420418345	0.001616229
S100A16	0.410918297	0.002293413
SMS	0.424430778	0.001389172
DCTN6	0.421705189	0.001539849
C1QL3	-0.411435732	0.002250778
ATXN10	0.439076953	0.000782596
ICOSLG	-0.474097188	0.000172833
SIRPB1	-0.409268394	0.002433795
CLEC6A	-0.430607531	0.00109503
SELPLG.1	0.410799986	0.002303327
LILRA5.1	-0.408252243	0.002523977

Supplementary Table 12: Correlation coefficients and p-values of proteins most associated with plasma in the *LPS^{hi} Fatal* sub-group.

Pearson's correlation coefficients and two-tailed p-values of the 19 proteins most associated with plasma LPS among over 7300 SomaScan proteins in the LPS^{hi} Fatal sub-group (n=39). Correlation coefficients were inverse-proportionally weighted to account for selection biases. Only correlations with an absolute value of r ≥ 0.4 and p-value < 0.05 are displayed. All p-values were adjusted for false discovery rates using the Benjamini-Hochberg (BH) method, based on the full SomaScan dataset of over 7300 proteins. All n values refer to independent children; no technical replicates were used.

Characteristic	LPShi Fatal<br>N = 34[a]	LPShi Non-Fatal<br>N = 145[a]	LPSlo Fatal<br>N = 98[a]	LPSlo Non-Fatal<br>N = 1,609[a]	p-value[b]
Plasma LPS					<0.001
Mean (SD)	16.7 (4.6)	17.3 (4.6)	3.6 (3.2)	3.0 (3.1)	
Anthropometric classification					<0.001
Acute A (SAM)	18 / 34 (52%)	48 / 145 (33%)	64 / 98 (66%)	341 / 1,609 (21%)	
Acute B (MAM)	5 / 34 (15%)	29 / 145 (20%)	14 / 98 (14%)	258 / 1,609 (16%)	
Acute C (Normal)	11 / 34 (33%)	68 / 145 (47%)	20 / 98 (20%)	1,009 / 1,609 (63%)	
Age group					0.4
<6 months	8 / 34 (25%)	20 / 145 (14%)	21 / 98 (21%)	312 / 1,609 (19%)	
6-11 months	14 / 34 (41%)	68 / 145 (47%)	39 / 98 (40%)	566 / 1,609 (35%)	
12 months & above	11 / 34 (34%)	57 / 145 (40%)	37 / 98 (38%)	731 / 1,609 (45%)	
Admission age					0.5
Mean (SD)	10.2 (5.6)	10.8 (4.5)	11.0 (5.6)	11.6 (5.6)	
Sex					0.008
Female	19 / 34 (56%)	83 / 145 (57%)	47 / 98 (48%)	591 / 1,609 (37%)	
Male	15 / 34 (44%)	62 / 145 (43%)	51 / 98 (52%)	1,017 / 1,609 (63%)	
Continent					<0.001
Africa	30 / 34 (88%)	121 / 145 (84%)	71 / 98 (72%)	854 / 1,609 (53%)	
Asia	4 / 34 (12%)	23 / 145 (16%)	27 / 98 (28%)	755 / 1,609 (47%)	
Malaria	7 / 34 (20%)	29 / 145 (20%)	17 / 98 (17%)	278 / 1,609 (17%)	>0.9
Sepsis	10 / 34 (28%)	19 / 145 (13%)	24 / 98 (24%)	242 / 1,609 (15%)	0.2
Gastroenteritis	26 / 34 (77%)	91 / 145 (63%)	58 / 96 (60%)	964 / 1,589 (61%)	0.6
URTI	1 / 34 (4.0%)	11 / 145 (7.7%)	5 / 98 (5.1%)	81 / 1,609 (5.0%)	0.7
LRTI	13 / 34 (38%)	65 / 145 (45%)	40 / 98 (41%)	653 / 1,609 (41%)	0.9
HIV status					<0.001
neg	31 / 34 (90%)	139 / 145 (96%)	86 / 98 (88%)	1,579 / 1,609 (98%)	
pos	3 / 34 (9.9%)	5 / 145 (3.6%)	11 / 98 (12%)	30 / 1,609 (1.8%)	
TB	1 / 30 (2.8%)	0 / 119 (0%)	0 / 86 (0%)	2 / 1,466 (0.2%)	0.10
Breastfeeding status	26 / 34 (78%)	114 / 145 (79%)	59 / 98 (60%)	1,227 / 1,609 (76%)	0.084
Viruses	20 / 30 (69%)	67 / 125 (53%)	25 / 88 (28%)	664 / 1,460 (45%)	0.046
Parasites	9 / 31 (29%)	53 / 128 (41%)	27 / 92 (29%)	429 / 1,519 (28%)	0.2
Gram-negative Bacteria	33 / 34 (96%)	97 / 129 (75%)	87 / 95 (91%)	1,362 / 1,562 (87%)	0.081
Enteroaggregative E.coli					0.001
Mean (SD)	25.8 (5.8)	30.5 (5.9)	27.5 (5.9)	28.8 (6.4)	
Enterotoxigenic E.coli					>0.9
Mean (SD)	31.8 (5.8)	32.8 (5.2)	32.8 (4.4)	32.8 (4.6)	
Shigella toxin-producing Enterotoxigenic E.coli					0.8
Mean (SD)	32.8 (5.1)	33.0 (5.2)	33.4 (3.9)	33.6 (3.9)	
V.cholerae					>0.9
Mean (SD)	34.9 (0.4)	34.9 (0.6)	34.7 (1.8)	34.8 (1.6)	
CTX-M-producing bacteria					0.052
Mean (SD)	26.2 (5.8)	28.6 (6.5)	25.6 (6.2)	27.2 (6.8)	
mphA Azithromysin Resistance					<0.001
Mean (SD)	21.2 (5.2)	26.7 (7.1)	21.4 (5.9)	22.9 (6.4)	
X16s					<0.001
Mean (SD)	12.2 (1.9)	14.0 (3.5)	13.0 (3.0)	14.0 (3.3)	
Cryptosporidium					0.053
Mean (SD)	32.6 (4.9)	31.9 (6.0)	33.2 (4.7)	34.1 (2.9)	
Adenovirus 40/41					0.4
Mean (SD)	33.6 (4.6)	34.4 (3.5)	34.3 (2.7)	34.2 (3.2)	
Rotavirus					<0.001
Mean (SD)	31.9 (5.7)	33.2 (4.7)	34.5 (2.3)	32.9 (4.3)	
Sapovirus					0.2
Mean (SD)	34.2 (2.0)	34.4 (1.9)	34.6 (2.1)	34.7 (1.6)	

Fecal MPO					*0.10*
Mean (SD)	*4,747.1 (7,607.1)*	*2,653.3 (3,183.5)*	*5,227.1 (6,334.1)*	*4,163.6 (4,468.9)*	
Fecal Calprotectin					*0.003*
Mean (SD)	*852.4 (1,210.2)*	*328.3 (462.0)*	*895.7 (1,387.5)*	*586.3 (951.8)*	
Fecal AAT					*0.066*
Mean (SD)	*295.4 (502.2)*	*158.0 (236.1)*	*323.8 (529.9)*	*294.7 (427.4)*	
Plasma Zonulin					*0.021*
Mean (SD)	*33,736.2 (32,243.8)*	*38,441.4 (30,298.5)*	*42,406.5 (35,107.7)*	*47,717.2 (29,380.6)*	
Plasma Diamine Oxidase					*<0.001*
Mean (SD)	*3,377.3 (3,205.3)*	*4,433.3 (2,628.9)*	*4,808.8 (12,543.9)*	*4,834.4 (3,252.7)*	
Plasma FABP2					*0.5*
Mean (SD)	*3,703.8 (3,797.9)*	*3,689.6 (3,539.2)*	*3,551.2 (4,379.5)*	*3,133.7 (2,714.3)*	
Plasma CRP					*0.094*
Mean (SD)	*62,685.4 (31,276.2)*	*56,492.7 (34,998.5)*	*71,497.2 (32,896.9)*	*70,184.9 (35,755.3)*	
Plasma SAA1					*0.3*
Mean (SD)	*50,660.4 (53,468.9)*	*56,115.7 (62,615.3)*	*65,839.3 (61,976.0)*	*74,573.1 (73,751.4)*	
Plasma MPO					*0.002*
Mean (SD)	*35,788.1 (20,177.6)*	*24,180.2 (12,891.4)*	*34,692.0 (22,355.8)*	*27,326.2 (13,461.8)*	
Plasma Calprotectin					*0.5*
Mean (SD)	*2,041.2 (1,420.7)*	*1,910.6 (1,326.4)*	*1,764.2 (1,187.4)*	*1,756.3 (1,022.2)*	
Plasma AAT					*0.9*
Mean (SD)	*49,869.7 (11,679.6)*	*48,541.2 (13,458.9)*	*51,082.5 (17,252.0)*	*50,824.9 (15,878.9)*	
Plasma soluble CD14					*0.051*
Mean (SD)	*31,688.5 (8,472.0)*	*29,630.4 (6,590.8)*	*34,807.8 (12,240.3)*	*32,279.0 (8,127.2)*	
Plasma CD14					*<0.001*
Mean (SD)	*2,397.6 (1,344.7)*	*1,607.0 (605.7)*	*2,364.8 (1,620.1)*	*1,673.0 (617.4)*	
Plasma LPS Binding Protein					*0.001*
Mean (SD)	*77,694.5 (40,981.5)*	*53,099.1 (31,694.3)*	*76,151.9 (38,968.7)*	*67,794.0 (33,009.2)*	
Plasma TLR4					*0.019*
Mean (SD)	*379.6 (176.7)*	*438.6 (196.9)*	*673.4 (1,266.3)*	*511.7 (1,053.9)*	
Plasma Heparin-Binding Protein					*<0.001*
Mean (SD)	*31,240.7 (10,668.0)*	*26,322.3 (13,020.1)*	*29,175.7 (19,256.3)*	*23,707.3 (10,530.0)*	
Plasma TREM1					*<0.001*
Mean (SD)	*4,411.7 (4,014.6)*	*2,601.7 (1,686.2)*	*2,908.0 (2,404.2)*	*2,037.7 (1,177.0)*	
Plasma CDH1					*0.2*
Mean (SD)	*2,145.5 (567.9)*	*2,292.0 (476.7)*	*2,215.8 (1,014.3)*	*2,200.7 (483.1)*	
Plasma CDH1.1					*0.007*
Mean (SD)	*955.5 (409.8)*	*1,130.3 (474.6)*	*1,278.8 (1,012.4)*	*1,521.2 (2,872.5)*	
Plasma CDH1.2					*0.004*
Mean (SD)	*22,900.8 (8,445.9)*	*27,482.1 (7,827.2)*	*23,456.4 (8,073.5)*	*25,908.1 (6,163.6)*	
Plasma OCLN					*0.008*
Mean (SD)	*1,053.7 (386.2)*	*1,012.9 (520.2)*	*1,029.2 (331.7)*	*949.9 (254.8)*	
Plasma ZO-1					*<0.001*
Mean (SD)	*446.2 (126.4)*	*621.5 (615.5)*	*518.0 (510.9)*	*746.7 (1,846.2)*	
Plasma TNFAIP3					*0.003*
Mean (SD)	*1,459.9 (451.2)*	*1,305.2 (306.8)*	*1,627.1 (1,337.0)*	*1,657.9 (1,059.6)*	
Plasma SOCS-3					*0.11*
Mean (SD)	*1,337.89 (372.15)*	*1,385.58 (515.95)*	*1,728.89 (3,121.79)*	*1,477.47 (2,376.70)*	

[a]*N = inverse proportionally weighted total within CHAIN cohort (n=3101)*

[b]*Design-based Kruskal-Wallis test; Pearson's X^2: Rao & Scott adjustment*

Supplementary Table 13: Demographic, comorbidity frequencies, entero-pathogen quantification and fecal and plasma protein expression of LPS sub-groups

Demographics (age, sex, anthropometric classification, continent), fecal biomarkers (MPO, calprotectin, AAT), plasma LPS concentrations, comorbidities (malaria, sepsis, gastroenteritis, LRTI), barrier protein expression (zonulin, diamine oxidase) and plasma systemic inflammatory (CRP, SAA1, MPO, calprotectin, AAT) and LPS signal transduction

biomarkers (CD14, soluble CD14, LPS binding protein) comparison. Both unweighted and inverse proportionally weighted totals (N) are presented. Unpaired, two-tailed Wilcoxon rank-sum tests were used for comparisons of continuous variables, while chi-square tests were used for categorical variables. n values refer to independent participants; no technical replicates were used. Only entero-pathogen with significant difference in expression between the cohorts are presented. See Supplementary Table 11 for all pathogen levels. LPS = lipopolysaccharide, MPO = myeloperoxidase, AAT = Alpha-1-antitrypsin, FABP2 = Intestinal-type fatty acid-binding protein, TLR4 = Toll-like Receptor 4.

LPS-mortality sub-group	emmean	SE	df	lower.CL	upper.CL	Protein
LPShi Fatal	16.49095	0.814201	620	14.89203	18.08988	Plasma LPS
LPShi Non-Fatal	17.14094	0.804852	620	15.56037	18.7215	Plasma LPS
LPSlo Fatal	3.640725	0.391066	620	2.872751	4.408698	Plasma LPS
LPSlo Non-Fatal	3.262385	0.328504	620	2.61727	3.907499	Plasma LPS
LPShi Fatal	11.71576	0.395279	546	10.93931	12.49221	X16s
LPShi Non-Fatal	13.5623	0.599685	546	12.38433	14.74027	X16s
LPSlo Fatal	12.57288	0.343029	546	11.89906	13.24669	X16s
LPSlo Non-Fatal	13.6671	0.273985	546	13.12891	14.20529	X16s
LPShi Fatal	34.95407	0.038782	614	34.87791	35.03023	Aeromonas
LPShi Non-Fatal	34.94148	0.044655	614	34.85378	35.02918	Aeromonas
LPSlo Fatal	34.83307	0.070533	614	34.69455	34.97158	Aeromonas
LPSlo Non-Fatal	34.90569	0.046586	614	34.8142	34.99717	Aeromonas
LPShi Fatal	30.43563	1.07995	613	28.31478	32.55648	Campylobacter_pan
LPShi Non-Fatal	32.32064	0.826065	613	30.69838	33.9429	Campylobacter_pan
LPSlo Fatal	31.19213	0.699816	613	29.8178	32.56646	Campylobacter_pan
LPSlo Non-Fatal	31.92167	0.629511	613	30.68541	33.15793	Campylobacter_pan
LPShi Fatal	31.20871	0.974209	613	29.29552	33.12191	Campylobacter_jejuni_coli
LPShi Non-Fatal	33.31213	0.640532	613	32.05423	34.57004	Campylobacter_jejuni_coli
LPSlo Fatal	32.40704	0.617741	613	31.19389	33.62018	Campylobacter_jejuni_coli
LPSlo Non-Fatal	33.04209	0.573196	613	31.91643	34.16776	Campylobacter_jejuni_coli
LPShi Fatal	25.70849	1.187183	614	23.37706	28.03992	EAEC
LPShi Non-Fatal	30.25354	1.021526	614	28.24744	32.25965	EAEC
LPSlo Fatal	27.30321	0.742605	614	25.84486	28.76156	EAEC
LPSlo Non-Fatal	28.84558	0.636927	614	27.59476	30.0964	EAEC
LPShi Fatal	31.89881	1.054502	614	29.82794	33.96968	tEPEC
LPShi Non-Fatal	32.20544	0.892949	614	30.45183	33.95904	tEPEC
LPSlo Fatal	31.71218	0.759452	614	30.22074	33.20362	tEPEC
LPSlo Non-Fatal	33.43478	0.581309	614	32.29318	34.57637	tEPEC
LPShi Fatal	32.60549	0.740515	612	31.15123	34.05974	aEPEC
LPShi Non-Fatal	33.19467	0.661342	612	31.89589	34.49344	aEPEC
LPSlo Fatal	33.67542	0.476546	612	32.73955	34.61128	aEPEC
LPSlo Non-Fatal	32.7229	0.608053	612	31.52878	33.91702	aEPEC
LPShi Fatal	31.82195	1.255017	614	29.35731	34.2866	ETEC
LPShi Non-Fatal	32.74125	0.888973	614	30.99546	34.48705	ETEC
LPSlo Fatal	33.03933	0.519081	614	32.01994	34.05872	ETEC
LPSlo Non-Fatal	33.10246	0.372186	614	32.37155	33.83337	ETEC
LPShi Fatal	32.59311	1.183746	614	30.26843	34.9178	ST_ETEC
LPShi Non-Fatal	32.76131	0.872199	614	31.04846	34.47417	ST_ETEC
LPSlo Fatal	33.41263	0.448597	614	32.53165	34.2936	ST_ETEC
LPSlo Non-Fatal	33.76962	0.314438	614	33.15211	34.38712	ST_ETEC
LPShi Fatal	34.32531	0.50295	614	33.3376	35.31302	LT_ETEC
LPShi Non-Fatal	34.95258	0.270199	614	34.42196	35.48321	LT_ETEC
LPSlo Fatal	34.65356	0.283554	614	34.09671	35.21042	LT_ETEC
LPSlo Non-Fatal	34.40421	0.206585	614	33.99851	34.80991	LT_ETEC
LPShi Fatal	34.64889	0.202514	613	34.25118	35.04659	H_pylori
LPShi Non-Fatal	34.9207	0.084479	613	34.75479	35.0866	H_pylori
LPSlo Fatal	34.99964	0.022483	613	34.95548	35.04379	H_pylori
LPSlo Non-Fatal	34.99377	0.019022	613	34.95641	35.03112	H_pylori
LPShi Fatal	34.71901	0.275877	543	34.17709	35.26093	Plesiomonas
LPShi Non-Fatal	34.64355	0.300927	543	34.05243	35.23467	Plesiomonas
LPSlo Fatal	34.68046	0.273771	543	34.14268	35.21824	Plesiomonas
LPSlo Non-Fatal	34.5927	0.323675	543	33.95689	35.22851	Plesiomonas
LPShi Fatal	34.77303	0.211915	542	34.35675	35.18931	Salmonella
LPShi Non-Fatal	34.9655	0.092369	542	34.78406	35.14695	Salmonella
LPSlo Fatal	34.84112	0.128805	542	34.5881	35.09414	Salmonella
LPSlo Non-Fatal	34.88238	0.071703	542	34.74153	35.02323	Salmonella
LPShi Fatal	31.39905	1.439534	544	28.57132	34.22677	Shigella_EIEC
LPShi Non-Fatal	33.6673	0.789302	544	32.11684	35.21775	Shigella_EIEC
LPSlo Fatal	32.26964	0.767118	544	30.76276	33.77652	Shigella_EIEC
LPSlo Non-Fatal	32.93531	0.765787	544	31.43105	34.43957	Shigella_EIEC
LPShi Fatal	35.00972	0.020233	544	34.96997	35.04946	STEC
LPShi Non-Fatal	35.00505	0.028827	544	34.94843	35.06168	STEC
LPSlo Fatal	34.98204	0.022542	544	34.93776	35.02632	STEC
LPSlo Non-Fatal	34.9707	0.019954	544	34.93151	35.0099	STEC
LPShi Fatal	34.84828	0.0981	544	34.65557	35.04098	V_cholerae
LPShi Non-Fatal	34.8573	0.090436	544	34.67965	35.03494	V_cholerae
LPSlo Fatal	34.75695	0.161065	544	34.44057	35.07334	V_cholerae
LPSlo Non-Fatal	34.91004	0.053502	544	34.80494	35.01514	V_cholerae

LPS-mortality sub-group	*emmean*	*SE*	*df*	*lower.CL*	*upper.CL*	*Protein*
LPShi Fatal	*22.15731*	*1.143491*	*544*	*19.91111*	*24.40351*	*CTX_M*
LPShi Non-Fatal	*25.20894*	*1.133517*	*544*	*22.98234*	*27.43555*	*CTX_M*
LPSlo Fatal	*23.05805*	*0.965512*	*544*	*21.16146*	*24.95464*	*CTX_M*
LPSlo Non-Fatal	*26.06011*	*0.824986*	*544*	*24.43957*	*27.68066*	*CTX_M*
LPShi Fatal	*20.02153*	*1.204971*	*544*	*17.65456*	*22.38849*	*mphA_Azithromysin.resistance*
LPShi Non-Fatal	*25.57236*	*1.3808*	*544*	*22.86001*	*28.28471*	*mphA_Azithromysin.resistance*
LPSlo Fatal	*20.8319*	*0.90609*	*544*	*19.05204*	*22.61177*	*mphA_Azithromysin.resistance*
LPSlo Non-Fatal	*23.06043*	*0.874564*	*544*	*21.34249*	*24.77837*	*mphA_Azithromysin.resistance*
LPShi Fatal	*34.94809*	*0.06921*	*614*	*34.81217*	*35.084*	*C_difficile*
LPShi Non-Fatal	*34.94663*	*0.07805*	*614*	*34.79335*	*35.09991*	*C_difficile*
LPSlo Fatal	*34.80809*	*0.136603*	*614*	*34.53983*	*35.07635*	*C_difficile*
LPSlo Non-Fatal	*34.86454*	*0.073534*	*614*	*34.72013*	*35.00895*	*C_difficile*
LPShi Fatal	*35*	*9.32E-14*	*614*	*35*	*35*	*Ancylostoma*
LPShi Non-Fatal	*35*	*9.27E-14*	*614*	*35*	*35*	*Ancylostoma*
LPSlo Fatal	*35*	*5.85E-14*	*614*	*35*	*35*	*Ancylostoma*
LPSlo Non-Fatal	*35*	*5.17E-14*	*614*	*35*	*35*	*Ancylostoma*
LPShi Fatal	*35.00393*	*0.003716*	*614*	*34.99663*	*35.01122*	*Ascaris*
LPShi Non-Fatal	*35.0037*	*0.003132*	*614*	*34.99755*	*35.00985*	*Ascaris*
LPSlo Fatal	*34.98117*	*0.018942*	*614*	*34.94397*	*35.01836*	*Ascaris*
LPSlo Non-Fatal	*35.00113*	*0.00247*	*614*	*34.99628*	*35.00599*	*Ascaris*
LPShi Fatal	*35*	*9.32E-14*	*614*	*35*	*35*	*Cyclospora*
LPShi Non-Fatal	*35*	*9.27E-14*	*614*	*35*	*35*	*Cyclospora*
LPSlo Fatal	*35*	*5.85E-14*	*614*	*35*	*35*	*Cyclospora*
LPSlo Non-Fatal	*35*	*5.17E-14*	*614*	*35*	*35*	*Cyclospora*
LPShi Fatal	*34.6255*	*0.254364*	*614*	*34.12598*	*35.12503*	*E_bieneusi*
LPShi Non-Fatal	*34.70646*	*0.197814*	*614*	*34.31799*	*35.09494*	*E_bieneusi*
LPSlo Fatal	*34.85644*	*0.121427*	*614*	*34.61798*	*35.0949*	*E_bieneusi*
LPSlo Non-Fatal	*34.76171*	*0.13911*	*614*	*34.48852*	*35.0349*	*E_bieneusi*
LPShi Fatal	*35*	*5.37E-14*	*613*	*35*	*35*	*E_histolytica*
LPShi Non-Fatal	*35*	*5.44E-14*	*613*	*35*	*35*	*E_histolytica*
LPSlo Fatal	*35*	*3.39E-14*	*613*	*35*	*35*	*E_histolytica*
LPSlo Non-Fatal	*35*	*2.97E-14*	*613*	*35*	*35*	*E_histolytica*
LPShi Fatal	*34.999*	*0.006119*	*614*	*34.98698*	*35.01102*	*E_intestinalis*
LPShi Non-Fatal	*35.00231*	*0.006313*	*614*	*34.98991*	*35.01471*	*E_intestinalis*
LPSlo Fatal	*35.00356*	*0.005204*	*614*	*34.99334*	*35.01378*	*E_intestinalis*
LPSlo Non-Fatal	*34.99215*	*0.0085*	*614*	*34.97546*	*35.00885*	*E_intestinalis*
LPShi Fatal	*34.96554*	*0.279403*	*614*	*34.41684*	*35.51424*	*Giardia*
LPShi Non-Fatal	*34.26997*	*0.433844*	*614*	*33.41798*	*35.12197*	*Giardia*
LPSlo Fatal	*34.72821*	*0.251234*	*614*	*34.23483*	*35.22159*	*Giardia*
LPSlo Non-Fatal	*34.20812*	*0.193785*	*614*	*33.82756*	*34.58868*	*Giardia*
LPShi Fatal	*35.00531*	*0.006607*	*544*	*34.99233*	*35.01829*	*Isospora*
LPShi Non-Fatal	*34.96084*	*0.038236*	*544*	*34.88573*	*35.03595*	*Isospora*
LPSlo Fatal	*35.00429*	*0.004904*	*544*	*34.99466*	*35.01392*	*Isospora*
LPSlo Non-Fatal	*35.00072*	*0.003269*	*544*	*34.9943*	*35.00715*	*Isospora*
LPShi Fatal	*34.99803*	*0.003626*	*544*	*34.9909*	*35.00515*	*Necator*
LPShi Non-Fatal	*34.99938*	*0.003121*	*544*	*34.99325*	*35.00551*	*Necator*
LPSlo Fatal	*35.00341*	*0.004445*	*544*	*34.99468*	*35.01214*	*Necator*
LPSlo Non-Fatal	*34.99548*	*0.004676*	*544*	*34.98629*	*35.00466*	*Necator*
LPShi Fatal	*35*	*2.67E-14*	*542*	*35*	*35*	*Strongyloides*
LPShi Non-Fatal	*35*	*2.62E-14*	*542*	*35*	*35*	*Strongyloides*
LPSlo Fatal	*35*	*1.75E-14*	*542*	*35*	*35*	*Strongyloides*
LPSlo Non-Fatal	*35*	*1.56E-14*	*542*	*35*	*35*	*Strongyloides*
LPShi Fatal	*35*	*3.54E-14*	*544*	*35*	*35*	*Trichuris*
LPShi Non-Fatal	*35*	*3.44E-14*	*544*	*35*	*35*	*Trichuris*
LPSlo Fatal	*35*	*2.35E-14*	*544*	*35*	*35*	*Trichuris*
LPSlo Non-Fatal	*35*	*2.10E-14*	*544*	*35*	*35*	*Trichuris*
LPShi Fatal	*32.18654*	*0.900993*	*614*	*30.41714*	*33.95594*	*Cryptosporidium*
LPShi Non-Fatal	*31.44635*	*1.140719*	*614*	*29.20617*	*33.68654*	*Cryptosporidium*
LPSlo Fatal	*32.83876*	*0.577563*	*614*	*31.70452*	*33.973*	*Cryptosporidium*
LPSlo Non-Fatal	*33.44474*	*0.538053*	*614*	*32.38809*	*34.50138*	*Cryptosporidium*
LPShi Fatal	*33.39596*	*0.73185*	*608*	*31.9587*	*34.83322*	*Adenovirus_40_41*
LPShi Non-Fatal	*34.12433*	*0.608884*	*608*	*32.92855*	*35.3201*	*Adenovirus_40_41*
LPSlo Fatal	*34.14381*	*0.455729*	*608*	*33.24882*	*35.03881*	*Adenovirus_40_41*
LPSlo Non-Fatal	*34.09159*	*0.458521*	*608*	*33.19112*	*34.99207*	*Adenovirus_40_41*
LPShi Fatal	*34.79838*	*0.176944*	*610*	*34.45089*	*35.14587*	*Astrovirus*
LPShi Non-Fatal	*34.13569*	*0.483243*	*610*	*33.18667*	*35.08472*	*Astrovirus*
LPSlo Fatal	*34.70748*	*0.207112*	*610*	*34.30074*	*35.11422*	*Astrovirus*
LPSlo Non-Fatal	*34.91187*	*0.078858*	*610*	*34.757*	*35.06673*	*Astrovirus*

LPS-mortality sub-group	emmean	SE	df	lower.CL	upper.CL	Protein
LPShi Fatal	34.93153	0.107604	540	34.72016	35.1429	Norovirus_GI
LPShi Non-Fatal	35.04231	0.048438	540	34.94716	35.13746	Norovirus_GI
LPSlo Fatal	34.94413	0.081096	540	34.78483	35.10343	Norovirus_GI
LPSlo Non-Fatal	34.96236	0.029662	540	34.90409	35.02062	Norovirus_GI
LPShi Fatal	34.03836	0.502442	540	33.05138	35.02534	Norovirus_GII
LPShi Non-Fatal	33.72984	0.69616	540	32.36232	35.09735	Norovirus_GII
LPSlo Fatal	34.4064	0.375684	540	33.66842	35.14438	Norovirus_GII
LPSlo Non-Fatal	34.2705	0.345241	540	33.59232	34.94868	Norovirus_GII
LPShi Fatal	32.15078	1.273753	543	29.64869	34.65287	Rotavirus
LPShi Non-Fatal	33.54014	0.892922	543	31.78614	35.29415	Rotavirus
LPSlo Fatal	34.94453	0.336185	543	34.28414	35.60491	Rotavirus
LPSlo Non-Fatal	33.86875	0.222329	543	33.43202	34.30548	Rotavirus
LPShi Fatal	34.38217	0.35294	541	33.68887	35.07548	Sapovirus
LPShi Non-Fatal	34.64074	0.301176	541	34.04913	35.23236	Sapovirus
LPSlo Fatal	34.81102	0.1926	541	34.43268	35.18935	Sapovirus
LPSlo Non-Fatal	34.99216	0.095136	541	34.80528	35.17904	Sapovirus
LPShi Fatal	34.91243	0.087605	544	34.74035	35.08452	M_tuberculosis
LPShi Non-Fatal	35.00598	0.004984	544	34.99619	35.01577	M_tuberculosis
LPSlo Fatal	35.00355	0.003188	544	34.99729	35.00981	M_tuberculosis
LPSlo Non-Fatal	34.99892	0.003157	544	34.99271	35.00512	M_tuberculosis
LPShi Fatal	5271.467	1203.956	618	2907.126	7635.807	Fecal MPO
LPShi Non-Fatal	3505.604	706.5668	618	2118.041	4893.167	Fecal MPO
LPSlo Fatal	5835.133	662.0694	618	4534.955	7135.312	Fecal MPO
LPSlo Non-Fatal	5467.057	574.5151	618	4338.819	6595.296	Fecal MPO
LPShi Fatal	976.3094	210.1796	591	563.5196	1389.099	Fecal Calprotectin
LPShi Non-Fatal	458.2099	128.4371	591	205.9612	710.4586	Fecal Calprotectin
LPSlo Fatal	1013.107	138.6806	591	740.7398	1285.473	Fecal Calprotectin
LPSlo Non-Fatal	807.7661	115.4578	591	581.0085	1034.524	Fecal Calprotectin
LPShi Fatal	248.5035	115.1273	615	22.41311	474.5938	Fecal AAT
LPShi Non-Fatal	116.9718	50.81254	615	17.18467	216.7589	Fecal AAT
LPSlo Fatal	266.8986	50.30456	615	168.109	365.6881	Fecal AAT
LPSlo Non-Fatal	260.3818	35.04909	615	191.5514	329.2122	Fecal AAT
LPShi Fatal	3522.298	790.0925	620	1970.716	5073.88	AOC1
LPShi Non-Fatal	4665.129	800.0114	620	3094.069	6236.189	AOC1
LPSlo Fatal	4966.263	1058.403	620	2887.775	7044.752	AOC1
LPSlo Non-Fatal	5099.744	702.7762	620	3719.634	6479.854	AOC1
LPShi Fatal	38196.6	5397.777	620	27596.45	48796.74	HP
LPShi Non-Fatal	42549.96	5163.598	620	32409.7	52690.22	HP
LPSlo Fatal	48338.2	3929.04	620	40622.36	56054.04	HP
LPSlo Non-Fatal	54242.09	2992.724	620	48364.99	60119.2	HP
LPShi Fatal	3860.64	645.9677	620	2592.09	5129.19	FABP2
LPShi Non-Fatal	3887.279	514.9168	620	2876.086	4898.471	FABP2
LPSlo Fatal	3772.243	448.7681	620	2890.954	4653.533	FABP2
LPSlo Non-Fatal	3443.336	366.9647	620	2722.692	4163.981	FABP2
LPShi Fatal	47957.02	2877.926	620	42305.36	53608.68	SERPINA1
LPShi Non-Fatal	45731.19	2274.734	620	41264.07	50198.31	SERPINA1
LPSlo Fatal	49334.01	1817.981	620	45763.86	52904.16	SERPINA1
LPSlo Non-Fatal	50218.45	1472.58	620	47326.6	53110.3	SERPINA1
LPShi Fatal	37822.75	4012.36	620	29943.28	45702.21	Plasma MPO
LPShi Non-Fatal	25951.39	2214.609	620	21602.34	30300.43	Plasma MPO
LPSlo Fatal	35842.59	2512.159	620	30909.22	40775.96	Plasma MPO
LPSlo Non-Fatal	29449.15	1398.362	620	26703.05	32195.25	Plasma MPO
LPShi Fatal	2057.347	239.7289	620	1586.568	2528.126	S100A8.S100A9
LPShi Non-Fatal	1928.172	225.6063	620	1485.126	2371.217	S100A8.S100A9
LPSlo Fatal	1810.991	150.7744	620	1514.901	2107.082	S100A8.S100A9
LPSlo Non-Fatal	1844.452	136.2913	620	1576.803	2112.1	S100A8.S100A9
LPShi Fatal	48805.84	11248.95	620	26715.17	70896.5	SAA1
LPShi Non-Fatal	54494.57	12858.62	620	29242.84	79746.29	SAA1
LPSlo Fatal	63948.53	7542.136	620	49137.3	78759.76	SAA1
LPSlo Non-Fatal	73224.38	6545.957	620	60369.45	86079.32	SAA1
LPShi Fatal	56295.46	6478.36	620	43573.27	69017.65	SAA4
LPShi Non-Fatal	59928.01	5329.084	620	49462.77	70393.26	SAA4
LPSlo Fatal	55822.85	4284.762	620	47408.45	64237.26	SAA4
LPSlo Non-Fatal	60670.85	3290.43	620	54209.12	67132.59	SAA4
LPShi Fatal	67996.7	5928.356	620	56354.61	79638.79	CRP
LPShi Non-Fatal	63790.28	6936.34	620	50168.71	77411.85	CRP
LPSlo Fatal	75065.63	3820.478	620	67562.98	82568.27	CRP
LPSlo Non-Fatal	76178.16	3155.204	620	69981.98	82374.35	CRP

LPS-mortality sub-group	emmean	SE	df	lower.CL	upper.CL	Protein
LPShi Fatal	32754.3	1391.334	620	30022	35486.6	CD14
LPShi Non-Fatal	30408.47	1522.363	620	27418.85	33398.08	CD14
LPSlo Fatal	35182.71	1346.071	620	32539.3	37826.12	CD14
LPSlo Non-Fatal	32948.31	1022.744	620	30939.85	34956.78	CD14
LPShi Fatal	2439.402	232.4946	620	1982.83	2895.975	CD14.1
LPShi Non-Fatal	1675.588	108.4508	620	1462.613	1888.563	CD14.1
LPSlo Fatal	2385.713	152.6105	620	2086.016	2685.409	CD14.1
LPSlo Non-Fatal	1776.487	69.3787	620	1640.241	1912.732	CD14.1
LPShi Fatal	482.4427	58.26043	620	368.031	596.8544	TLR4
LPShi Non-Fatal	562.3748	61.45923	620	441.6813	683.0683	TLR4
LPSlo Fatal	756.8039	143.3964	620	475.2023	1038.405	TLR4
LPSlo Non-Fatal	595.7125	60.29458	620	477.3061	714.1189	TLR4
LPShi Fatal	78115.81	8484.298	620	61454.36	94777.25	LBP
LPShi Non-Fatal	55134.03	5162.795	620	44995.35	65272.72	LBP
LPSlo Fatal	75034.52	4293.617	620	66602.72	83466.31	LBP
LPSlo Non-Fatal	69843.02	2475.103	620	64982.42	74703.63	LBP
LPShi Fatal	30398.48	2157.939	620	26160.73	34636.24	LY6G6C
LPShi Non-Fatal	26549.44	2029.282	620	22564.34	30534.54	LY6G6C
LPSlo Fatal	28203.34	1900.404	620	24471.33	31935.35	LY6G6C
LPSlo Non-Fatal	24077.55	1130.382	620	21857.71	26297.39	LY6G6C
LPShi Fatal	4428.34	652.212	620	3147.528	5709.152	TREM1
LPShi Non-Fatal	2667.207	278.959	620	2119.388	3215.026	TREM1
LPSlo Fatal	3035.62	248.5522	620	2547.514	3523.726	TREM1
LPSlo Non-Fatal	2321.734	158.5145	620	2010.444	2633.025	TREM1
LPShi Fatal	2216.257	126.5086	620	1967.82	2464.694	CDH1
LPShi Non-Fatal	2351.28	104.6388	620	2145.791	2556.769	CDH1
LPSlo Fatal	2281.187	106.7345	620	2071.582	2490.792	CDH1
LPSlo Non-Fatal	2280.356	78.87927	620	2125.453	2435.258	CDH1
LPShi Fatal	792.1443	167.327	620	463.548	1120.741	CDH1.1
LPShi Non-Fatal	1080.324	150.3558	620	785.0552	1375.592	CDH1.1
LPSlo Fatal	1175.457	137.6508	620	905.1385	1445.775	CDH1.1
LPSlo Non-Fatal	1522.406	153.6457	620	1220.677	1824.135	CDH1.1
LPShi Fatal	23784.33	1787.851	620	20273.36	27295.31	CDH1.2
LPShi Non-Fatal	27812.44	1493.946	620	24878.63	30746.24	CDH1.2
LPSlo Fatal	23758.11	948.8791	620	21894.7	25621.51	CDH1.2
LPSlo Non-Fatal	25907.43	840.1617	620	24257.52	27557.34	CDH1.2
LPShi Fatal	1102.584	78.45241	620	948.519	1256.648	OCLN
LPShi Non-Fatal	1064.997	88.10381	620	891.9794	1238.015	OCLN
LPSlo Fatal	1067.746	54.68506	620	960.3557	1175.137	OCLN
LPSlo Non-Fatal	1006.435	62.60123	620	883.4992	1129.372	OCLN
LPShi Fatal	318.7759	127.9006	620	67.60506	569.9467	TJP1
LPShi Non-Fatal	458.2319	160.5307	620	142.9822	773.4816	TJP1
LPSlo Fatal	306.7919	175.7568	620	-38.3589	651.9426	TJP1
LPSlo Non-Fatal	435.7485	145.2673	620	150.4728	721.0242	TJP1
LPShi Fatal	1516.459	99.74649	620	1320.577	1712.341	TNFAIP3
LPShi Non-Fatal	1331.27	70.39672	620	1193.025	1469.515	TNFAIP3
LPSlo Fatal	1673.35	110.1224	620	1457.092	1889.608	TNFAIP3
LPSlo Non-Fatal	1663.124	75.84454	620	1514.181	1812.067	TNFAIP3
LPShi Fatal	1441.47	209.574	620	1029.909	1853.031	SOCS3
LPShi Non-Fatal	1516.718	252.635	620	1020.594	2012.842	SOCS3
LPSlo Fatal	1811.376	407.323	620	1011.476	2611.276	SOCS3
LPSlo Non-Fatal	1641.493	230.3587	620	1189.115	2093.871	SOCS3

Supplementary Table 14 (Adjusted) – Predicted means of Entero-pathogen Quantification, and Fecal and Plasma Protein Expression across LPS sub-groups (Adjusted Models)

Survey-weighted generalized linear models were used to estimate adjusted means for plasma LPS concentrations, fecal biomarkers (MPO, calprotectin, AAT), barrier function proteins (zonulin, diamine oxidase), systemic inflammatory markers (CRP, SAA1, MPO, calprotectin, AAT), and LPS signal transduction markers (CD14, soluble CD14, LBP) for admission children (n = 635). Models were adjusted for age, sex, HIV status, and study site. Values represent marginal means with standard errors generated using the emmeans package. Pairwise contrasts were tested using two-tailed comparisons, with Holm adjustment applied for multiple testing. All n values refer to independent children; no technical replicates were used. LPS = lipopolysaccharide, MPO = myeloperoxidase, AAT = alpha-1-antitrypsin.

Biomarkers	LPShi Non-Fatal - LPSlo Non-Fatal	LPShi Non-Fatal - LPSlo Fatal	LPShi Fatal - LPShi Non-Fatal	LPShi Fatal - LPSlo Non-Fatal	LPShi Fatal - LPSlo Fatal	LPSlo Fatal - LPSlo Non-Fatal
Plasma LPS	3.95615E-54	3.6665E-49	0.570220724	2.70608E-48	1.10054E-44	0.570220724
Fecal AAT	0.026432227	0.102058595	1	1	1	1
Fecal Calprotectin	0.009957584	0.00084603	0.066608417	0.88114244	0.88114244	0.466949685
Fecal MPO	0.005660346	0.009116783	0.033707639	0.113783087	0.666968804	0.049082187
Adenovirus_40_41	1	1	1	1	1	1
aEPEC	1	1	1	1	0.333076372	0.002962578
Aeromonas	0.541425598	0.292605332	0.568294545	0.264573614	0.254138646	0.541425598
Ancylostoma	1	1	1	1	1	1
Ascaris	1	1	1	1	1	1
Astrovirus	0.744483705	1	1	1	1	1
C_difficile	0.365260582	0.365260582	0.958411786	0.365260582	0.365260582	0.933629157
Campylobacter_jejuni_coli	0.611991077	0.492709207	0.168168158	0.219751799	0.611991077	0.611991077
Campylobacter_pan	0.989701812	0.702888451	0.60085386	0.702888451	0.989701812	0.702888451
Cryptosporidium	0.421157866	0.674125112	0.932219965	0.673269097	0.932219965	0.673269097
CTX_M	0.707775227	0.146323566	0.057795075	0.000418721	0.707775227	0.000418721
Cyclospora	1	1	1	1	1	1
E_bieneusi	1	1	1	1	1	1
E_histolytica	1	1	1	1	1	1
E_intestinalis	1	1	1	1	1	1
EAEC	0.287504654	0.030475725	0.007676441	0.030475725	0.287504654	0.091570578
ETEC	1	1	1	1	1	1
Giardia	1	1	0.651307068	0.056070967	1	0.293189857
H_pylori	1	1	0.923645959	0.577641976	0.577641976	1
Isospora	1	1	1	1	1	1
LT_ETEC	0.059772539	1	1	1	1	1
M_tuberculosis	1	1	1	1	1	1
mphA_Azithromysin.resistance	0.092319743	0.001704832	0.001684302	0.0163795	0.465645444	0.00663813
Necator	1	1	1	1	1	1
Norovirus_GI	1	1	1	1	1	1
Norovirus_GII	1	1	1	1	1	1
Plesiomonas	1	1	1	0.58953748	1	1
Rotavirus	0.77905432	0.638883448	0.77905432	0.638883448	0.166014074	0.010758844
Salmonella	1	1	1	1	1	1
Sapovirus	1	1	1	0.573422769	1	1
Shigella_EIEC	0.69150965	0.241685901	0.506153784	0.78285338	0.78285338	0.78285338
ST_ETEC	1	1	1	1	1	1
STEC	1	1	1	1	1	1
Strongyloides	1	1	1	1	1	1

Biomarkers	LPShi Non-Fatal - LPSlo Non-Fatal	LPShi Non-Fatal - LPSlo Fatal	LPShi Fatal - LPShi Non-Fatal	LPShi Fatal - LPSlo Non-Fatal	LPShi Fatal - LPSlo Fatal	LPSlo Fatal - LPSlo Non-Fatal
tEPEC	0.580114293	1	1	0.580114293	1	0.10294383
Trichuris	1	1	1	1	1	1
V_cholerae	1	1	1	1	1	1
X16s	0.86117197	0.234512554	0.015352951	3.99222E-06	0.150723322	0.008597225
AOC1	1	1	0.512660154	0.046348818	0.947909458	1
CD14	0.221369877	0.019078979	0.31964989	0.87388688	0.31964989	0.214488076
CD14.1	0.629863458	0.000234634	0.008458438	0.01392935	0.843438504	0.000261252
CDH1	1	1	1	1	1	1
CDH1.1	0.130406271	0.549030367	0.222736551	0.004143086	0.050898529	0.222736551
CDH1.2	0.524186671	0.046047526	0.204070373	0.524186671	0.988052938	0.046047526
CRP	0.440549966	0.587695394	1	0.677168157	0.770102269	1
FABP2	1	1	1	1	1	1
HP	0.094679605	0.614212921	0.614212921	0.014243627	0.316805231	0.358347683
LBP	0.030967784	0.011676419	0.073034158	0.724805108	0.7379949	0.724805108
LY6G6C	0.62944024	0.831973446	0.613658072	0.018106487	0.831973446	0.154259809
OCLN	1	1	1	0.612929245	1	0.533117122
S100A8.S100A9	1	1	1	1	1	1
SAA1	0.700565953	0.94469485	0.94469485	0.151699985	0.736609698	0.736609698
SAA4	1	1	1	1	1	1
SERPINA1	0.171778101	0.661540877	1	1	1	1
SOCS3	1	0.927626159	1	0.834854867	0.834854867	1
TJP1	1	0.739456892	0.739456892	0.672680669	1	0.739456892
TLR4	0.500391172	0.474025986	0.234415307	0.092938464	0.234415307	0.500391172
TNFAIP3	0.001284569	0.02244176	0.350038649	0.578763747	0.578763747	0.932751018
TREM1	0.362873603	0.362873603	0.040640416	0.006248109	0.114347472	0.008102687

Supplementary Table 15 – Pairwise Comparisons of adjusted means for Entero-pathogen Quantification and Fecal and Plasma Protein Expression Across LPS sub-groups

Pairwise comparisons were performed to assess differences in model-adjusted means between LPS sub-groups for plasma LPS concentrations, fecal biomarkers (MPO, calprotectin, AAT), barrier function proteins (zonulin, diamine oxidase), systemic inflammatory markers (CRP, SAA1, MPO, calprotectin, AAT), and LPS signal transduction markers (CD14, soluble CD14, LBP) for admission children (n=635). P-values were calculated from two-tailed t-tests for contrasts of estimated marginal means derived from survey-weighted generalized linear models, with Holm adjustment for multiple comparisons. Models were adjusted for age, sex, HIV status, and study site. These values represent the statistical significance of differences in adjusted means across LPS sub-groups. All n values refer to independent children; no technical replicates were used.

LPS = lipopolysaccharide, MPO = myeloperoxidase, AAT = alpha-1-antitrypsin, LBP = LPS-binding protein.

	LPSHI F (SD)	LPSLO F (SD)	LPSHI NF (SD)	LPSLO NF (SD)
**CA6**	9.578 (1.189)	9.759 (1.16)	10.445 (1.197)	10.568 (1.214)
**CA6.1**	8.361 (1.724)	8.776 (1.685)	9.979 (1.73)	9.987 (1.67)
**CDON**	11.963 (0.921)	12.519 (1.012)	12.61 (0.928)	12.983 (0.968)
**COL10A1**	11.585 (1.601)	11.839 (1.746)	12.951 (1.547)	13.324 (1.555)
**COL1A1**	12.694 (1.71)	13.086 (1.687)	14.135 (1.411)	14.345 (1.521)
**DEFA3**	15.123 (1.404)	14.262 (1.69)	13.538 (1.466)	13.331 (1.33)
**FTH1.FTL**	13.687 (1.739)	13.664 (1.693)	12.511 (1.963)	12.764 (1.711)
**FTL**	13.83 (1.717)	13.792 (1.677)	12.69 (1.912)	12.891 (1.699)
**HAMP**	12.59 (2.191)	11.812 (2.083)	11.36 (2.305)	11.545 (1.828)
**IGF1**	10.876 (0.856)	11.204 (0.925)	11.859 (0.894)	12.127 (1.0)
**IGF2**	11.146 (1.193)	11.679 (1.158)	12.602 (0.92)	12.824 (1.112)
**IGFBP3**	12.007 (0.844)	12.23 (0.886)	12.922 (0.914)	13.206 (0.994)
**IGLL1**	11.546 (1.895)	11.554 (1.739)	12.836 (1.634)	13.247 (1.678)
**IL1RL1**	15.057 (1.267)	14.695 (1.661)	13.716 (1.404)	13.519 (1.402)
**IL1RN**	13.588 (1.358)	12.742 (1.242)	12.311 (1.111)	12.275 (1.106)
**LCN2**	11.506 (1.612)	10.773 (1.535)	10.549 (1.258)	9.844 (1.104)
**LTA.LTB**	7.383 (0.595)	7.961 (0.807)	8.236 (0.78)	8.688 (0.838)
**PADI1**	7.608 (1.479)	7.556 (0.95)	7.531 (0.593)	7.67 (0.864)
**PLA2G2A**	13.128 (1.914)	12.21 (2.306)	12.628 (1.994)	12.166 (2.002)
**PRSS2**	13.702 (1.815)	12.944 (1.596)	12.762 (1.823)	12.049 (1.496)

Supplementary Table 16: Sub-group summary statistics of top differentially expressed proteins.
Summary statistics (Mean and standard deviation) of the top10 or bottom 10 expressed proteins. Proteins were selected after assessment of log2 fold change of expression between LPS^{hi} F sub-group (n=39) and community children (n=251). Statistics are presented for each LPS-mortality sub-group (LPS^{lo} F, LPS^{hi} NF, LPS^{lo}NF and LPS^{hi} F). Two-tailed Wilcoxon p values are provided in Supplementary Table 17. All n values refer to independent children; no technical replicates were used. SD = standard deviation, F = Fatal, NF = Non-fatal.

	GENE	COMPARISON	P_VALUE
**1**	CA6.1	LPShi F vs LPSlo F	0.122
**2**	CA6.1	LPShi F vs LPShi NF	5.65E-05
**3**	CA6.1	LPShi F vs LPSlo NF	1.43E-07
**4**	IGF1	LPShi F vs LPSlo F	0.0381
**5**	IGF1	LPShi F vs LPShi NF	1.99E-06
**6**	IGF1	LPShi F vs LPSlo NF	<1e-10
**7**	IGF2	LPShi F vs LPSlo F	0.0166
**8**	IGF2	LPShi F vs LPShi NF	6.20E-08
**9**	IGF2	LPShi F vs LPSlo NF	<1e-10
**10**	LTA.LTB	LPShi F vs LPSlo F	2.66E-05
**11**	LTA.LTB	LPShi F vs LPShi NF	1.56E-07
**12**	LTA.LTB	LPShi F vs LPSlo NF	<1e-10
**13**	CA6	LPShi F vs LPSlo F	0.214
**14**	CA6	LPShi F vs LPShi NF	0.000493
**15**	CA6	LPShi F vs LPSlo NF	9.70E-07
**16**	COL10A1	LPShi F vs LPSlo F	0.502
**17**	COL10A1	LPShi F vs LPShi NF	0.000131
**18**	COL10A1	LPShi F vs LPSlo NF	5.15E-09
**19**	IGLL1	LPShi F vs LPSlo F	0.808
**20**	IGLL1	LPShi F vs LPShi NF	0.000826
**21**	IGLL1	LPShi F vs LPSlo NF	1.32E-07
**22**	CDON	LPShi F vs LPSlo F	0.00135
**23**	CDON	LPShi F vs LPShi NF	0.00131
**24**	CDON	LPShi F vs LPSlo NF	5.56E-09
**25**	IGFBP3	LPShi F vs LPSlo F	0.14
**26**	IGFBP3	LPShi F vs LPShi NF	5.49E-06
**27**	IGFBP3	LPShi F vs LPSlo NF	<1e-10
**28**	COL1A1	LPShi F vs LPSlo F	0.233
**29**	COL1A1	LPShi F vs LPShi NF	0.000107
**30**	COL1A1	LPShi F vs LPSlo NF	5.47E-08
**31**	IL1RL1	LPShi F vs LPSlo F	0.299
**32**	IL1RL1	LPShi F vs LPShi NF	2.77E-05
**33**	IL1RL1	LPShi F vs LPSlo NF	2.17E-09
**34**	PRSS2	LPShi F vs LPSlo F	0.0189
**35**	PRSS2	LPShi F vs LPShi NF	0.0191
**36**	PRSS2	LPShi F vs LPSlo NF	7.28E-08
**37**	PADI1	LPShi F vs LPSlo F	0.565
**38**	PADI1	LPShi F vs LPShi NF	0.42
**39**	PADI1	LPShi F vs LPSlo NF	0.0174

**40**	DEFA3	LPShi F vs LPSlo F	0.00548
**41**	DEFA3	LPShi F vs LPShi NF	7.98E-06
**42**	DEFA3	LPShi F vs LPSlo NF	<1e-10
**43**	HAMP	LPShi F vs LPSlo F	0.0353
**44**	HAMP	LPShi F vs LPShi NF	0.0157
**45**	HAMP	LPShi F vs LPSlo NF	0.000775
**46**	PLA2G2A	LPShi F vs LPSlo F	0.0221
**47**	PLA2G2A	LPShi F vs LPShi NF	0.227
**48**	PLA2G2A	LPShi F vs LPSlo NF	0.0039
**49**	FTL	LPShi F vs LPSlo F	0.892
**50**	FTL	LPShi F vs LPShi NF	0.00529
**51**	FTL	LPShi F vs LPSlo NF	0.000826
**52**	FTH1.FTL	LPShi F vs LPSlo F	0.951
**53**	FTH1.FTL	LPShi F vs LPShi NF	0.00499
**54**	FTH1.FTL	LPShi F vs LPSlo NF	0.00116
**55**	IL1RN	LPShi F vs LPSlo F	0.00108
**56**	IL1RN	LPShi F vs LPShi NF	2.54E-05
**57**	IL1RN	LPShi F vs LPSlo NF	2.27E-08
**58**	LCN2	LPShi F vs LPSlo F	0.0153
**59**	LCN2	LPShi F vs LPShi NF	0.00458
**60**	LCN2	LPShi F vs LPSlo NF	5.77E-10

Supplementary Table 17: Wilcoxon sub-group comparison of top differentially expressed proteins.
Two-tailed Wilcoxon comparison of LPS-mortality sub-groups (LPS^{lo} F, LPS^{hi} NF LPS^{lo} NF vs. LPS^{hi} F) of the top10 or bottom 10 expressed proteins in the LPS^{hi} F sub-group. The top 10 and bottom 10 proteins were selected after assessment of log2 fold change of expression between LPS^{hi} F sub-group (n=39) and community children (n=251). All n values refer to independent children; no technical replicates were used. F = Fatal, NF = Non-fatal.

LPS-mortality sub-group	emmean	SE	df	lower.CL	upper.CL	Protein
LPShi Fatal	1173.077	384.5722	619	417.8524	1928.301	CA6
LPShi Non-Fatal	1938.974	375.339	619	1201.882	2676.066	CA6
LPSlo Fatal	1484.242	290.3392	619	914.0728	2054.411	CA6
LPSlo Non-Fatal	2140.859	265.684	619	1619.108	2662.61	CA6
LPShi Fatal	864.1435	374.1236	619	129.4382	1598.849	CA6.1
LPShi Non-Fatal	2045.688	559.8362	619	946.28	3145.097	CA6.1
LPSlo Fatal	1274.287	329.0993	619	628.0009	1920.574	CA6.1
LPSlo Non-Fatal	1969.06	319.4682	619	1341.687	2596.432	CA6.1
LPShi Fatal	5588.272	1110.139	619	3408.178	7768.367	CDON
LPShi Non-Fatal	7909.94	980.8855	619	5983.673	9836.207	CDON
LPSlo Fatal	7441.527	871.4385	619	5730.193	9152.861	CDON
LPSlo Non-Fatal	9970.114	919.8805	619	8163.649	11776.58	CDON
LPShi Fatal	5492.62	1493.161	619	2560.345	8424.896	COL10A1
LPShi Non-Fatal	9955.818	1441.856	619	7124.295	12787.34	COL10A1
LPSlo Fatal	7440.063	1346.98	619	4794.859	10085.27	COL10A1
LPSlo Non-Fatal	12645.06	976.8413	619	10726.73	14563.38	COL10A1
LPShi Fatal	16950.25	4233.084	619	8637.302	25263.2	COL1A1
LPShi Non-Fatal	30220.29	6032.217	619	18374.2	42066.38	COL1A1
LPSlo Fatal	20558.81	3617.997	619	13453.78	27663.85	COL1A1
LPSlo Non-Fatal	30359.81	3627.712	619	23235.7	37483.93	COL1A1
LPShi Fatal	49670.95	6942.484	619	36037.27	63304.63	DEFA3
LPShi Non-Fatal	22197.47	4107.251	619	14131.63	30263.3	DEFA3
LPSlo Fatal	33616.29	3779.117	619	26194.85	41037.74	DEFA3
LPSlo Non-Fatal	22571.43	2603.268	619	17459.12	27683.74	DEFA3
LPShi Fatal	21609.36	4977.939	619	11833.66	31385.05	FTH1.FTL
LPShi Non-Fatal	10404.63	3339.21	619	3847.077	16962.18	FTH1.FTL
LPSlo Fatal	22574.03	3238.901	619	16213.47	28934.6	FTH1.FTL
LPSlo Non-Fatal	15136.4	1887.537	619	11429.65	18843.15	FTH1.FTL
LPShi Fatal	23628.4	5370.468	619	13081.85	34174.94	FTL
LPShi Non-Fatal	11493.15	3669.202	619	4287.554	18698.74	FTL
LPSlo Fatal	24489.76	3553.198	619	17511.97	31467.54	FTL
LPSlo Non-Fatal	16396.71	2070.689	619	12330.28	20463.14	FTL
LPShi Fatal	14850.95	2623.234	619	9699.436	20002.47	HAMP
LPShi Non-Fatal	8848.527	1820.132	619	5274.145	12422.91	HAMP
LPSlo Fatal	9626.466	1359.883	619	6955.923	12297.01	HAMP
LPSlo Non-Fatal	8305.459	1046.816	619	6249.718	10361.2	HAMP
LPShi Fatal	2860.118	628.3014	619	1626.257	4093.978	IGF1
LPShi Non-Fatal	4400.988	511.0229	619	3397.439	5404.536	IGF1
LPSlo Fatal	3924.229	406.5388	619	3125.866	4722.591	IGF1
LPSlo Non-Fatal	5189.633	351.7097	619	4498.944	5880.322	IGF1
LPShi Fatal	2595.71	936.1409	619	757.3125	4434.107	IGF2
LPShi Non-Fatal	5998.152	869.6949	619	4290.242	7706.063	IGF2
LPSlo Fatal	4180.702	563.977	619	3073.162	5288.242	IGF2
LPSlo Non-Fatal	7100.074	361.9855	619	6389.206	7810.943	IGF2
LPShi Fatal	6678.33	1320.518	619	4085.093	9271.568	IGFBP3
LPShi Non-Fatal	9649.006	863.712	619	7952.845	11345.17	IGFBP3
LPSlo Fatal	7393.48	621.7589	619	6172.467	8614.492	IGFBP3
LPSlo Non-Fatal	10360.89	503.7359	619	9371.648	11350.12	IGFBP3
LPShi Fatal	6546.635	1626.629	619	3352.256	9741.014	IGLL1
LPShi Non-Fatal	12699.33	2133.669	619	8509.226	16889.44	IGLL1
LPSlo Fatal	6593.671	1109.575	619	4414.683	8772.659	IGLL1
LPSlo Non-Fatal	12471.88	994.7751	619	10518.33	14425.42	IGLL1
LPShi Fatal	47216.12	8144.892	619	31221.15	63211.08	IL1RL1
LPShi Non-Fatal	21394.37	3872.965	619	13788.63	29000.12	IL1RL1
LPSlo Fatal	43796.92	4283.198	619	35385.56	52208.28	IL1RL1
LPSlo Non-Fatal	22785.96	2414.964	619	18043.45	27528.48	IL1RL1
LPShi Fatal	16868.22	2411.991	619	12131.54	21604.89	IL1RN
LPShi Non-Fatal	7325.93	1672.591	619	4041.289	10610.57	IL1RN
LPSlo Fatal	10386.17	948.6918	619	8523.122	12249.21	IL1RN

LPS-mortality sub-group	emmean	SE	df	lower.CL	upper.CL	Protein
LPSlo Non-Fatal	7375.103	553.0968	619	6288.929	8461.276	IL1RN
LPShi Fatal	4784.382	906.6958	619	3003.809	6564.954	LCN2
LPShi Non-Fatal	2133.481	319.9961	619	1505.072	2761.891	LCN2
LPSlo Fatal	3171.805	412.648	619	2361.445	3982.164	LCN2
LPSlo Non-Fatal	1780.403	216.8536	619	1354.545	2206.261	LCN2
LPShi Fatal	183.2128	33.06063	619	118.2882	248.1374	LTA.LTB
LPShi Non-Fatal	324.5653	32.94165	619	259.8744	389.2562	LTA.LTB
LPSlo Fatal	258.6172	22.75383	619	213.9332	303.3013	LTA.LTB
LPSlo Non-Fatal	406.5784	19.45475	619	368.3731	444.7838	LTA.LTB
LPShi Fatal	1694.57	1497.267	619	-1245.77	4634.909	PADI1
LPShi Non-Fatal	160.244	92.11748	619	-20.6567	341.1446	PADI1
LPSlo Fatal	377.8853	271.6216	619	-155.526	911.2967	PADI1
LPSlo Non-Fatal	304.2167	105.028	619	97.96225	510.4711	PADI1
LPShi Fatal	21138.19	4156.745	619	12975.16	29301.23	PLA2G2A
LPShi Non-Fatal	15149.91	4392.517	619	6523.871	23775.95	PLA2G2A
LPSlo Fatal	19673.05	2905.019	619	13968.16	25377.93	PLA2G2A
LPSlo Non-Fatal	14075.04	2163.645	619	9826.063	18324.01	PLA2G2A
LPShi Fatal	24650.9	5369.544	619	14106.16	35195.63	PRSS2
LPShi Non-Fatal	17810.19	3618.508	619	10704.15	24916.23	PRSS2
LPSlo Fatal	15599.04	2790.489	619	10119.06	21079.01	PRSS2
LPSlo Non-Fatal	11329.41	2062.808	619	7278.457	15380.36	PRSS2

Supplementary Table 18 – Predicted Means based on top and bottom differentially expressed proteins in the *LPS^{hi} F* sub-group

Survey-weighted generalized linear models were used to estimate the adjusted predicted mean abundance (± standard error) for the 4 LPS-survival status sub-groups (LPS^{hi} Fatal, LPS^{hi} Non-Fatal, LPS^{lo} Fatal, LPS^{lo} Non-Fatal), based on the 10 top and 10 bottom differentially expressed proteins in the LPS^{hi} F sub-group (n=39). Models were adjusted for age, sex, HIV status, and study site. Values represent marginal means with standard errors generated using the emmeans package. Pairwise contrasts were tested using two-tailed comparisons, with Holm adjustment applied for multiple testing. All n values refer to independent children; no technical replicates were used.

Comparison	estimate	SE	df	t.ratio	p.value	Protein
LPShi Fatal - LPShi Non-Fatal	-765.897	427.9397	619	-1.78973	0.295944	CA6
LPShi Fatal - LPSlo Fatal	-311.165	378.6409	619	-0.82179	0.82302	CA6
LPShi Fatal - LPSlo Non-Fatal	-967.782	353.6853	619	-2.73628	0.038353	CA6
LPShi Non-Fatal - LPSlo Fatal	454.7317	363.3839	619	1.251381	0.633805	CA6
LPShi Non-Fatal - LPSlo Non-Fatal	-201.885	330.4008	619	-0.61103	0.82302	CA6
LPSlo Fatal - LPSlo Non-Fatal	-656.617	262.5721	619	-2.50071	0.063259	CA6
LPShi Fatal - LPShi Non-Fatal	-1181.54	467.7285	619	-2.52613	0.047123	CA6.1
LPShi Fatal - LPSlo Fatal	-410.144	325.7859	619	-1.25894	0.417055	CA6.1
LPShi Fatal - LPSlo Non-Fatal	-1104.92	306.7209	619	-3.60235	0.002044	CA6.1
LPShi Non-Fatal - LPSlo Fatal	771.4011	445.0984	619	1.733102	0.250726	CA6.1
LPShi Non-Fatal - LPSlo Non-Fatal	76.62893	409.1866	619	0.187271	0.851509	CA6.1
LPSlo Fatal - LPSlo Non-Fatal	-694.772	259.9284	619	-2.67294	0.038586	CA6.1
LPShi Fatal - LPShi Non-Fatal	-2321.67	1011.482	619	-2.29531	0.066146	CDON
LPShi Fatal - LPSlo Fatal	-1853.25	998.8381	619	-1.85541	0.128026	CDON
LPShi Fatal - LPSlo Non-Fatal	-4381.84	958.3925	619	-4.57207	3.50E-05	CDON
LPShi Non-Fatal - LPSlo Fatal	468.4128	815.8649	619	0.57413	0.566088	CDON
LPShi Non-Fatal - LPSlo Non-Fatal	-2060.17	672.9927	619	-3.06121	0.0092	CDON
LPSlo Fatal - LPSlo Non-Fatal	-2528.59	685.2293	619	-3.69013	0.001219	CDON
LPShi Fatal - LPShi Non-Fatal	-4463.2	1784.612	619	-2.50093	0.050576	COL10A1
LPShi Fatal - LPSlo Fatal	-1947.44	1712.11	619	-1.13745	0.296269	COL10A1
LPShi Fatal - LPSlo Non-Fatal	-7152.44	1457.104	619	-4.90867	7.05E-06	COL10A1
LPShi Non-Fatal - LPSlo Fatal	2515.755	1737.448	619	1.44796	0.296269	COL10A1
LPShi Non-Fatal - LPSlo Non-Fatal	-2689.24	1358.313	619	-1.97984	0.144492	COL10A1
LPSlo Fatal - LPSlo Non-Fatal	-5205	1371.802	619	-3.79428	0.000813	COL10A1
LPShi Fatal - LPShi Non-Fatal	-13270	6156.796	619	-2.15535	0.126077	COL1A1
LPShi Fatal - LPSlo Fatal	-3608.56	4004.296	619	-0.90117	0.735693	COL1A1
LPShi Fatal - LPSlo Non-Fatal	-13409.6	3738.996	619	-3.58641	0.00217	COL1A1
LPShi Non-Fatal - LPSlo Fatal	9661.48	5813.069	619	1.662027	0.291041	COL1A1
LPShi Non-Fatal - LPSlo Non-Fatal	-139.522	5706.311	619	-0.02445	0.980501	COL1A1
LPSlo Fatal - LPSlo Non-Fatal	-9801	3180.034	619	-3.08204	0.010736	COL1A1
LPShi Fatal - LPShi Non-Fatal	27473.48	7668.961	619	3.582425	0.001836	DEFA3
LPShi Fatal - LPSlo Fatal	16054.66	7336.817	619	2.188232	0.058052	DEFA3
LPShi Fatal - LPSlo Non-Fatal	27099.52	7029.647	619	3.855032	0.000767	DEFA3
LPShi Non-Fatal - LPSlo Fatal	-11418.8	4719.612	619	-2.41944	0.047495	DEFA3
LPShi Non-Fatal - LPSlo Non-Fatal	-373.963	3891.844	619	-0.09609	0.923481	DEFA3
LPSlo Fatal - LPSlo Non-Fatal	11044.86	3371.655	619	3.275798	0.00445	DEFA3
LPShi Fatal - LPShi Non-Fatal	11204.73	5341.904	619	2.097516	0.145412	FTH1.FTL
LPShi Fatal - LPSlo Fatal	-964.674	5388.606	619	-0.17902	0.85798	FTH1.FTL
LPShi Fatal - LPSlo Non-Fatal	6472.96	4748.245	619	1.363232	0.37387	FTH1.FTL
LPShi Non-Fatal - LPSlo Fatal	-12169.4	4251.46	619	-2.86241	0.026079	FTH1.FTL
LPShi Non-Fatal - LPSlo Non-Fatal	-4731.77	3077.095	619	-1.53774	0.37387	FTH1.FTL
LPSlo Fatal - LPSlo Non-Fatal	7437.635	3329.514	619	2.23385	0.129247	FTH1.FTL
LPShi Fatal - LPShi Non-Fatal	12135.25	5810.05	619	2.088666	0.148583	FTL
LPShi Fatal - LPSlo Fatal	-861.36	5865.226	619	-0.14686	0.883291	FTL
LPShi Fatal - LPSlo Non-Fatal	7231.69	5169.562	619	1.398898	0.430849	FTL
LPShi Non-Fatal - LPSlo Fatal	-12996.6	4640.768	619	-2.80053	0.031567	FTL
LPShi Non-Fatal - LPSlo Non-Fatal	-4903.56	3348.712	619	-1.46431	0.430849	FTL
LPSlo Fatal - LPSlo Non-Fatal	8093.05	3639.982	619	2.223376	0.132753	FTL
LPShi Fatal - LPShi Non-Fatal	6002.427	2887.711	619	2.07861	0.190323	HAMP
LPShi Fatal - LPSlo Fatal	5224.487	2615.885	619	1.997216	0.190323	HAMP
LPShi Fatal - LPSlo Non-Fatal	6545.494	2485.479	619	2.633494	0.051974	HAMP
LPShi Non-Fatal - LPSlo Fatal	-777.939	1760.366	619	-0.44192	1	HAMP
LPShi Non-Fatal - LPSlo Non-Fatal	543.0674	1684.277	619	0.322433	1	HAMP

Comparison	estimate	SE	df	t.ratio	p.value	Protein
LPSlo Fatal - LPSlo Non-Fatal	1321.007	979.6751	619	1.348413	0.534056	HAMP
LPShi Fatal - LPShi Non-Fatal	-1540.87	698.3136	619	-2.20656	0.110845	IGF1
LPShi Fatal - LPSlo Fatal	-1064.11	642.1102	619	-1.65721	0.293952	IGF1
LPShi Fatal - LPSlo Non-Fatal	-2329.52	626.2617	619	-3.71972	0.001305	IGF1
LPShi Non-Fatal - LPSlo Fatal	476.7591	546.2854	619	0.872729	0.383149	IGF1
LPShi Non-Fatal - LPSlo Non-Fatal	-788.645	502.9307	619	-1.5681	0.293952	IGF1
LPSlo Fatal - LPSlo Non-Fatal	-1265.4	382.67	619	-3.30678	0.004992	IGF1
LPShi Fatal - LPShi Non-Fatal	-3402.44	1190.835	619	-2.85719	0.017671	IGF2
LPShi Fatal - LPSlo Fatal	-1584.99	1001.859	619	-1.58205	0.228297	IGF2
LPShi Fatal - LPSlo Non-Fatal	-4504.36	970.5969	619	-4.64082	2.12E-05	IGF2
LPShi Non-Fatal - LPSlo Fatal	1817.45	967.893	619	1.877739	0.18266	IGF2
LPShi Non-Fatal - LPSlo Non-Fatal	-1101.92	934.3988	619	-1.17928	0.238738	IGF2
LPSlo Fatal - LPSlo Non-Fatal	-2919.37	597.6206	619	-4.88499	7.91E-06	IGF2
LPShi Fatal - LPShi Non-Fatal	-2970.68	1489.685	619	-1.99416	0.139717	IGFBP3
LPShi Fatal - LPSlo Fatal	-715.15	1360.749	619	-0.52556	0.842869	IGFBP3
LPShi Fatal - LPSlo Non-Fatal	-3682.56	1354.558	619	-2.71864	0.033694	IGFBP3
LPShi Non-Fatal - LPSlo Fatal	2255.526	944.8953	619	2.387065	0.069131	IGFBP3
LPShi Non-Fatal - LPSlo Non-Fatal	-711.88	884.9054	619	-0.80447	0.842869	IGFBP3
LPSlo Fatal - LPSlo Non-Fatal	-2967.41	615.9316	619	-4.81775	1.10E-05	IGFBP3
LPShi Fatal - LPShi Non-Fatal	-6152.7	2388.309	619	-2.57617	0.030662	IGLL1
LPShi Fatal - LPSlo Fatal	-47.0362	1609.823	619	-0.02922	1	IGLL1
LPShi Fatal - LPSlo Non-Fatal	-5925.24	1614.258	619	-3.67057	0.001315	IGLL1
LPShi Non-Fatal - LPSlo Fatal	6105.661	2108.426	619	2.895839	0.01566	IGLL1
LPShi Non-Fatal - LPSlo Non-Fatal	227.4568	2152.608	619	0.105666	1	IGLL1
LPSlo Fatal - LPSlo Non-Fatal	-5878.2	1089.095	619	-5.39733	5.79E-07	IGLL1
LPShi Fatal - LPShi Non-Fatal	25821.74	8731.202	619	2.957409	0.012883	IL1RL1
LPShi Fatal - LPSlo Fatal	3419.196	8684.349	619	0.393719	1	IL1RL1
LPShi Fatal - LPSlo Non-Fatal	24430.15	8263.135	619	2.956523	0.012883	IL1RL1
LPShi Non-Fatal - LPSlo Fatal	-22402.5	5037.014	619	-4.44758	5.15E-05	IL1RL1
LPShi Non-Fatal - LPSlo Non-Fatal	-1391.59	3656.011	619	-0.38063	1	IL1RL1
LPSlo Fatal - LPSlo Non-Fatal	21010.95	3856.35	619	5.448404	4.41E-07	IL1RL1
LPShi Fatal - LPShi Non-Fatal	9542.286	2905.069	619	3.284701	0.005393	IL1RN
LPShi Fatal - LPSlo Fatal	6482.05	2548.306	619	2.543671	0.033634	IL1RN
LPShi Fatal - LPSlo Non-Fatal	9493.113	2476.269	619	3.833635	0.000835	IL1RN
LPShi Non-Fatal - LPSlo Fatal	-3060.24	1812.534	619	-1.68837	0.183686	IL1RN
LPShi Non-Fatal - LPSlo Non-Fatal	-49.1723	1792.07	619	-0.02744	0.978119	IL1RN
LPSlo Fatal - LPSlo Non-Fatal	3011.064	995.4535	619	3.024816	0.010364	IL1RN
LPShi Fatal - LPShi Non-Fatal	2650.901	949.4051	619	2.79217	0.021589	LCN2
LPShi Fatal - LPSlo Fatal	1612.577	979.8305	619	1.645772	0.200637	LCN2
LPShi Fatal - LPSlo Non-Fatal	3003.979	916.7784	619	3.276668	0.005546	LCN2
LPShi Non-Fatal - LPSlo Fatal	-1038.32	441.6044	619	-2.35125	0.057069	LCN2
LPShi Non-Fatal - LPSlo Non-Fatal	353.0781	264.3608	619	1.335592	0.200637	LCN2
LPSlo Fatal - LPSlo Non-Fatal	1391.402	362.019	619	3.84345	0.000803	LCN2
LPShi Fatal - LPShi Non-Fatal	-141.353	40.94616	619	-3.45216	0.002376	LTA.LTB
LPShi Fatal - LPSlo Fatal	-75.4045	33.60488	619	-2.24385	0.050389	LTA.LTB
LPShi Fatal - LPSlo Non-Fatal	-223.366	33.25885	619	-6.71598	2.54E-10	LTA.LTB
LPShi Non-Fatal - LPSlo Fatal	65.94807	34.66325	619	1.902536	0.057565	LTA.LTB
LPShi Non-Fatal - LPSlo Non-Fatal	-82.0131	32.36658	619	-2.53388	0.034578	LTA.LTB
LPSlo Fatal - LPSlo Non-Fatal	-147.961	22.73511	619	-6.50805	7.87E-10	LTA.LTB
LPShi Fatal - LPShi Non-Fatal	1534.326	1519.557	619	1.009719	1	PADI1
LPShi Fatal - LPSlo Fatal	1316.685	1566.415	619	0.840572	1	PADI1
LPShi Fatal - LPSlo Non-Fatal	1390.354	1541.334	619	0.902046	1	PADI1
LPShi Non-Fatal - LPSlo Fatal	-217.641	284.8844	619	-0.76396	1	PADI1

Comparison	estimate	SE	df	t.ratio	p.value	Protein
LPShi Non-Fatal - LPSlo Non-Fatal	-143.973	127.8916	619	-1.12574	1	PADI1
LPSlo Fatal - LPSlo Non-Fatal	73.6686	325.589	619	0.226263	1	PADI1
LPShi Fatal - LPShi Non-Fatal	5988.282	5422.834	619	1.104272	1	PLA2G2A
LPShi Fatal - LPSlo Fatal	1465.146	4398.532	619	0.333099	1	PLA2G2A
LPShi Fatal - LPSlo Non-Fatal	7063.157	3928.649	619	1.797859	0.363433	PLA2G2A
LPShi Non-Fatal - LPSlo Fatal	-4523.14	4603.148	619	-0.98262	1	PLA2G2A
LPShi Non-Fatal - LPSlo Non-Fatal	1074.875	4374.154	619	0.245733	1	PLA2G2A
LPSlo Fatal - LPSlo Non-Fatal	5598.011	2656.638	619	2.107178	0.213023	PLA2G2A
LPShi Fatal - LPShi Non-Fatal	6840.707	6078.923	619	1.125316	0.521783	PRSS2
LPShi Fatal - LPSlo Fatal	9051.858	5477.332	619	1.652604	0.296756	PRSS2
LPShi Fatal - LPSlo Non-Fatal	13321.49	5294.222	619	2.516231	0.072681	PRSS2
LPShi Non-Fatal - LPSlo Fatal	2211.15	3703.335	619	0.59707	0.550679	PRSS2
LPShi Non-Fatal - LPSlo Non-Fatal	6480.78	3358.189	619	1.929844	0.223424	PRSS2
LPSlo Fatal - LPSlo Non-Fatal	4269.63	2122.403	619	2.011697	0.223424	PRSS2

Supplementary Table 19 – Pairwise comparisons based on top and bottom differentially expressed proteins in the *LPS^{hi} F* sub-group

Two-tailed pairwise comparisons of model-adjusted means were generated between LPS-survival sub-groups for the top 10 and bottom 10 biomarkers identified from the LPS^{hi} F sub-group (n=39). P-values were calculated using the emmeans package with appropriate multiplicity adjustments and are derived from regression models adjusted for age, sex, HIV status, and site. These values indicate the significance of differences in adjusted means between sub-groups. All n values refer to independent children; no technical replicates were used.

Biomarker	p_value
EAEC	0.001705
CTX_M	0.019798
mphA_Azithromysin.resistance	0.004716
X16s	5.05E-04
Rotavirus	0.11791
Fecal Calprotectin	0.007936
HP	0.047805
AOC1	0.704063
Plasma MPO	3.08E-05
CD14.1	7.80E-17
LBP	0.009014
LY6G6C	6.25E-13
TREM1	1.63E-08
CDH1.1	0.193468
CDH1.2	4.93E-04
OCLN	0.008189
TLR4	0.335772
TJP1	0.201287
TNFAIP3	0.66918
GOLM2	2.67E-06
SELPLG	0.304116
ADH5	1.29E-05
DNAJB2	4.93E-05
TRAF4	0.007926
CD7	0.208954
NRBP1	0.472894
KCNG4	0.016761
CD247	0.417098
S100A16	0.652134
SMS	0.377421
DCTN6	0.443573
C1QL3	5.35E-04
ATXN10	0.445961
ICOSLG	0.985651
SIRPB1	0.970759
CLEC6A	0.009268
SELPLG.1	0.360717
LILRA5.1	0.225547

Supplementary Table 20 – Univariate Cox Proportional Hazards Models for Biomarkers and Mortality
Univariate Cox proportional hazards models were used to evaluate associations between 38 individual biomarkers and mortality among children from the admission cohort (n=635). Biomarkers selection criteria: (1) 19 based on biological relevance and differential expression in adjusted or unadjusted comparisons (see Supplementary Tables 16-19), and (2) 19 based on their strongest correlations with plasma LPS in the LPS^{hi} F subgroup (Figure 3 & Supplementary Table 8). The table presents two-tailed p-values for each biomarker. Inverse probability weighting was applied to account for selection bias. Biomarkers with $p < 0.2$ were highlighted in green to indicate potential inclusion in the multivariable model.
LPS = lipopolysaccharide, MPO = myeloperoxidase, AAT = alpha-1-antitrypsin, mCD14 = membrane CD14.

Variable	HR[1]	95% CI[1]	p-value
Plasma LPS	1.39	1.06, 1.82	0.019
Anthropometric classification	0.54	0.40, 0.72	<0.001
Fecal Calprotectin	1.26	1.09, 1.47	0.002
Plasma MPO	1.37	1.08, 1.75	0.010
CD14.1	1.48	1.28, 1.72	<0.001
TREM1	1.28	1.08, 1.53	0.005
CLEC6A	1.23	1.05, 1.43	0.011
TRAF4	1.28	1.05, 1.56	0.015
EAEC	1.12	0.86, 1.45	0.4
CTX_M	0.78	0.58, 1.06	0.11
mphA_Azithromycin.resistance	0.77	0.53, 1.10	0.15
X16s	0.93	0.65, 1.31	0.7
Rotavirus	0.85	0.60, 1.19	0.3
HP	1.01	0.78, 1.30	>0.9
LBP	1.03	0.80, 1.33	0.8
LY6G6C	1.09	0.81, 1.47	0.6
CDH1.1	0.73	0.46, 1.17	0.2
CDH1.2	0.83	0.58, 1.17	0.3
OCLN	1.10	0.94, 1.29	0.2
GOLM2	0.77	0.51, 1.16	0.2
ADH5	1.05	0.79, 1.40	0.7
DNAJB2	1.03	0.79, 1.35	0.8
KCNG4	0.81	0.58, 1.12	0.2
C1QL3	0.94	0.65, 1.35	0.7
Admission age	0.79	0.60, 1.04	0.089
Sex	1.42	1.12, 1.80	0.004
HIV status	1.06	0.86, 1.30	0.6
Site	1.00	1.00, 1.00	0.3

[1]HR = Hazard Ratio, CI = Confidence Interval

Supplementary Table 21 – Full Multivariable CoxPH model including 22 Biomarkers and mortality-related covariates
A multivariable Cox proportional hazards model was fitted to assess independent associations between biomarker levels and mortality among children admitted to hospital (n=635). The model included 22 biomarkers identified through univariate screening (p < 0.2) and selection criteria (see Supplementary Table 20), along with known mortality-related factors (anthropometric classification, HIV status, study site) and, age, and sex (as defined in Figure 2D). All covariates were entered simultaneously and mutually adjusted. Hazard ratios (HRs), 95% confidence intervals, confidence interval and two-tailed p-values are reported. All covariates are tested simultaneously within a single model. Inverse probability weighting was applied to account for selection bias. This full model was used to identify significant predictors for final visualization (see Figure 2E). LPS = lipopolysaccharide, MPO = myeloperoxidase, AAT = alpha-1-antitrypsin, mCD14 = membrane CD14.

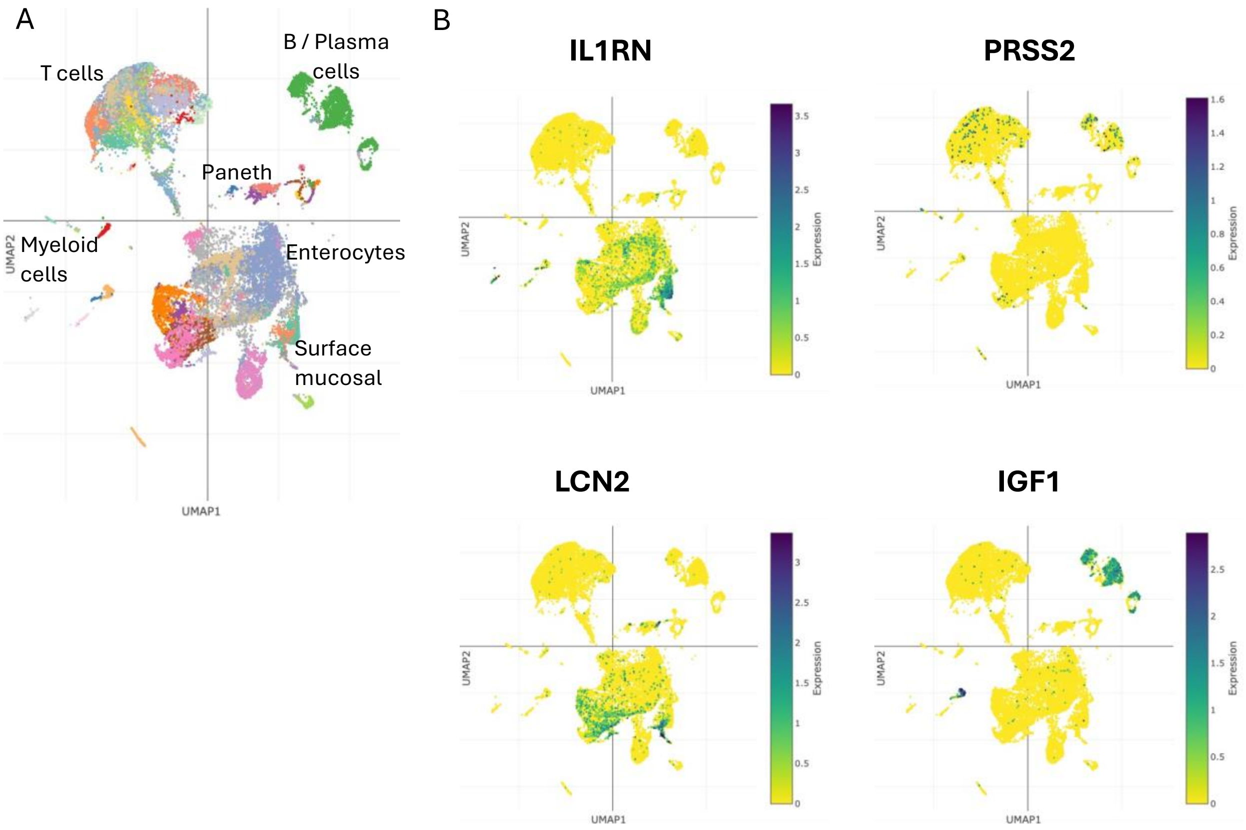

Supplementary Figure 7: Deconvolution of SomaScan proteomics data on single-cell transcriptomics dataset of enteropathy.[9]

The differentially expressed proteins in LPS^hi Fatal cohort (n=39) were mapped onto the single cell transcriptomics dataset. (A) UMAP of enteropathy single cells data identifying cell types including immune cells (myeloid cells, T cell, B cell, plasma cell) and enterocytes (surface mucosal cell, Paneth cell, enterocytes, etc.) (B) UMAP embedding shows expression of representative genes of proteins differentially expressed proteins in LPS^hi Fatal cohort.

PROTEIN	IMMUNITY	NUTRITION	TISSUE REPAIR / REGENERATION	LPS SIGNALING
**SELPLG**	Yes (leukocyte adhesion, T-cell trafficking)	No	No	Yes (upregulated in LPS challenge)
**ADH5**	No	Yes (formaldehyde & NO metabolism, oxidative stress protection)	No	Yes (protects against nitrosative stress from LPS)
**DNAJB2**	No	No	Yes (molecular chaperone in neuronal repair, stress recovery)	No
**TRAF4**	Yes (TNF receptor signaling, T-cell costimulation)	No	No	Yes (modulates TLR4 pathway downstream)
**CD7**	Yes (T-cell activation and signaling)	No	No	No
**CD247**	Yes (part of CD3-TCR complex)	No	No	No
**S100A16**	Yes (monocyte chemotaxis, inflammatory signaling)	No	No	No
**SMS**	No	Yes (polyamine biosynthesis—linked to cell growth and nutritional status)	No	No
**ICOSLG**	Yes (costimulation of T cells)	No	No	No
**CLEC6A**	Yes (dectin-2 receptor, antifungal immunity)	No	No	Yes (triggers NF-κB via SYK, can be upregulated with LPS)
**LILRA5**	Yes (regulation of myeloid immune cell activity)	No	No	No

Supplementary Table 22: Function of top LPS-correlated proteins in the *LPS^hi^ Fatal* sub-group.
11 of the 18 proteins most correlated with plasma LPS in the LPS^{hi} Fatal sub-group has function in immunity, nutrition, tissue repair and/or LPS signalling.

Gene	Description	GO_Term	EntrezGene_ID	HGNC_ID	GO_ID
1 ADH5	alcohol dehydrogenase 5 (class III), chi polypeptide	zinc ion binding; oxidoreductase activity; S-(hydroxymethyl)glutathione dehydrogenase (NAD(P)+) activity; formaldehyde catabolic process; lipid metabolic process; metal ion binding; small molecule metabolic process; fatty acid binding; response to redox state; electron transfer activity; alcohol dehydrogenase (NAD+) activity, fatty acid omega-oxidation; formaldehyde dehydrogenase activity; retinoid metabolic process; respiratory system process; **response to lipopolysaccharide**; positive regulation of blood pressure; response to nitrosative stress	128	HGNC:253	0008270; 0016491; 0051903; 0046294; 0005737; 0006629; 0046872; 0044281; 0005829; 0070062; 0005504; 0051775; 0009055; 0004022; 0004024; 0010430; 0018467; 0042802; 0106321; 0106322; 0001523; 0003016; 0032496; 0045777; 0051409; 0005739
2 ATXN10	ataxin 10	Cell division; cytoskeleton; nervous system development; dendrite; centriole; midbody; ciliary basal body; neuron projection development; **regulation of cytokinesis**; neuronal cell body; cilium assembly	25814	HGNC:10549	0005737; 0016020; 0042995; 0005829; 0051301; 0005886; 0005856; 0005515; 0048471; 0007399; 0005615; 0030425; 0005814; 0030496; 0036064; 0031175; 0032465; 0043025; 0060271
3 C1QL3	complement C1q like 3	Regulation of synapse organization; postsynaptic density assembly; neurotransmitter receptor localization to postsynaptic specialization membrane; synaptic cleft; hippocampal mossy fiber to CA3 synapse; glutamatergic synapse	389941	HGNC:19359	0005515; 0005576; 0005581; 0042802; 0050807; 0097107; 0099645; 0043083; 0098686; 0098978
4 CD247	CD247 molecule	Cell surface receptor signaling pathway; transmembrane signaling receptor activity; plasma membrane protein complex; protein binding; **adaptive immune response**; alpha-beta T cell receptor complex and activation; T cell receptor signaling pathway; protein homodimerization activity; protein heterodimerization activity; Fc-gamma receptor signaling pathway; **gamma-delta T cell activation**; gamma-delta T cell receptor complex; Fc-gamma receptor III complex; protein tyrosine kinase binding; protein complex oligomerization; positive regulation of protein localization to cell surface	919	HGNC:1677	0016020; 0007166; 0004888; 0005886; 0098797; 0005515; 0002250; 0042105; 0042802; 0050852; 0042803; 0065003; 0046982; 0005737; 0046631; 0042101; 0038094; 0046629; 0042106; 0033001; 1990782; 0051259; ; 2000010; 0005794
5 CD7	CD7 molecule	Signaling receptor activity; **adaptive immune response**; protein binding; cell surface receptor protein tyrosine kinase signaling pathway; transmembrane signaling receptor activity; T cell activation; **positive regulation of T cell cytokine production**	924	HGNC:1695	0016020; 0038023; 0002250; 0005886; 0005515; 0006955; 0007169; 0004888; 0042110; 0002726
6 CLEC6A	C-type lectin domain containing 6A	**Innate immune response; adaptive immune response;** external side of plasma membrane; protein binding; mannose binding; **positive regulation of canonical NF-kappaB signal transduction**; **positive regulation of cytokine production**; **pattern recognition receptor activity; defense response to fungus; antifungal innate immune response;** positive regulation of peptidyl-tyrosine phosphorylation; positive regulation of intracellular signal transduction; phospholipase binding; detection and response to yeast; stimulatory C-type lectin receptor signaling pathway; **positive regulation of T-helper 17 type immune response**	93978	HGNC:14556	0005886; 0005509; 0046872; 0030246; 0045087; 0002250; 0009897; 0005515; 0005537; 0043123; 0001819; 0038187; 0050832; 0061760; 0050731; 1902533; 0043274; 0001878; 0002223; 2000318; 0001879
7 DCTN6	dynactin subunit 6	Dynactin complex; centromeric region; kinetochore; centrosome; dynein complex binding; mitotic spindle organization	10671	HGNC:16964	0005869; 0005737; 0005856; 0000775; 0000776; 0005694; 0005813; 0005829; 0070840; 0007052
8 DNAJB2	DnaJ heat shock protein family (Hsp40) member B2	Unfolded protein binding; Hsp70 protein binding; chaperone-mediated protein folding; nuclear membrane; polyubiquitin modification-dependent protein binding; **negative regulation of cell population proliferation**; proteasome-mediated ubiquitin-dependent protein catabolic process; **negative regulation of cell growth**; ERAD pathway; protein refolding; regulation of protein localization; response to unfolded protein; positive regulation of protein ubiquitination; positive regulation of proteasomal ubiquitin-dependent protein catabolic process; ATPase activator activity; ubiquitin protein ligase binding; positive regulation of ATP-dependent activity; negative regulation of inclusion body assembly; **proteasome complex**; protein serine/threonine kinase binding; neuron cellular homeostasis; protein transporter activity; inclusion body; ubiquitin-modified protein reader activity; regulation of protein ubiquitination; regulation of chaperone-mediated protein folding; proteasome binding; negative regulation of protein deubiquitination	3300	HGNC:5228	0051082; 0030544; 0061077; 0031965; 0005634; 0005737; 0016020; 0005783; 0005789; 0005515; 0005829; 0031593; 0051087; 0008285; 0043161; 0030308; 0036503; 0042026; 0032880; 0006986; 0032091; 0031398; 0032436; 0001671; 0031625; 0032781; 0090084; 0000502; 0120283; 0070050; 0140318; 0016234; 0043130; 0140036; 0031396; 1903644; 0070628; 0090086
9 GOLM2	golgi membrane protein 2	Golgi apparatus	113201	HGNC:24892	0016020; 0005794

10 ICOSLG	inducible T cell costimulator ligand	Signal transduction; signaling receptor binding; **adaptive immune response**; **regulation of cytokine production; T cell receptor signaling pathway; defense response;** extracellular exosome; **B cell activation; T cell activation**; hyperosmotic response; **positive regulation of activated T cell proliferation**	23308	HGNC:17087	0016020; 0005886; 0043231; 0005515; 0007165; 0005102; 0002250; 0009897; 0042802; 0001817; 0050852; 0006952; 0070062; 0042113; 0042110; 0006972; 0042104
11 KCNG4	potassium voltage-gated channel modifier subfamily G member 4	Transmembrane transport; voltage-gated potassium channel activity; monoatomic ion transport; potassium ion transport; protein homooligomerization; voltage-gated potassium channel complex; monoatomic ion channel activity; potassium channel regulator activity; potassium channel activity; potassium ion transmembrane transport; transmembrane transporter binding; action potential; delayed rectifier potassium channel activity; regulation of potassium ion transmembrane transport	93107	HGNC:19697	0016020; 0055085; 0005249; 0006811; 0006813; 0051260; 0008076; 0005216; 0005886; 0005654; 0034702; 0015459; 0005267; 0071805; 0044325; 0001508; 0005251; 0043266; 1901379
12 LILRA5	leukocyte immunoglobulin like receptor A5	**Innate immune response; immune response-regulating signaling pathway; inhibitory MHC class I receptor activity; cytokine-mediated signaling pathway; negative regulation of interleukin-12 production; negative regulation of interleukin-13 production; positive regulation of interleukin-1 beta production; positive regulation of interleukin-10 production; positive regulation of interleukin-6 production; positive regulation of tumor necrosis factor production; positive regulation of MAPK cascade; positive regulation of inflammatory response; positive regulation of cell activation;** positive regulation of calcium ion transport; positive regulation of protein tyrosine kinase activity; extracellular space	353514	HGNC:16309	0005576; 0005886; 0045087; 0009986; 0002764; 0032396; 0019221; 0032695; 0032696; 0032731; 0032733; 0032755; 0032760; 0043410; 0050729; 0050867; 0051928; 0061098; 0005615
13 NRBP1	nuclear receptor binding protein 1	ATP binding; protein kinase activity; protein phosphorylation; cell projection; endomembrane system; protein binding; lamellipodium; cell cortex; endoplasmic reticulum to Golgi vesicle-mediated transport; protein homodimerization activity; protein serine/threonine kinase activity	29959	HGNC:7993	0005524; 0004672; 0006468; 0005737; 0016020; 0042995; 0012505; 0005515; 0030027; 0005938; 0006888; 0005654; 0042803; 0004674
14 S100A16	S100 calcium binding protein A16	Calcium ion binding; RNA binding; cytoplasm; metal ion binding; plasma membrane; protein binding; nucleolus; extracellular space; protein homodimerization activity; extracellular exosome; calcium-dependent protein binding; perinuclear region of cytoplasm; response to calcium ion	140576	HGNC:20441	0005509; 0005634; 0003723; 0005737; 0046872; 0005829; 0005886; 0005515; 0005730; 0005615; 0042803; 0070062; 0048306; 0048471; 0042802; 0051592
15 SELPLG	selectin P ligand	Cell adhesion; symbiont entry into host cell; signaling receptor binding; uropod; **virus receptor activity**; **leukocyte tethering or rolling; leukocyte migration; plasma membrane raft; leukocyte adhesive activation; cellular response to interleukin-6**	6404	HGNC:10722	0005886; 0016020; 0005515; 0007155; 0046718; 0005102; 0001931; 0001618; 0050901; 0050900; 0044853; 0050902; 0071354
16 SIRPB1	signal regulatory protein beta 1	**Positive regulation of T cell activation; signal transduction; c**ell surface receptor signaling pathway; **positive regulation of phagocytosis; cell surface; secretory granule membrane**	10326	HGNC:15928	0005886; 0005515; 0050870; 0007165; 0007166; 0050766; 0009986; 0030667; 0016020
17 SMS	spermine synthase	Spermine biosynthetic process; spermine synthase activity; transferase activity; polyamine biosynthetic process; methionine metabolic process	6611	HGNC:11123	0006597; 0016768; 0016740; 0005829; 0070062; 0006596; 0006595; 0006555; 0008215
18 TRAF4	TNF receptor associated factor 4	Metal ion binding; protein binding; zinc ion binding; **signal transduction; regulation of apoptotic process; positive regulation of JNK cascade; activation of NF-kappaB-inducing kinase activity;** ; fibrillar center; transferase activity; cytoskeleton; perinuclear region of cytoplasm; **innate immune response;** bicellular tight junction; **tumor necrosis factor receptor binding;** ubiquitin protein ligase binding; proteasome-mediated ubiquitin-dependent protein catabolic process; thioesterase binding; **regulation of canonical NF-kappaB signal transduction;** positive regulation of protein kinase activity; WW domain binding; protein kinase binding; respiratory gaseous exchange by respiratory system; respiratory tube development	9618	HGNC:12034	0046872; 0005515; 0008270; 0007165; 0042981; 0046330; 0007250; 0005634; 0005737; 0005886; 0005654; 0001650; 0005829; 0016740; 0005856; 0006915; 0048471; 0045087; 0042802; 0005923; 0019899; 0005164; 0031625; 0043161; 0031996; 0043122; 0045860; 0050699; 0019901; 0007585; 0030323

Supplementary Table 23: Function of top LPS-correlated proteins in the *LPS^{hi} Fatal* sub-group.
*Go terms of the 18 top plasma proteins correlated to plasma LPS in the LPS^{hi} Fatal sub-group (n=39). Terms related to direct immune processes have been **bolded**.*

Outcome	Term	Estimate	Std. Error	T value	P-value
PC1	(Intercept)	-6.992	3.079	-2.27	0.0235
PC1	`Plasma LPS`	-0.166	0.165	-1.01	0.315
PC1	Malaria	-7.142	2.860	-2.50	0.0128
PC1	HIV	6.214	4.120	1.51	0.132
PC1	Gastroenteritis	6.693	2.067	3.24	0.00127
PC1	Sepsis	-6.125	2.536	-2.41	0.016
PC1	LRTI	-1.598	2.052	-0.78	0.436
PC1	URTI	4.029	4.444	0.91	0.365
PC1	`Anthropometric classification`	3.238	1.157	2.80	0.00528
PC2	(Intercept)	6.433	2.418	2.66	0.008
PC2	`Plasma LPS`	-0.547	0.130	-4.21	<0.001
PC2	Malaria	-18.995	2.246	-8.46	<0.001
PC2	HIV	-11.554	3.235	-3.57	<0.001
PC2	Gastroenteritis	-0.455	1.623	-0.28	0.779
PC2	Sepsis	-2.362	1.992	-1.19	0.236
PC2	LRTI	-5.074	1.611	-3.15	0.00172
PC2	URTI	3.476	3.490	1.00	0.32
PC2	`Anthropometric classification`	1.324	0.908	1.46	0.146

Supplementary Table 24: Adjusted linear regression results for associations between plasma LPS, comorbidities, and principal components PC1 and PC2.

Linear regression models were fitted separately for PC1 and PC2 as outcome variables for admission children (n=635). Each model included plasma LPS concentration and the following comorbidities as covariates: malaria, HIV , gastroenteritis, sepsis, lower respiratory tract infection (LRTI), upper respiratory tract infection (URTI), and anthropometric classification. The table presents regression coefficients (Estimates), standard errors, t-values, and two-tailed p-values for each term in the model. Values are rounded to three decimal places, and p-values less than 0.001 are reported as "<0.001". Statistically significant associations are highlighted in green.

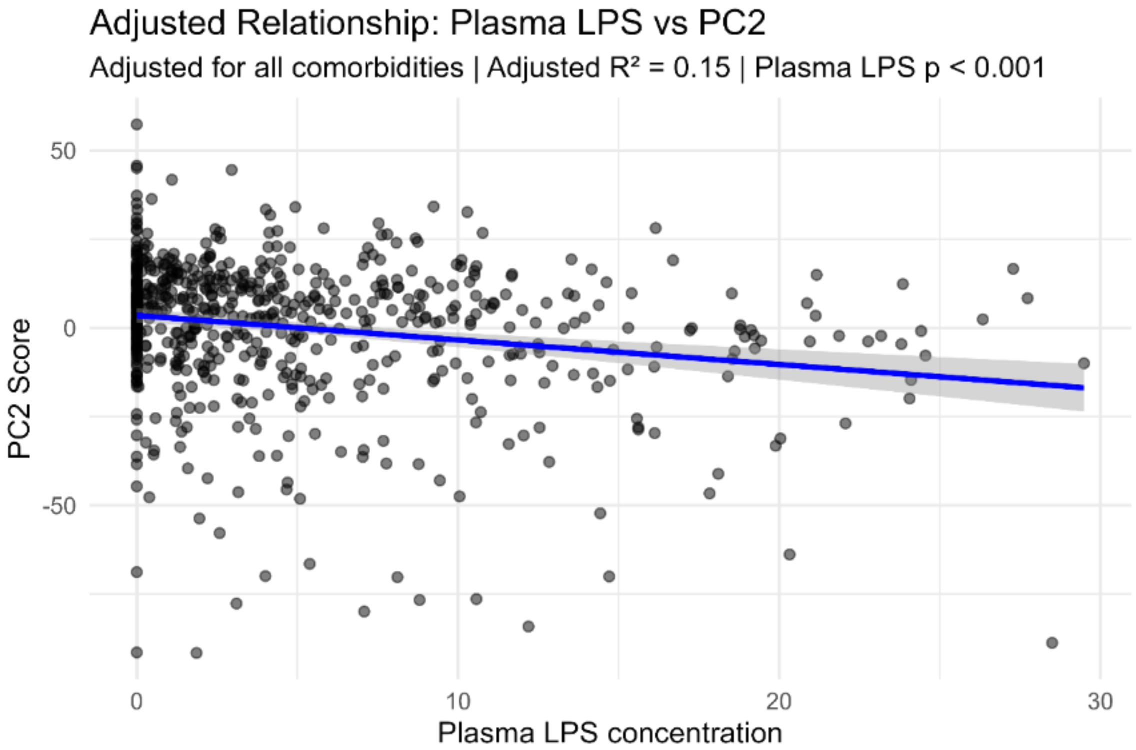

Supplementary Figure 8: Adjusted association between plasma LPS concentration and PC2.
A linear regression model was generated to examine the relationship between plasma LPS concentration and principal component 2 (PC2) of admission children (n=635) SomaScan proteomics data, adjusting for comorbidities including malaria, HIV, gastroenteritis, sepsis, lower respiratory tract infection (LRTI), upper respiratory tract infection (URTI), and anthropometric classification. Each point represents an individual participant. The blue line shows the fitted regression line with 95% confidence interval. The model yielded an adjusted R^2 *of 0.15 and a p-value for the plasma LPS coefficient of <0.001.*

Supplementary References:

1. Lew, W. Y. *et al.* Recurrent exposure to subclinical lipopolysaccharide increases mortality and induces cardiac fibrosis in mice. *PLOS ONE* **8**, e61057 (2013). https://doi.org:10.1371/journal.pone.0061057
2. Njunge, J. M. *et al.* The Childhood Acute Illness and Nutrition (CHAIN) network nested case-cohort study protocol: a multi-omics approach to understanding mortality among children in sub-Saharan Africa and South Asia. *Gates Open Res* **6**, 77 (2022). https://doi.org:10.12688/gatesopenres.13635.2
3. Mwape, I. *et al.* Immunogenicity of rotavirus vaccine (RotarixTM) in infants with environmental enteric dysfunction. *PLOS ONE* **12**, e0187761 (2017). https://doi.org:10.1371/journal.pone.0187761
4. Amadi, B. *et al.* Impaired Barrier Function and Autoantibody Generation in Malnutrition Enteropathy in Zambia. *EBioMedicine* **22**, 191-199 (2017). https://doi.org:10.1016/j.ebiom.2017.07.017
5. Kelly, P. *et al.* Gastric and intestinal barrier impairment in tropical enteropathy and HIV: limited impact of micronutrient supplementation during a randomised controlled trial. *BMC Gastroenterol* **10**, 72 (2010). https://doi.org:10.1186/1471-230X-10-72
6. Beckmann, G. T. & Rüffer, A. *Mikroökologie des Darmes: Grundlagen, Diagnostik, Therapie*. (Schlütersche, 2000).
7. Saiki, T. Myeloperoxidase concentrations in the stool as a new parameter of inflammatory bowel disease. *Kurume Med J* **45**, 69-73 (1998). https://doi.org:10.2739/kurumemedj.45.69
8. Kolho, K. L. & Alfthan, H. Concentration of fecal calprotectin in 11,255 children aged 0-18 years. *Scand J Gastroenterol* **55**, 1024-1027 (2020). https://doi.org:10.1080/00365521.2020.1794026
9. Kummerlowe, C. *et al.* Single-cell profiling of environmental enteropathy reveals signatures of epithelial remodeling and immune activation. *Science Translational Medicine* **14**, eabi8633 (2022). https://doi.org:10.1126/scitranslmed.abi8633
