# Supplementary material for: Plasma lipopolysaccharide levels predict mortality in acutely ill children in Low- and Middle-Income Countries
Source: Nat Commun. 2025 Nov 28;16:10787. doi: 10.1038/s41467-025-65429-0 (PMC12663156; doi:10.1038/s41467-025-65429-0)
Supplement: Supplementary file 2 — Reporting Summary [file 41467_2025_65429_MOESM2_ESM.pdf]

nature portfolio

Corresponding author(s): Holm H. Uhlig

Last updated by author(s): Sep 27, 2025

Reporting Summary

Nature Portfolio wishes to improve the reproducibility of the work that we publish. This form provides structure for consistency and transparency in reporting. For further information on Nature Portfolio policies, see our Editorial Policies and the Editorial Policy Checklist.

Statistics

For all statistical analyses, confirm that the following items are present in the figure legend, table legend, main text, or Methods section.

n/a	Confirmed	
☐	☒	The exact sample size (*n*) for each experimental group/condition, given as a discrete number and unit of measurement
☐	☒	A statement on whether measurements were taken from distinct samples or whether the same sample was measured repeatedly
☐	☒	The statistical test(s) used AND whether they are one- or two-sided<br>*Only common tests should be described solely by name; describe more complex techniques in the Methods section.*
☐	☒	A description of all covariates tested
☐	☒	A description of any assumptions or corrections, such as tests of normality and adjustment for multiple comparisons
☐	☒	A full description of the statistical parameters including central tendency (e.g. means) or other basic estimates (e.g. regression coefficient) AND variation (e.g. standard deviation) or associated estimates of uncertainty (e.g. confidence intervals)
☐	☒	For null hypothesis testing, the test statistic (e.g. *F*, *t*, *r*) with confidence intervals, effect sizes, degrees of freedom and *P* value noted<br>*Give P values as exact values whenever suitable.*
☒	☐	For Bayesian analysis, information on the choice of priors and Markov chain Monte Carlo settings
☒	☐	For hierarchical and complex designs, identification of the appropriate level for tests and full reporting of outcomes
☐	☒	Estimates of effect sizes (e.g. Cohen's *d*, Pearson's *r*), indicating how they were calculated

Our web collection on statistics for biologists contains articles on many of the points above.

Software and code

Policy information about availability of computer code

Data collection

This study uses previously collected data from the larger prospective, observational Childhood Acute Illness and Nutrition (CHAIN) network cohort. Data collection covers clinical (e.g., gastroenteritis, malaria, sepsis, HIV diagnosis), demographic (e.g., age, sex), anthropometric (e.g., height, weight), enteropathogen (e.g., selected bacteria, viral and parasite levels), SomaScan proteomics and survival outcome.

Data analysis

Analyses for this study were done using R Statistical software (version 4.4.2 in RStudio) and Python (version 3.10.4). R codes are provided as R Markdown (.RMD) files which allows convenient knitting to reproduce all main and supplementary results. Chunks of code can also be assessed as needed. Python codes are provided as Jupyter notebook. Panpipes (latest version) was used for this analysis.

The R packages and versions used are as follows:
Package Version
dplyr 1.1.4
gtsummary 2.0.4
cardx 0.2.2
survey 4.4.2
gt 0.11.1
here 1.0.1
data.table 1.16.2
grattan 2024.1.1
expss 0.11.6
flextable 0.9.7

survminer 0.5.0
survival 3.7.0
contsurvplot 0.2.1
ggplot2 3.5.1
rlang 1.1.4
pammtools 0.5.93
gganimate 1.0.9
transformr 0.1.5
plotly 4.10.4
reshape2 1.4.4
knitr 1.49
rmarkdown 2.29
forestplot 3.1.6
ggstats 0.7.0
cowplot 1.1.3
tidyverse 2.0.0
ggpubr 0.6.0
rstatix 0.7.2
DescTools 0.99.58
ggsci 3.2.0
weights 1.0.4
ggcorrplot 0.1.4.1
emmeans 1.11.0
gridExtra 2.3
janitor 2.2.0
rstatix 0.7.2
tibble 3.2.1
tidyr 1.3.1
caret 6.0.94
glmnet 4.1.8
ComplexHeatmap 2.22.0
circlize 0.4.16
magick 2.8.5
gridGraphics 0.5.1
grid 4.4.2
officer 0.6.7
pander 0.6.5
broom 1.0.7
broom.helpers 1.17.0
RANN 2.6.2
BiocManager 1.30.25
purrr 1.0.2

For manuscripts utilizing custom algorithms or software that are central to the research but not yet described in published literature, software must be made available to editors and reviewers. We strongly encourage code deposition in a community repository (e.g. GitHub). See the Nature Portfolio guidelines for submitting code & software for further information.

Data

Policy information about availability of data

All manuscripts must include a data availability statement. This statement should provide the following information, where applicable:

- Accession codes, unique identifiers, or web links for publicly available datasets
- A description of any restrictions on data availability
- For clinical datasets or third party data, please ensure that the statement adheres to our policy

Data Availability statement:
The data that support the findings of this study are archived on the Harvard Dataverse (https://doi.org/10.7910/DVN/EJA4F6). The data contain sensitive information about study participants and may include identifiers that could compromise confidentiality or lead to ethnic stigmatisation. Access to these data requires submission of a formal request for consideration by our Data Governance Committee. Email completed data request form to the Data Governance Committee at dgc@kemri-wellcome.org. The requester provides investigators details, variables requested, intended use of the dataset, potential risks of the study including risks to confidentiality of individuals or communities, potential benefits of the study including to participant communities, scientific capacity building or health policy and planned outputs (if analysis on dataset will result in publication or reports or presentations). The requester also needs to formally agree to the conditions and limitations for data sharing to avoid misuse of shared data.
For further details, visit: https://dataverse.harvard.edu/dataverse/kwtrp.

Data Replication:
The codes associated with this analysis can be accessed as RMD and python files through the Harvard Dataverse website under DOI: https://doi.org/10.7910/DVN/EJA4F6.

Research involving human participants, their data, or biological material

Policy information about studies with human participants or human data. See also policy information about sex, gender (identity/presentation), and sexual orientation and race, ethnicity and racism.

Reporting on sex and gender	Sex was classified based on the parent-reported 'sex assigned at birth' and recorded as a demographic factor. The admission cohort comprised 61% males, slightly higher than in community children (53%; p = 0.047). Sex distributions differed significantly across the four LPS–mortality sub-groups; therefore, sex was included as an adjustment covariate in inverse proportionally weighted CoxPH survival models (e.g., Figure 2) and in sensitivity analyses of biomarker expression (e.g., Supplementary Tables 14–15, 18–19). Although sex was independently associated with mortality in some analyses, adjustment for covariates addressed potential confounding. Individual-level data, including sex, are available in the source data to ensure reproducibility, with strict privacy safeguards in place.
Reporting on race, ethnicity, or other socially relevant groupings	The study included participants from sub-Saharan Africa and South Asia. The study does not report findings based on race or ethnicity.
Population characteristics	This study focused on exploring associations and identifying potential immune-related pathways in plasma LPS-linked mortality in 638 children admitted to hospital in 9 sites across sub-Saharan Africa and South Asia. 251 community children were incorporated in analysis as controls.<br><br>Key covariate-related population characteristics include site, continent, age, age group, sex, anthropometric classification, breastfeeding status, HIV, Malaria and TB status.<br><br>Plasma LPS concentrations did not differ between admission and community children (p > 0.9). The two cohorts also showed similar distributions of breastfeeding status, HIV, and TB (p > 0.05), with only marginal differences in sex (p = 0.047) and age (p = 0.049). By contrast, continent, anthropometric classification, and malaria history differed significantly between groups (p < 0.05). All CoxPH survival models were adjusted for these covariates, and sensitivity analyses were conducted where appropriate to account for key confounders.
Recruitment	Recruitment involved assessing criteria, approaching eligible caregivers, discussing the study, and obtaining written informed consent for children for the CHAIN cohort in South Asia and Sub-Saharan Africa. Compensation was provided for time spent at study visits according to institutional or national guidelines set to avoid undue inducement to join studies and therefore varied slightly between sites. Typically this was set at loss of earnings for the hours spent attending follow up at the national minimum wage rate along with reimbursement of transport costs.<br><br>Cases for this analysis included all acutely ill CHAIN cohort participants randomly selected in a nested case-control design who also had plasma LPS samples available. Controls were well children from the same community as cases. Inverse proportional weighting was utilized wherever possible to account for selection biases and improve generalizability of findings.
Ethics oversight	The CHAIN protocol was approved by participating sites' ethics committees and the Oxford Tropical Research Ethics Committee (OxTREC), University of Oxford.

Note that full information on the approval of the study protocol must also be provided in the manuscript.

Field-specific reporting

Please select the one below that is the best fit for your research. If you are not sure, read the appropriate sections before making your selection.

☒ Life sciences ☐ Behavioural & social sciences ☐ Ecological, evolutionary & environmental sciences

For a reference copy of the document with all sections, see nature.com/documents/nr-reporting-summary-flat.pdf

Life sciences study design

All studies must disclose on these points even when the disclosure is negative.

Sample size	The nested case-cohort study design was selected because of its ability to provide estimates that are representative of the entire cohort while also yielding slightly more statistical power than a case-control design under sample size constraints. This design involved a random 24% subsample of the original cohort (sub-cohort), followed by additional inclusion of all cases outside the sub-cohort creating the case-cohort. To determine the size of the sub-cohort, statistical power calculations suggested that a ratio of one case to two non-cases were adequate to reliably detect an HR of 1.5 at >80% power. Furthermore, simulation-based power calculations suggested that adding more non-cases per cases would yield minimal gains in power while incurring substantial additional expense. Given the expected number of cases and the results from the power calculation, a 24% random sample was determined to be optimal for the study. This sample size was however constrained by sample availability – as not all selected children had sufficient samples, and among those with sufficient samples, not all had experimental data successfully generated within the study. Therefore, LPS levels were available from 638 cohort children at admission, including 199 children that died during the study period, 439 survivors and 251 community children.
Data exclusions	Data exclusions were dependent on the objective of each analysis. Where the statistics on the entire population was performed, all 638 children admitted to hospital were included. Community participants were included as necessary as controls. Analysis of non-wasted children excluded children with MUAC < 11.5 cm. Unweighted Kaplan Meier or pathway analyses used 11.58 E.U/ml as plasma LPS cut-off based on Log-Rank Statistic of 90-day mortality. Sub-group analysis of gastroenteritis-diagnosed children excluded dysentery cases.

Replication	Codes were developed by study team members and reviewed by others when appropriate. All code was documented as R Markdown (RMD) or python files, which, along with the required input data, were independently executed at least twice. Outputs were reviewed by at least two team members, and replications consistently reproduced the results reported in the manuscript. This analysis can be replicated in fill using code and datasets available. Validation of findings will require future studies.
Randomization	No randomization was used for this study. This was a case-cohort study, and cases and non-cases were defined by the CHAIN cohort study outcome. Participants were not allocated into experimental groups, but analyses compared children who died or survived based on plasma LPS concentration.
Blinding	Investigators were blinded in all experiments.

Reporting for specific materials, systems and methods

We require information from authors about some types of materials, experimental systems and methods used in many studies. Here, indicate whether each material, system or method listed is relevant to your study. If you are not sure if a list item applies to your research, read the appropriate section before selecting a response.

Materials & experimental systems

n/a	Involved in the study	
☒	☐	Antibodies
☒	☐	Eukaryotic cell lines
☒	☐	Palaeontology and archaeology
☒	☐	Animals and other organisms
☐	☒	Clinical data
☒	☐	Dual use research of concern
☒	☐	Plants

Methods

n/a	Involved in the study	
☒	☐	ChIP-seq
☒	☐	Flow cytometry
☒	☐	MRI-based neuroimaging

Clinical data

Policy information about clinical studies

All manuscripts should comply with the ICMJE guidelines for publication of clinical research and a completed CONSORT checklist must be included with all submissions.

Clinical trial registration	ClinicalTrials.gov ID: NCT03208725
Study protocol	Njunge JM, Tickell K, Diallo AH, Sayeem Bin Shahid ASM, Gazi MA, Saleem A, Kazi Z, Ali S, Tigoi C, Mupere E, Lancioni CL, Yoshioka E, Chisti MJ, Mburu M, Ngari M, Ngao N, Gichuki B, Omer E, Gumbi W, Singa B, Bandsma R, Ahmed T, Voskuijl W, Williams TN, Macharia A, Makale J, Mitchel A, Williams J, Gogain J, Janjic N, Mandal R, Wishart DS, Wu H, Xia L, Routledge M, Gong YY, Espinosa C, Aghaeepour N, Liu J, Houpt E, Lawley TD, Browne H, Shao Y, Rwigi D, Kariuki K, Kaburu T, Uhlig HH, Gartner L, Jones K, Koulman A, Walson J, Berkley J. The Childhood Acute Illness and Nutrition (CHAIN) network nested case-cohort study protocol: a multi-omics approach to understanding mortality among children in sub-Saharan Africa and South Asia. Gates Open Res. 2022 Nov 3;6:77. doi: 10.12688/gatesopenres.13635.2. PMID: 36415883; PMCID: PMC9646488.
Data collection	Acutely ill children were recruited from nine sites in six countries in sub-Saharan Africa and South Asia. Blood, stool, and fecal swabs were collected at admission, discharge, and scheduled follow up time periods from subjects or at a single community participant visit. This study only included the admission and discharge samples.
Outcomes	Primary and Secondary outcomes are outlined in the study protocol referenced above.

Plants

Seed stocks	*Report on the source of all seed stocks or other plant material used. If applicable, state the seed stock centre and catalogue number. If plant specimens were collected from the field, describe the collection location, date and sampling procedures.*
Novel plant genotypes	*Describe the methods by which all novel plant genotypes were produced. This includes those generated by transgenic approaches, gene editing, chemical/radiation-based mutagenesis and hybridization. For transgenic lines, describe the transformation method, the number of independent lines analyzed and the generation upon which experiments were performed. For gene-edited lines, describe the editor used, the endogenous sequence targeted for editing, the targeting guide RNA sequence (if applicable) and how the editor was applied.*
Authentication	*Describe any authentication procedures for each seed stock used or novel genotype generated. Describe any experiments used to assess the effect of a mutation and, where applicable, how potential secondary effects (e.g. second site T-DNA insertions, mosiacism, off-target gene editing) were examined.*
